# Supplementary material for: Contextually appropriate communication strategies for COVID-19 prevention in Kenya border regions: evidence from a mixed methods observational study in Busia and Mandera counties
Source: BMJ Open. 2023 May 16;13(5):e062686. doi: 10.1136/bmjopen-2022-062686 (PMC10192579; doi:10.1136/bmjopen-2022-062686)
Supplement: Supplementary data [file bmjopen-2022-062686supp003.pdf]

COVID-19 RAPID QUANTITATIVE ASSESSMENT TOOL

ID of Study Participant: ……………………………………….
Date of Interview: …………………………………………….
Physical Location: …………………………………………….

**1. SOCIO-DEMOGRAPHIC INFORMATION**		
1.1	Sex	[1] Male [2] Female
1.2	Age of respondent (in complete years)	
1.3	What is your current marital status?	[1] Single (not married and not living with a partner)<br>[2] married (monogamous)<br>[3] married (polygamous)<br>[4] Separated (currently not living together but not divorced)<br>[5] Divorced<br>[6] Widowed/widower<br>[7] Co-habiting (not married but live with a partner)
1.4	What level of education or years of schooling did you finish?	[1] None<br>[2] Primary education, 1-4 years<br>[3] Primary education, 5-8 years<br>[4] Secondary Education, 9-12 years<br>[5] Secondary Education, 13-14<br>[6] University Education > 14 years<br>[7] Adult education
1.5	What is your main occupation?	[1] Student<br>[2] Employed/Professional(Teacher, doctor, nurse, manager, accountant)<br>[3] Casual worker (attendant in hotel, bar, shop, housemaid, gardener)<br>[4] Housewife<br>[5] Business (self-employed)<br>[6] Farmer (large scale, subsistent farming, gardening)<br>[7] Unemployed<br>[8]Other(specify) ___________________________
**2. SOCIO-ECONOMIC INFORMATION**		
2.1	Currently, who are you living with? (*Circle all that apply)*	[1] Alone<br>[2] Parents<br>[3] Spouse<br>[4] Children<br>[5] Other relatives<br>[6] Friends
2.2	What is the **MAIN source** of income for your household? (*Circle only one)*	[1] Formal employment<br>[2] Small scale farming<br>[3] Farming and selling of produce<br>[4] Small business, self-employed (non-farming)<br>[5] Petty trade<br>[6] Casual/informal labour<br>[7] Relatives/remittances<br>[8] Welfare/NGO support<br>[9] Other (specify)___________________________
2.3	What is the **MAIN** source of food for your	[1] Purchase (market/grocery store)<br>[2] Household farm/garden

Kaduka L, *et al. BMJ Open* 2023; 13:e062686. doi: 10.1136/bmjopen-2022-062686

	household? (*Choose one response only*)	[3] Relatives and friends<br>[4] Welfare/NGO support<br>[5] Other (specify) _____________<br>[88] Don't Know
2.4	How much on average do you spend on food in your household? (*choose from categories*)	[1] Less than 1000<br>[2] 1,000-2,999<br>[3] 3,000-4,999<br>[4] 5,000-9,999<br>[5] 10,000-19,999<br>[6] 20,000-49,999<br>[7] 50,000- 99,999<br>[8] 100, 000+
2.5	What is the main source of your domestic water?	[1] Tap/rain water within compound (piped into the house)<br>[2] Tap water outside the house compound (public tap)<br>[3] Tap water, purchased from trolley (mkokoteni)<br>[4] Well/bore hole<br>[5] Pond water<br>[6] Other (specify) ____<br>[88] Don't Know
2.6	Do you usually treat water for drinking?	[1] Yes<br>[2] No<br>[88] Don't know
2.7	If **YES to 3.6**, how do you usually treat water for drinking?	[1] Boil<br>[2] Filter<br>[3] Boil and Filter<br>[4] Use bottled water<br>[5] Do nothing<br>[6] Other (specify) ____<br>[88] Don't Know
2.8	What kind of latrine/toilet facility do members of your household usually use?	[1] Flush or pour flush toilet<br>[2] Pit latrine<br>[3] Bucket toilet<br>[4] Hanging toilet/hanging latrine<br>[5] No facility/bush/field<br>[6]Other (Specify)____
2.9	Do you share this toilet facility with other households?	[1] Yes<br>[2] No
2.10	Does your household have *(tick as many)*	[1] Clock/watch<br>[2] Electricity<br>[3] Radio<br>[4] Television<br>[5] Mobile telephone<br>[6] Fixed telephone<br>[7] Refrigerator<br>[8] Solar panel
2.11	Does any member of your household own:	[1] Bicycle<br>[2] Motorcycle/scooter<br>[3] Animal-drawn cart<br>[4] Car/truck

Kaduka L, *et al. BMJ Open* 2023; 13:e062686. doi: 10.1136/bmjopen-2022-062686

		[5] Boat with motor
2.12	What type of fuel does your household **MAINLY** use for cooking?	[1] Firewood<br>[2] Charcoal<br>[3] Kerosene<br>[4] Electricity<br>[5] Gas<br>[88] Other (Specify)_________________________________
2.13	The house you live is it,	[1] Rented<br>[2] Live without pay?<br>[3] Owns<br>[4] Squatting
2.14	How many sleeping rooms are there in the house you live?	[1] Zero or one sleeping rooms<br>[2] Two sleeping rooms<br>[3] Three or more sleeping rooms.
2.15	What quality /type of material is the floor of the house you live in?	[1] Finished floor with parquet, carpet, tiles, ceramic Tiles<br>[2] Cement, concrete, raw wood, etc<br>[3] None, earth, dung Wood<br>[4] Other (specify) ________________________
**3. KNOWLEDGE ABOUT COVID-19**		
3.1	Have you heard about the new Coronavirus disease (COVID-19)	[1] Yes<br>[2] No
3.2	What do you know about the new coronavirus disease? (Choose all that apply)	Choose one best answer:<br>**[1]** I don't know anything<br>**[2]** It's a virus that can cause a disease<br>**[3]** It's a government's programme<br>**[4]** It's a TV/radio campaign<br>**[5]** Other: Specify_________________
3.3	What kind of information have you received about the disease? (Legends to be added to the questionnaire)	Mark all answers that are true:<br>**[1]** How to protect yourself from the disease?<br>**[2]** Symptoms of the new coronavirus disease<br>**[3]** How it is transmitted<br>**[4]** What to do if you have the symptoms<br>**[5]** People who are at high risk of getting the disease<br>**[6]** Complications of the disease<br>**[7]** Other (please specify)_________

Kaduka L, *et al. BMJ Open* 2023; 13:e062686. doi: 10.1136/bmjopen-2022-062686

3.4	Where did you hear about the new coronavirus disease from? What channels or sources? (Tick all that apply)	Mark all channels that you heard about the disease from:<br>**[1]** Radio<br>**[2]** TV<br>**[3]** WhatsApp<br>**[4]** Social Media (e.g. Facebook, Instagram, Twitter)<br>**[5]** Health unit/Healthcare worker<br>**[6]** Family members<br>**[7]** Friends<br>**[8]** Community health workers<br>**[9]** Red Cross/ Red Crescent volunteers<br>**[10]** Other community mobilisers<br>[11] Community leaders<br>[12] Religious Leaders<br>[13] Traditional Healers<br>[14] Traditional Midwives<br>[15] Any other person from the community<br>[16] Other (please specify) __________
3.5	Which channel/who do you trust the most to receive information related to coronavirus? (one or more options)	Check the best channel for you to receive coronavirus information from:<br>**[1]** Radio<br>**[2]** TV<br>**[3]** WhatsApp<br>**[4]** Social Media (not WhatsApp)<br>**[5]** Health unit/Healthcare worker<br>**[6]** Family members<br>**[7]** Friends<br>**[8]** Community health workers<br>**[9]** Red Cross Red Crescent volunteers<br>**[10]** Other community mobilisers<br>[11] Community leaders<br>[12] Religious Leaders<br>[13] Traditional Healers<br>[14] Traditional Midwives<br>[15] Any other person from the community<br>[16] Other (Specify) ______________________
3.6	How dangerous do you think the new coronavirus risk is?	Choose the best answer that matches your belief about the disease:<br>**[1]** Very dangerous<br>**[2]** More or less dangerous<br>**[3]** Is not dangerous<br>**[4]** Other (Specify)________________________
3.7	Who do you think is at highest risk to get the coronavirus?	

Kaduka L, *et al. BMJ Open* 2023; 13:e062686. doi: 10.1136/bmjopen-2022-062686

		Mark all those you think are likely to become sick:<br>**[1]** Children under 5 years old<br>**[2]** Adolescents up to 15 years old<br>**[3]** Youth<br>**[4]** Adults<br>**[5]** Elderly persons<br>**[6]** Pregnant women<br>**[7]** Health workers<br>**[8]** Persons with chronic diseases such as diabetes and high blood pressure<br>[**9**] Other (Specify)________________
3.8	Do you think you are likely to become sick with the new coronavirus?	[1] Yes<br>[2] No<br>[3] Don´t know<br>If Yes or No, tell us Why_
3.9	Do you think any of your family members are at risk of becoming sick with coronavirus?	[1] Yes<br>[2] No<br>[3] Don´t know<br>If Yes or No, tell us Why_
3.10	How does the coronavirus spread?	Mark all the ways you think the disease spreads:<br>**[1]** Blood transfusion<br>**[2]** Droplets from infected people<br>**[3]** Airborne<br>**[4]** Direct contact with infected people.<br>**[5]** Touching contaminated objects/surfaces<br>**[6]** Sexual intercourse contact<br>**[7]** Contact with contaminated animals<br>**[8]** Mosquito bites<br>**[9]** Eating contaminated food<br>**[10]** Drinking unclean water<br>**[11]** Don't know<br>[12] Other (Specify)________________
3.11	What are the main symptoms of COVID-19?	Mark all the symptoms you think are caused by the new coronavirus:<br>**[1]** Fever<br>**[2]** Cough<br>**[3]** Shortness of breath and breathing difficulties<br>**[4]** Muscle pain<br>**[5]** Headache<br>**[6]** Diarrhoea<br>**[7]** Don't know<br>**[8]** No symptoms<br>**[9]** Other (Specify) ________________________

Kaduka L, *et al. BMJ Open* 2023; 13:e062686. doi: 10.1136/bmjopen-2022-062686

3.12	Do you know how to prevent it? (One or more options)	Choose all the prevention steps you know about:<br>**[1]** Sleep under the mosquito net<br>**[2]** Wash your hands regularly using soap and water<br>[3] Use hand sanitiser<br>**[4]** Drink only treated water<br>**[5]** Cover your mouth and nose when coughing or sneezing<br>**[6]** Avoid close contact with anyone who has a fever and cough<br>**[7]** Eliminate standing water<br>**[8]** Cook meat and eggs well<br>**[9]** Avoid unprotected direct contact with live animals and surfaces in contact with animals<br>**[10]** Standing 2 metres away from other people<br>**[11]** Wearing a mask<br>**[12]** Don't know<br>**[13]** Other Specify_________________________
3.13	What have you and your family been doing to prevent becoming sick with coronavirus in the recent days? (Tick all that apply)	Mark all steps that you have taken:<br>**[1]** Washing hands regularly using soap / water<br>[2] Using alcohol-based hand sanitiser<br>**[3]** Covering mouth and nose when coughing or sneezing<br>**[4]** Avoid close contact with anyone who has a fever and cough<br>**[5]** Eliminate standing water<br>**[6]** Cook meat and eggs well<br>**[7]** Avoid unprotected direct contact with live animals and surfaces in contact with animals.<br>**[8]** Standing 2 metres away from other people<br>**[9]** Wearing a mask<br>**[10]** Don't know<br>**[11]** Nothing<br>**[12]** Other (Specify)________________________________
3.14	If Nothing to question 3.13, what makes it difficult for you to follow advice on preventing yourself from the disease?	**[1]** Need to go to work to earn money<br>**[2]** Live with a large family in a small house<br>**[3]** Don't know<br>**[4]** Other (Please specify)____________________
3.15	Do you consider it important to take actions to prevent the spread of coronavirus in your community?	[1] Yes<br>[2] No<br>[3] Don't know<br>[4] Other (Specify) ______________________
3.16	What would you do if you or someone from your family has symptoms of this disease?	

		Tick all the actions you would take:<br>**[1]** I will look for a more experienced relative to advise me on what to do<br>**[2]** I will go to the hospital / health unit<br>**[3]** I will go to the neighbourhood nurse<br>**[4]** I will buy medicines at the market<br>**[5]** I will look for the traditional healer<br>**[6]** I would stay in quarantine<br>**[7]** Other (Specify) ______________________________
3.17	What more would you like to know about the disease?	Choose all topics that you would like to know about;<br>**[1]** How to protect yourself from the disease?<br>**[2]** Symptoms of the new coronavirus disease<br>**[3]** How it is transmitted<br>**[4]** What to do if you have the symptoms<br>**[5]** Most at risk groups<br>**[6]** How to treat it<br>**[7]** Other (Specify)___________________________
3.18	Do you think the coronavirus disease is generating stigma against specific people?	[1] Yes<br>[2] No<br>[3] Don't know
3.19	(If yes) Which group is being dis-criminated in your community because of coronavirus?	1.____________________________________<br>2.____________________________________<br>3.____________________________________
3.20	Do you think Kenya is adequately prepared in dealing with this disease?	[1] Yes<br>[2] No<br>[3] Don't know
3.21	Do you know of any special measures that have been put in place nationally to deal with this new coronavirus? (Tick all that apply)	[1] Social distancing<br>[2] Closing borders<br>[3] Coronavirus telephone line/help<br>[4] Temporary expansion of healthcare facilities<br>[5] National Curfew<br>[6] Other (please specify)
3.22	If restrictions are in place, have you had difficulty in acquiring essential supplies such as food?	[1] Yes<br>[2] No<br>[3] Don't know
3.23	How do you think vulnerable groups can access essential supplies such as food and medicine? (Tick all that apply)	(1). Friends and family<br>(2). Community programmes<br>(3). National government run service<br>(4). NGO<br>(5). Religious organisations<br>(6). Other (please specify)

Kaduka L, *et al. BMJ Open* 2023; 13:e062686. doi: 10.1136/bmjopen-2022-062686

4. KNOWLEDGE ABOUT SIMILAR DISEASES: SEASONAL OR REGULAR FLU		
4.1	What do you usually do if you have a regular flu?	Mark the 1-2 most typical actions you take:<br>**[1]** I have never had the flu<br>**[2]** Nothing, I continue with my normal life, after some time the flu goes away<br>**[3]** I stay at home, so I don't infect others<br>**[4]** I wear a mask<br>**[5]** I drink tea until I recover<br>**[6]** I seek advice from my neighbours<br>**[7]** I seek advice from local healers<br>**[7]** I go to the health unit<br>**[8]** Other (Specify) ________________________________
4.2	From where/who do you usually receive information on flu and other diseases?	Choose as many channels as you have heard about regular flu from:<br>**[1]** Radio<br>**[2]** TV<br>**[3]** WhatsApp<br>**[4]** Social Media (not WhatsApp)<br>**[5]** Health unit/Healthcare worker<br>**[6]** Family members<br>**[7]** Friends<br>**[8]** Community health workers<br>**[9]** Red Cross Red Crescent volunteers<br>**[10]** Other community mobilisers<br>[11] Community leaders<br>[12] Religious Leaders<br>[13] Traditional Healers<br>[14] Traditional Midwives<br>[15] Any other person from the community<br>[16] Other (Specify) ____________________

THANK YOU VERY MUCH FOR YOUR TIME

Kaduka L, *et al. BMJ Open* 2023; 13:e062686. doi: 10.1136/bmjopen-2022-062686
