# Supplementary material for: Contextually appropriate communication strategies for COVID-19 prevention in Kenya border regions: evidence from a mixed methods observational study in Busia and Mandera counties
Source: BMJ Open. 2023 May 16;13(5):e062686. doi: 10.1136/bmjopen-2022-062686 (PMC10192579; doi:10.1136/bmjopen-2022-062686)
Supplement: Supplementary data [file bmjopen-2022-062686supp005.pdf]

Supplementary Table 1: List of variables used for constructing a wealth index

	Name of Variable	Coding		Factor loadings	
				Factor1	Factor2
1	Ownership of household goods - 1 Clock/Watch	1 = Yes	0 = No		
2	Ownership of household goods - 2 Electricity	1 = Yes	0 = No	.708	
3	Ownership of household goods - 3 Radio	1 = Yes	0 = No		.657
4	Ownership of household goods - 4 Television	1 = Yes	0 = No	.628	.427
5	Ownership of household goods - 5 Mobile telephone	1 = Yes	0 = No	.375	
6	Ownership of household goods - 6 Fixed telephone	1 = Yes	0 = No		
7	Ownership of household goods - 7 Refrigerator	1 = Yes	0 = No		
8	Ownership of household goods - 8 Solar panel	1 = Yes	0 = No		.552
9	Any household member own - 1 Bicycle	1 = Yes	0 = No		.753
10	Any household member own - 2 Motorcyle/Scotter	1 = Yes	0 = No	.427	
11	Any household member own - 3 Animal drawn cart	1 = Yes	0 = No		
12	Any household member own - 4 Car/Truck	1 = Yes	0 = No		
13	Any household member own - 5 Boat with motor	1 = Yes	0 = No		
14	Own house live in	1 = Yes	0 = No		.346
15	Sleeping rooms in the house that live in	1, 2, 3		.640	
16	Finished/cement floor	1 = Yes	0 = No	.681	
	Small coefficients below .30 were suppressed<br>*Eigen values for components one and two were 2.464 & 1.509*				
