# Supplementary material for: Contextually appropriate communication strategies for COVID-19 prevention in Kenya border regions: evidence from a mixed methods observational study in Busia and Mandera counties
Source: BMJ Open. 2023 May 16;13(5):e062686. doi: 10.1136/bmjopen-2022-062686 (PMC10192579; doi:10.1136/bmjopen-2022-062686)
Supplement: Supplementary data [file bmjopen-2022-062686supp004.pdf]

COVID-19 QUALITATIVE INTERVIEW GUIDE

This will be a semi structured interview with people who have knowledge of and experienced the COVID-19 disease. We wish to obtain views/opinions from policy actors, healthcare workers, COVID-19 survivors and carers, COVID-19 bereaved, truckers and traders.

Introductory Script

Enumerator:
Good morning/ afternoon,
My name is {**Enumerator Name**} from **KEMRI**.
Welcome to this session and thank you for taking the time to be here. We are conducting a research study on behalf of KEMRI and Kings College London (UK) to assess the response strategies and community/practitioner experiences of COVID-19 in border counties in Kenya.

We would like you to be as honest and as open as you can. Also take note that there are no correct, right or wrong response(s) to the issues raised. Feel free to chip in or interject at any point. All your responses will be both confidential and anonymous, and your name and details will not be divulged during analysis. Your responses will not affect your standing with anyone or any organization/company mentioned in this discussion.

In order to make sure that we capture the most useful account of this discussion, we would like to audio record our conversation. This recording will only be available to the research team involved. Will you give us permission to record?
Thank you.

Demographic Information

1. Participant geographic Location
2. Sex
3. Age
4. Occupation
5. Level of Education

Awareness of COVID-19

1. Have you heard about the New Coronavirus Disease (COVID-19)? Please tell me what you know about it (*Explain to the participant what the disease is in case s/he doesn't know what it is*)
2. From whom/ where did you get this information from (*Prompt for details of the information given through the specified source e.g. symptoms, transmission, self-care, risks, complications etc.*)

3. Are there some people, information sources or channels that you trust more than others to give you accurate information about the disease? Which people or sources are these and why do you trust them?

4. In your view how dangerous do you think the COVID-19 disease is? (*Probe for reasons as to why*)

5. Who in your view do you think is most at risk of contracting this disease? (*Probe for reasons as to why*)

6. Please explain to me how you think the COVID-19 is spread in in your County?

7. In your view, how would you identify someone who is infected with the disease? (*Probe for main symptoms*)

8. Do you think a person can be infected and spread the infection to others before they start showing the symptoms you have just mentioned? (*probe for reason for their opinion*)

9. Have you heard of any test that is done by medical professionals to identify people who have the disease? (*probe for what they know about testing*)

10. How would you prevent yourself or your family from contracting this disease?

11. What have you been doing to prevent yourself and your family from contracting the disease? (*Probe for prevention measures*)

12. What role can you play to prevent the spread of the disease within your community? (*Probe for self-quarantine measures, creation of awareness*)

13. What would you do if a member of your family contracted the disease?

14. Where do you think would be the best place for your family member to receive treatment if they contracted the disease? If it is a place away from home, would you be able to take them there? (*probe for why this is so both yes and no answers*)

15. What myths and misconceptions have you heard about the disease? (Probe for myths and misconceptions around symptoms, transmission, prevention, treatment)

16. Is there any more information you would like to know about the disease? (*Probe for preferred source for the additional information*)

17. In your view, do you think the COVID-19 disease pandemic is generation stigma against specific people? (*Probe the specific groups of people being stigmatised and how they are being stigmatized*)

18. Have you ever been sick with seasonal or regular flu? What do you usually do when you have a seasonal flu? (*Probe for home remedies, seeking care from hospital, doing nothing about it etc.*)

Kaduka L, *et al. BMJ Open* 2023; 13:e062686. doi: 10.1136/bmjopen-2022-062686

Additional questions for policy actors and healthcare workers

19. What is your role in the current organization that you serve?
20. How would you describe the preparedness and response of Kenya's healthcare system to COVID 19?
21. What has been your personal experience in regard to COVID-19?
22. What are some of the facilitators and barriers in the healthcare system when it comes to dealing with COVID-19 pandemic?
23. What would you wish to see done differently?

Thank you very much for your time and response.

Kaduka L, *et al. BMJ Open* 2023; 13:e062686. doi: 10.1136/bmjopen-2022-062686
