# Supplementary material for: Contextually appropriate communication strategies for COVID-19 prevention in Kenya border regions: evidence from a mixed methods observational study in Busia and Mandera counties
Source: BMJ Open. 2023 May 16;13(5):e062686. doi: 10.1136/bmjopen-2022-062686 (PMC10192579; doi:10.1136/bmjopen-2022-062686)
Supplement: Supplementary data [file bmjopen-2022-062686supp001.pdf]

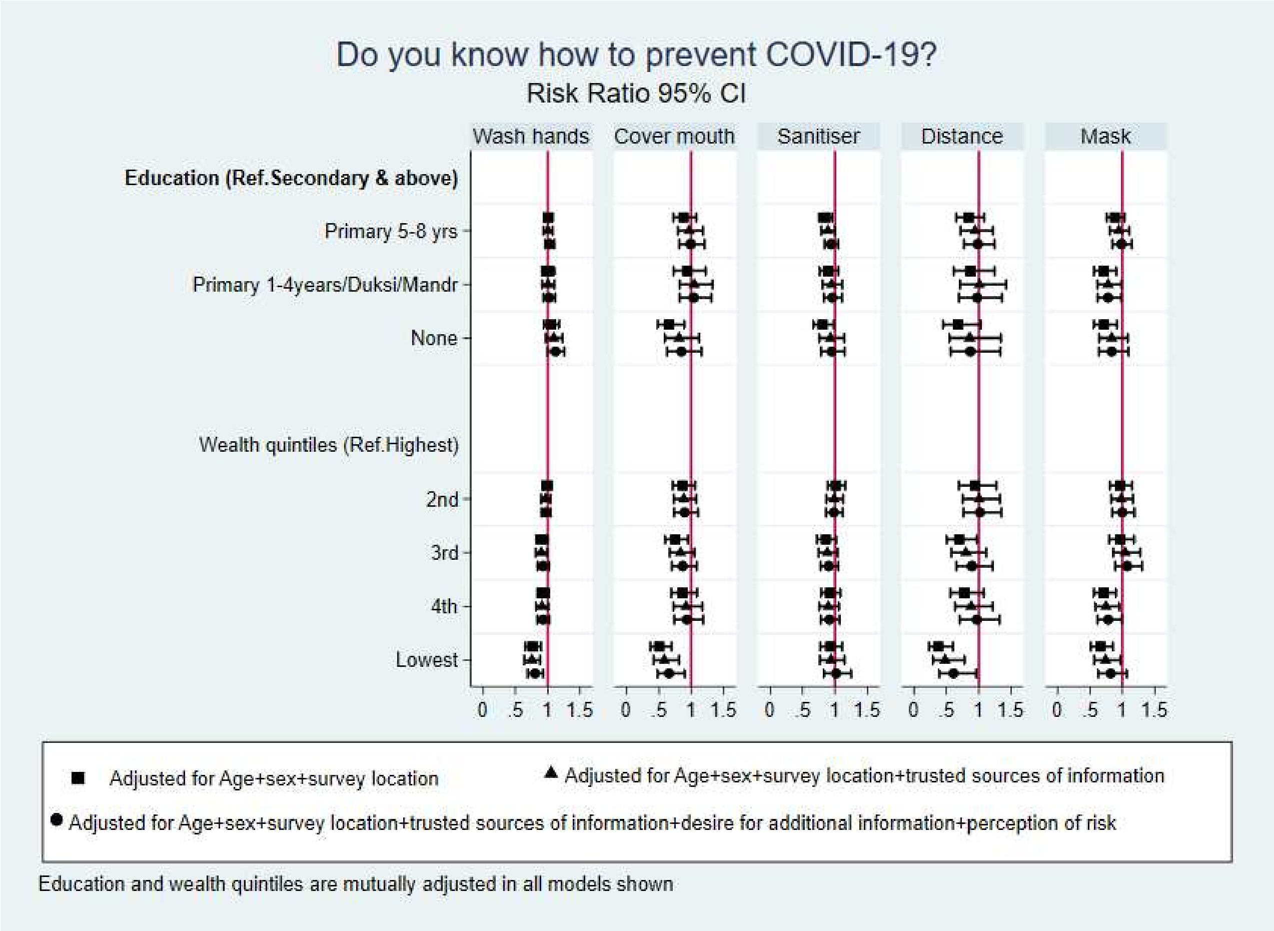

Supplementary Figure 1: Association between knowledge of prevention behaviours and measures of education and wealth. Risk Ratio and 95% confidence intervals.
