# Supplementary material for: Contextually appropriate communication strategies for COVID-19 prevention in Kenya border regions: evidence from a mixed methods observational study in Busia and Mandera counties
Source: BMJ Open. 2023 May 16;13(5):e062686. doi: 10.1136/bmjopen-2022-062686 (PMC10192579; doi:10.1136/bmjopen-2022-062686)
Supplement: Supplementary data [file bmjopen-2022-062686supp002.pdf]

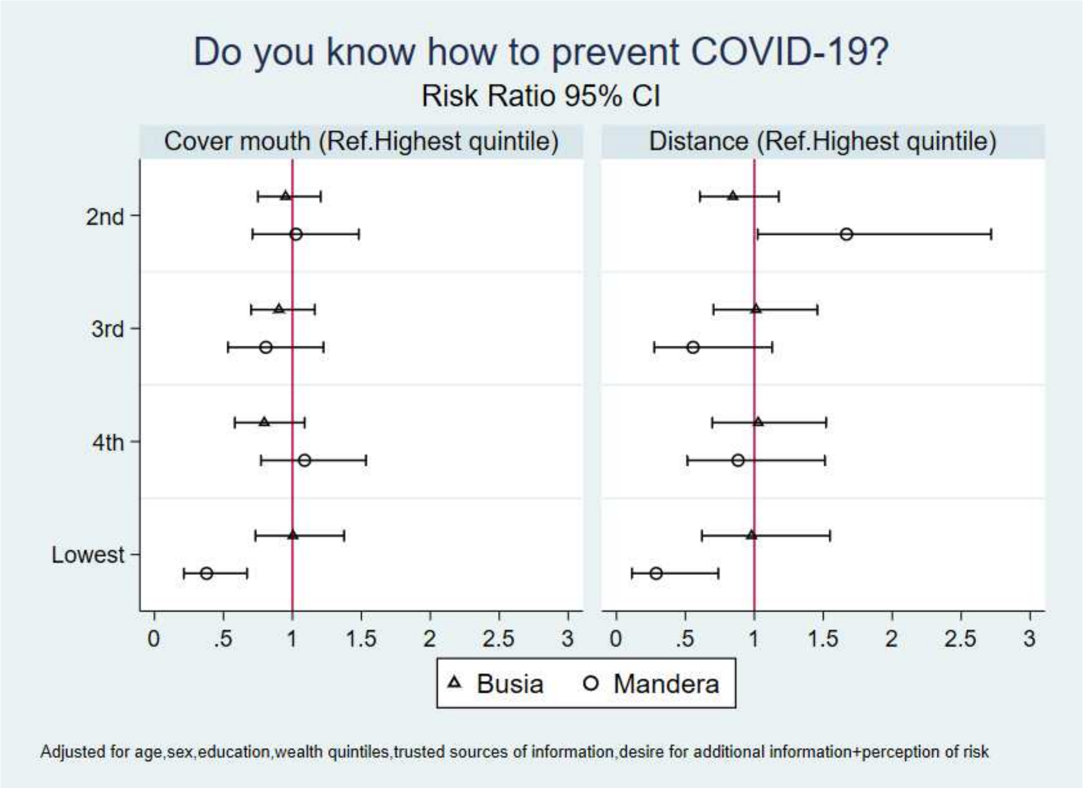

Supplementary Figure 2: Interaction between wealth quintiles and survey area for knowing that covering mouth when coughing/sneezing and keeping 2 metres distance could prevent COVID-19.
