# Supplementary material for: Contextually appropriate communication strategies for COVID-19 prevention in Kenya border regions: evidence from a mixed methods observational study in Busia and Mandera counties
Source: BMJ Open. 2023 May 16;13(5):e062686. doi: 10.1136/bmjopen-2022-062686 (PMC10192579; doi:10.1136/bmjopen-2022-062686)
Supplement: Author's manuscript [file bmjopen-2022-062686.draft_revisions.pdf]

BMJ Open is committed to open peer review. As part of this commitment we make the peer review history of every article we publish publicly available.

When an article is published we post the peer reviewers' comments and the authors' responses online. We also post the versions of the paper that were used during peer review. These are the versions that the peer review comments apply to.

The versions of the paper that follow are the versions that were submitted during the peer review process. They are not the versions of record or the final published versions. They should not be cited or distributed as the published version of this manuscript.

BMJ Open is an open access journal and the full, final, typeset and author-corrected version of record of the manuscript is available on our site with no access controls, subscription charges or pay-per-view fees (http://bmjopen.bmj.com).

If you have any questions on BMJ Open's open peer review process please email info.bmjopen@bmj.com

BMJ Open

Contextually Appropriate Communication Strategies for COVID-19 prevention in Kenya Border Regions: Evidence from an observational study in Busia and Mandera Counties.

Journal:	*BMJ Open*
Manuscript ID	bmjopen-2022-062686
Article Type:	Original research
Date Submitted by the Author:	11-Mar-2022
Complete List of Authors:	Kaduka, Lydia; Kenya Medical Research Institute, Centre for Public Health Research<br>Olale, Joanna; Kenya Medical Research Institute<br>Karamanos, Alexis; King's College London Faculty of Life Sciences and Medicine<br>Mutai, Joseph; Kenya Medical Research Institute<br>Coultas, Clare; King's College London School of Medical Education<br>Ahmed, Ismail; Kenya Medical Research Institute<br>L'Esperance, Veline; King's College London, School of Population Health and Environmental Sciences<br>Read, Ursula; University of Warwick Warwick Medical School<br>Dazzan, Paola; King's College London Institute of Psychiatry Psychology and Neuroscience<br>Cruickshank, John; King's College London Faculty of Life Sciences and Medicine, School of Life-course and Nutritional Sciences<br>Muniu, Erastus; Kenya Medical Research Institute<br>Harding, Seeromanie; King's College London Faculty of Life Sciences and Medicine
Keywords:	COVID-19, EPIDEMIOLOGY, Public health < INFECTIOUS DISEASES, PREVENTIVE MEDICINE

SCHOLARONE™
Manuscripts

I, the Submitting Author has the right to grant and does grant on behalf of all authors of the Work (as defined in the below author licence), an exclusive licence and/or a non-exclusive licence for contributions from authors who are: i) UK Crown employees; ii) where BMJ has agreed a CC-BY licence shall apply, and/or iii) in accordance with the terms applicable for US Federal Government officers or employees acting as part of their official duties; on a worldwide, perpetual, irrevocable, royalty-free basis to BMJ Publishing Group Ltd ("BMJ") its licensees and where the relevant Journal is co-owned by BMJ to the co-owners of the Journal, to publish the Work in this journal and any other BMJ products and to exploit all rights, as set out in our licence.

The Submitting Author accepts and understands that any supply made under these terms is made by BMJ to the Submitting Author unless you are acting as an employee on behalf of your employer or a postgraduate student of an affiliated institution which is paying any applicable article publishing charge ("APC") for Open Access articles. Where the Submitting Author wishes to make the Work available on an Open Access basis (and intends to pay the relevant APC), the terms of reuse of such Open Access shall be governed by a Creative Commons licence – details of these licences and which Creative Commons licence will apply to this Work are set out in our licence referred to above.

Other than as permitted in any relevant BMJ Author's Self Archiving Policies, I confirm this Work has not been accepted for publication elsewhere, is not being considered for publication elsewhere and does not duplicate material already published. I confirm all authors consent to publication of this Work and authorise the granting of this licence.

For peer review only

Contextually Appropriate Communication Strategies for COVID-19 prevention in Kenya Border Regions: Evidence from an observational study in Busia and Mandera Counties

Kaduka Lydia, Olale Joanna, Karamanos Alexis, Mutai Joseph, Coultas Clare, Ahmed Ismail, Lesperance Veline, Read Ursula, Dazzan Paola, Cruickshank Kennedy, Muniu Erastus, Harding Seeromanie.

Kaduka Lydia, Centre for Public Health Research, Kenya Medical Research Institute, Nairobi, Kenya.

Olale Joanna, Centre for Clinical Research, Kenya Medical Research Institute, Nairobi, Kenya.

Karamanos Alexis, School of Population Sciences and Health Services Research and School of Life Course Sciences, King's College London, London, UK.

Mutai Joseph, Centre for Public Health Research, Kenya Medical Research Institute, Nairobi, Kenya.

Coultas Clare, School of Education, Communication and Society, King's College London, London, UK.

Ahmed Ismail, Centre for Public Health Research, Kenya Medical Research Institute, Nairobi, Kenya.

Lesperance Veline, School of Population Sciences and Health Services Research and School of Life-Course Sciences, King's College London, London, UK.

Read Ursula, Department of Mental Health and Wellbeing, Warwick Medical School, University of Warwick, Coventry, UK.

Dazzan Paola, Department of Psychological Medicine, Institute of Psychiatry, Psychology and Neuroscience, King's College London, London, UK.

Cruickshank Kennedy, School of Life-course and Nutritional Sciences, Kings College London, London, UK.

Muniu Erastus, Centre for Public Health Research, Kenya Medical Research Institute, Nairobi, Kenya.

Harding Seeromanie, School of Population Sciences and Health Services Research and School of Life Course Sciences, King's College London, London, UK.

Corresponding author

Kaduka Lydia

Kenya Medical Research Institute

For peer review only - http://bmjopen.bmj.com/site/about/guidelines.xhtml

Centre for Public Health Research

P.O. Box 20752-00202, Nairobi, Kenya

Tel: (254)-2-2725016/7. Fax: (254)-2-2720030

Email: lkaduka@kemri.go.ke

Keywords: COVID-19, Border regions, Kenya

Word Count: 4110

For peer review only

For peer review only - http://bmjopen.bmj.com/site/about/guidelines.xhtml

ABSTRACT

Objectives: Kenya has long and porous borders with its neighbouring countries. These regions, predominantly inhabited by highly mobile rural communities with strong cross-border cultural ties, present major challenges in managing movement of people and COVID-19 preventive measures. Our study sought to assess knowledge of COVID-19 prevention behaviours, how these varied by socio-economic (SEC) factors, and the challenges of engagement and implementation, in two border counties of Kenya.

Methods: We conducted a mixed methods study using a household e-survey (Busia N=294; Mandera N=288; 57% females, 43% males), and qualitative telephone interviews (N=73: Busia 55; Mandera 18) with policy actors, healthcare workers, truckers and traders, and community members. Interviews were transcribed, English-translated and analysed using the Framework Method. Associations between SEC (wealth quintiles, educational level) and knowledge of COVID-19 preventive behaviours were explored using Poisson regression.

Results: Participants were mostly educated to primary school level (54.4% Busia, 61.6% Mandera). Knowledge of COVID-19 prevention varied by behaviour: hand washing - 86.5%, use of hand sanitiser -74.8%, wearing a face mask - 63.1%, covering the mouth when sneezing or coughing -56.3% and social distancing- 40.1%. Differences in knowledge by area, educational level and the wealth index were marked, greatest for Mandera, the less educated and the poor. Interviews with stakeholders revealed challenges in health messaging, psychosocial and socioeconomic factors, lack of preparedness for truck border crossings, language barrier, denial and livelihood insecurity as key challenges to engagement with and implementation of COVID-19 prevention behaviours in the border regions.

Conclusion: The influence of SEC disparities and border dynamics on knowledge and engagement with COVID-19 prevention behaviours call for contextually appropriate risk communication strategies that are cognisant of community needs and local patterns of information flow. Coordinating response measures across border points is crucial in winning communities' trust and maintaining essential economic and social activities.

For peer review only - http://bmjopen.bmj.com/site/about/guidelines.xhtml

STRENGTHS AND LIMITATIONS

- The study sampling strategy and high e-survey response rate (97%) were key strengths, which point to the versatility required in the conduct of research in emergency response with community engagement being central to the success of similar research projects.
- The study did not objectively assess the practice of preventive behaviours. To overcome the potential bias of self-reported information, qualitative interviews were used to explore factors influencing the ability of the community to adhere to preventive measures.
- Whereas Kenya's median age is 21, majority of the e-survey participants were ≥55 years. This group tends to be less tech savvy and may explain the trust hierarchy observed in this study.
- The study recorded a lower number of Mandera interviews. However, the context-specific challenges presented and what may be considered socio-culurally appropriate recommendations considerably inform future local efforts.

INTRODUCTION

Evidence continues to accrue on the dramatic global direct and indirect impacts of the COVID-19 pandemic. The pandemic has put a toll on both the social and economic structures of societies, contributing to global social, economic and health disparities [1,2]. Kenya has a population of ≈48 million, median age 20.1 years and 66.2% living on <US $3.20 /day [3]. It has long and porous borders with Tanzania, Uganda, Ethiopia, South Sudan, and Somalia, predominantly inhabited by highly mobile rural communities with strong cross-border cultural ties. Eight weeks after the first reported COVID-19 case in Kenya on March 13th 2020, a quarter of all COVID-19 reported cases came from these border counties. Recognising its vulnerability to COVID-19 due to its position as a major travel and transport hub and relatively weak health system, Kenya implemented several mitigation measures including: a dusk to dawn curfew; mandatory mask wearing; prohibition of large public gatherings; and closure of borders [4,5]. Indirect impacts of the measures resulted in increased poverty levels, disrupted livelihoods, deepened inequalities and decelerated progress towards the Sustainable Development Goals [6-8].

The needs of communities at border regions are an important consideration for Kenya and its neighbours due to their shared vision of regional health security. The porous borders in the Mandera triangle (Kenya, Ethiopia, and Somalia borders) and Busia (Kenya -Uganda border) limits the ability of partner states to monitor and identify active cases during pandemics [9-14]. With only 18% of the Kenyan population fully vaccinated by January 19th 2022 [15], the mainstay of the response remains adoption of preventive behaviours to reduce transmission.

For peer review only - http://bmjopen.bmj.com/site/about/guidelines.xhtml

In this study, we aimed to assess the levels of knowledge of key COVID-19 prevention behaviours, and whether these varied by socioeconomic factors, and obtained qualitative narratives to illuminate contextual factors impacting on engagement with prevention measures in Busia and Mandera border counties of Kenya. These two border counties continue to record poorer health outcomes due to long-standing issues in accessing reasonable quality healthcare because of poor health infrastructure, remoteness from the capital city, and high poverty rates. They are also among the eight lowest ranked counties in Kenya with the least healthcare workers per capita [16].

METHODS

Study design

A convergent parallel mixed methods study design was used to collect both quantitative and qualitative data in the two counties between September and October 2020.

Study settings: Mandera County located in Northern Kenya shares a border of 861km with Ethiopia to the North and 682 km with Somalia to the East (Figure 1). It is located 1052km from Kenya's capital city Nairobi, covers an area of 25,798 km², has a population of 867,457, population density of 33 persons/km², high poverty level (77.6%), and is inhabited predominantly by Muslim Somalis. Mandera's main economic activities are pastoralism and cross-border trade. Of Mandera's 775,085 population aged >3 years, 72% have never been to a learning institution. Busia County located 475km from Nairobi, borders Lake Victoria to the North and Uganda to the West (total border distance, 772 Km). With a 1,700Km² area and population of 893,000, population density is 527 persons/km², and poverty level 69%. Its main economic activities are cross-border trade, fishing, and agriculture, and is inhabited predominantly by Christians. 12.5% of Busia's 820,788 population aged >3 years have never been to school [17].

Quantitative survey

Sample size and sampling procedure: A cross-sectional household e-survey targeting adults aged >18 years was undertaken in September 2020. Sample size calculation was based on a prevalence of 50% for the indicator of interest (knowledge) and a maximum relative standard error for the estimated proportion of 7% to give a sample size of 205. Assuming 20 participants per cluster (communities), an intra-class correlation coefficient between clusters of 0.01, and a non-response rate of 10%, the resulting sample size was 269 rounded off to 300 participants, equivalent to 15 clusters per county. A three-stage sampling process was used. The first stage involved simple random sampling of Community Health Units (CHUs) in each County. The second stage involved sampling households within CHUs using systematic sampling with a random start, and lastly respondent selection in sampled households using the Kish Grid method [18].

For peer review only - http://bmjopen.bmj.com/site/about/guidelines.xhtml

Data collection and analysis: The e-survey data was collected using the WHO COVID-19 Rapid Quantitative Assessment Tool adapted to the local context [19]. The study used the REDCap software [20] to automate and share the survey tool with study participants.

Outcome measures: Knowledge about COVID-19 related preventive health behaviours was assessed by asking survey participants the following question *"Do you know how to prevent COVID-19?"*, to which they responded with a yes/no on the following individual statements; prevent COVID-19 by (i) wearing a face mask, (ii) keeping a distance of 2 metres from others, (iii) covering the mouth and nose when sneezing or coughing, (iv) using a hand sanitiser, and (v) washing hands with soap and water regularly. Affirmative responses were categorised as the reference category. Exposure status was assessed by asking participants if they had experienced any of the following symptoms - fever, cough, shortness of breath, sore throat, fatigue, loss of taste or smell, or eye infection, two weeks prior to the survey.

Correlates and confounders: Socio-economic circumstances (SEC) were measured using education and the wealth index, two commonly used SEC indices in Kenya [17]. The wealth index was calculated using data on household's ownership of 14 selected assets (Supplementary Table 1). The weights (factor scores) for each of the assets were generated through principal components analysis (PCA). Years of education and adult education were categorised as following; 1) Secondary education and above (more than 9 years of compulsory years of education), 2) upper primary education (5-8 years of compulsory education), 3) lower primary education (1-4 years of compulsory education) or/and Islamic education (Duksi or Madrassa), and 4) no education.

Participant's sex, age (categorised in 4 age bands 18-24, 25-54, 55-65 and 65+) and survey location (Mandera/Busia) were considered as potential confounders. Trusted sources of information, desire for additional information on COVID-19 and perception of risks stemming from SARS CoV2 were considered as potential mediators and were assessed by asking participants to respond with a yes/no to each category for the following questions: *"Which source/who do you trust the most to receive information related to the new Coronavirus?" with response categories* (i) the radio, (ii) the TV, (iii) the What's App messenger, (iv) Community Health Workers (CHWs) and (v) religious leaders. Desire for additional information on COVID-19 was measured using the question: *"What more would you like to know about COVID-19?"* with response categories (i) Symptoms of the new Coronavirus, (ii) How it is transmitted, (iii) what to do if you have symptoms; Perceived risks from COVID-19 (i) *"How dangerous do you find the new Coronavirus?"*, (ii) *"Do you think you are likely to become sick with the new Coronavirus"*, and (iii) "*Do you think any of your family members are at risk of becoming sick from the new Coronavirus?"*. Responses to the first question were categorised as very dangerous, dangerous or "not at all". The latter two were combined due to small numbers reporting 'not at all'. Response

For peer review only - http://bmjopen.bmj.com/site/about/guidelines.xhtml

to the other two questions consisted of yes, no and do not know. Information on gender, survey location, and age group were also collected.

Statistical analyses: All statistical analyses used the Stata V.16 software [21] and the statistical significance level set at 5%. The association between the exposures (wealth quintiles and educational level) and the outcomes of interest (knowing that COVID-19 can be prevented by specific behaviours) was explored using Poisson regression [22]. Poisson regression was preferred over logistic regression to allow relative risks to be presented, rather than odds ratios which are often misrepresented. All regression analyses applied survey weights to account for the higher proportion of women included in the survey (57% vs. 43% for males) and the higher proportion of survey participants with secondary education and above (28.5% vs. 23.7% for those with no education). The multivariable regression analysis adjusted for wealth quintiles, educational level, gender, survey location, age group, trusted sources of information about COVID-19, desired additional information on COVID-19 and perception of risks.

Qualitative interviews

Sampling procedure: Purposive sampling was used to identify and select respondents with professional or personal experience of COVID-19. Semi-structured, in-depth telephone interviews were done with policy makers, healthcare workers, truckers, traders and COVID-19 survivors and carers to assess their experiences and needs in relation to the pandemic and mitigation measures specific to border counties' populations. Interviewees were mapped using an initial stakeholders list which included the counties' leadership, health management teams, and the COVID-19 response teams, to which further additions were made through snowballing. Interviews were completed between September and October 2020.

Data collection and analysis: Key informant interviews were carried out to explore understanding and experiences of the pandemic. The interviews were conducted in either Kiswahili or English based on preference of the interviewees, time range of 30-60 minutes per interview. Questions related to the following topics were explored: community response to, and experience of the pandemic; health systems' preparedness and response to the pandemic; and border-specific issues related to the pandemic (only data pertaining to prevention behaviors are presented in this paper). The recorded interviews were transcribed, translated, and checked for quality by the Kenyan research team, and a UK-based co-author (CC) who is fluent in Swahili. The transcripts were then analysed using the framework method (Gale et al. (2013) [23]: open-coding of the data was undertaken in pairs or small teams (with at least one Kenyan, and one UK-based individual) so that sociocultural differences in interpretations of the data could be identified and engaged with. The team then met altogether to develop an analytical framework with key categories (i.e. groupings of codes that are clearly defined). The

For peer review only - http://bmjopen.bmj.com/site/about/guidelines.xhtml

analytical framework was then applied to all of the transcripts. Illustrative quotes were charted into a framework matrix and the data was interpreted and organized into themes.

Ethical considerations: The study protocol was approved by the KEMRI Scientific and Ethics Review Committee (Ref. No. KEMRI/SERU/CPHR/4021), and the National Commission for Science, Technology and Innovation (Ref. No. 864492). Informed consent was sought and obtained from participants prior to their participation.

Patient and Public Involvement: The research question was arrived at upon interrogating the existing covid-19 response measures and realising a potential gap in addressing the needs of border communities. The County administration assessed the relevance of the proposed research and gave permission for community participation in the study. Community health workers and village elders assisted in sensitising and mobilising community participation, participated in mapping and selection of households, and sampling of study participants. The study findings were discussed in stakeholder forums with representation from the counties, community-based organisations and members of health committees of the local legislature. Recommendation items actionable at county level were taken up by respective county teams for implementation.

RESULTS

Knowledge of key COVID-19 prevention behaviours in Busia and Mandera

We assessed levels of knowledge of key COVID-19 prevention behaviours, and whether these varied by socioeconomic factors. Table 1 shows the sample profiles for the 582 adults (Busia: 50.5%; Mandera 49.5%) who participated in the e-survey.

Table 1: Sample profiles in Busia and Mandera Counties

	**Busia (N=294)**	**Mandera (N=288)**	**All (N=582)**
	**Proportion (95% CI)**	**Proportion (95% CI)**	**Proportion (95% CI)**
**Age group**			
15-24 (Early working age)	8.6 (5.6 to 12.9) §	1.4 (0.6 to 3.5)	5.0 (3.4 to 7.3)
25-54 (Prime working age)	6.8 (4.3 to 10.6)	7.2 (4.7 to 10.8)	7.0 (5.1 to 9.5)
55-64 (Mature working age)	59.9 (52.8 to 66.7)	52.1 (45 to 59.1)	56.0 (51 to 60.9)
65 plus (Elderly)	24.7 (18.5 to 32) §	39.3 (32.3 to 46.8)	32 (27.2 to 37.3)
**Education level**			
Secondary and above	40.9 (34.4 to 47.6) §	15.1 (11.4 to 19.9)	28.0 (24.0 to 32.3)
Primary 5-8 yrs	28.6 (23.6 to 34.1) §	3.4 (2 to 5.8)	16.0 (13.4 to 19.0)
Primary 1-4years/Duksi/Madrasa	25.8 (19.1 to 34) §	58.2 (51.6 to 64.5)	42.0 (36.9 to 47.3)
None	4.7 (3.0 to 7.4) §	23.3 (18.9 to 28.3)	14.0 (11.6 to 16.8)
**Wealth quintiles**			
Highest quintile	16.9 (2.5 to 12.6) §	23.2 (17.8 to 29.7)	20.1 (16.5 to 24.2)
2nd	21.5 (2.7 to 16.7) §	12.7 (9.1 to 17.4)	17.1 (14.0 to 20.7)
3rd	29.7 (3.4 to 23.4) §	16.3 (11.6 to 22.4)	23.0 (18.9 to 27.7)

	Busia (N=294)	Mandera (N=288)	All (N=582)
	**Proportion (95% CI)**	**Proportion (95% CI)**	**Proportion (95% CI)**
4th	17.8 (2.7 to 13.1) §	18.4 (14 to 23.9)	18.1 (14.8 to 22.0)
Lowest	14 (2.4 to 10) §	29.4 (23.1 to 36.6)	21.7 (17.8 to 26.2)
**Preventative behaviours**			
Wash hands regularly with soap and water	95.9 (92.3 to 97.9) §	77 (70.4 to 82.5)	86.5 (82.6 to 89.6)
Cover mouth and nose when coughing or sneezing	64.3 (57.3 to 70.7) §	48.3 (41.3 to 55.3)	56.3 (51.4 to 61.1)
Hand sanitiser use	84.4 (78.7 to 88.9) §	65.2 (58.3 to 71.6)	74.8 (70.3 to 78.9)
Standing 2 meters away from other people	52.2 (45.3 to 59.1) §	27.9 (22.2 to 34.6)	40.1 (35.4 to 45.0)
Wear a face mask	70.9 (64.2 to 76.9) §	55.3 (48.2 to 62.3)	63.1 (58.2 to 67.9)
**Trusted source of information on COVID-19**			
Radio	92.8 (88.6 to 95.5) §	54.8 (47.8 to 61.7)	73.8 (69.4 to 77.8)
TV	51.6 (44.7 to 58.4) §	11.8 (7.9 to 17.4)	31.7 (27.3 to 36.4)
What's up	25.2 (19.8 to 31.5)	19.1 (14.2 to 25.3)	22.2 (18.4 to 26.5)
Community Health Workers	56.4 (49.4 to 63.1) §	43.7 (37.0 to 50.7)	50 (45.1 to 55)
Religious leaders	33.3 (27.3 to 39.9) §	46.2 (39.3 to 53.3)	39.7 (35 to 44.6)
**Kind of information received**			
Symptoms of COVID-19	82.3 (76.5 to 86.9) §	60.3 (53.2 to 66.9)	71.3 (66.6 to 75.5)
How SARS CoV2 is transmitted	74 (67.5 to 79.5) §	40.9 (34.2 to 47.9)	57.4 (52.5 to 62.2)
What to do if you have symptoms	65.7 (58.9 to 72) §	28.7 (22.7 to 35.6)	47.2 (42.3 to 52.2)
**Perception of risk**			
**How dangerous COVID-19 is?**			
Very dangerous	89.3 (84.4 to 92.8) §	73.2 (66.7 to 78.9)	81.3 (77.2 to 84.8)
Less dangerous/Not dangerous	10.7 (7.2 to 15.6) §	26.8 (21.1 to 33.3)	18.7 (15.2 to 22.8)
**Do you think any of your family member are at higher risk of becoming sick?**			
Yes	53.3 (46.4 to 60)§	20.4 (15.6 to 26.2)	36.8 (32.3 to 41.7)
No	13.8 (9.8 to 19.2)	13.8 (9.9 to 18.9)	13.8 (10.9 to 17.4)
Don't know	32.9 (26.8 to 39.6)§	65.8 (59.1 to 71.9)	49.4 (44.4 to 54.3)
**Do you think you are likely to become sick with COVID-19?**			
Yes	56.1 (49.2 to 62.8)§	16.3 (12.2 to 21.4)	36.2 (31.6 to 41)
No	14 (9.9 to 19.3)	16.8 (12.3 to 22.6)	15.4 (12.2 to 19.2)
Don't know	29.9 (24.1 to 36.6)§	66.9 (60.3 to 72.9)	48.4 (43.5 to 53.4)
COVID-19 symptoms			
Fever	12.9 (9 to 18.1)	20.2 (15.6 to 25.8)	16.5 (13.4 to 20.2)
Cough	12.5 (8.6 to 17.7)	21.1 (16.3 to 26.9)	16.8 (13.6 to 20.6)
Shortness of breath	7.7 (4.6 to 12.5)	9.9 (6.6 to 14.6)	8.8 (6.4 to 12)

For peer review only - http://bmjopen.bmj.com/site/about/guidelines.xhtml

For peer review only

	Busia (N=294)	Mandera (N=288)	All (N=582)
	**Proportion (95% CI)**	**Proportion (95% CI)**	**Proportion (95% CI)**
Sore throat	7.1 (4.3 to 11.7)	6.4 (3.8 to 10.3)	6.7 (4.7 to 9.6)
Fatigue	5.2 (2.8 to 9.5)	4.5 (2.5 to 9.1)	4.9 (3.1 to 7.4)
Loss of taste or smell	5.9 (3.2 to 10.7)	2.1 (0.9 to 5)	4 (2.4 to 6.6)

§ 95% CIs do not overlap

Majority (95%) of the participants were 25 years and above, 14% lacked formal education. Socio-economic disadvantage was high in both locations, with just over a quarter in Busia and over half of those in Mandera reporting having received only Primary 1-4 years, Duksi or Madrasa education. Mandera was consistently more disadvantaged than Busia across all measures. Overall, 17% reported a fever and cough, while 4% reported loss of smell/taste two weeks before the survey.

In both areas, the most known common prevention behaviours identified were washing hands regularly 86.5% (Busia 95.9%, 95% CI 92.3-97.9; Mandera 77%, 70.4-82.5) and use of a hand sanitiser 74.8% (Busia 84.4%, CI 78.7-88.9; Mandera 65.2%, CI 58.3-71.6) and the least commonly known was standing two metres away from others 40.1% (Busia 52%, 45.3-59.1; Mandera 28%, 22.2-34.6). Significantly lower proportions in Mandera than in Busia reported knowledge of prevention behaviours, having had secondary education, being in the most disadvantaged 4th and 5th wealth quintiles, having trust in COVID-19 related information from various sources including the radio and TV, and having received COVID-19 related knowledge on symptoms, transmission and what to do if they have symptoms. There were some notable differences for trusted sources of COVID-19 information. In Busia, 93% cited the radio as the most trusted source, whereas in Mandera the radio (55%), community health workers (44%) and religious leaders (46%) shared the top ranking for trusted source. Table 2 shows the influence of adjusting for SEC, trusted sources of information, additional information and perception of risk on the Mandera-Busia differences in knowledge of prevention behaviours.

Table 2: The influence of adjusting for socio-economic circumstances, trusted sources of COVID-19 information, and additional information and perception of risk on the Mandera-Busia differences in knowledge of prevention behaviours. Risk Ratio (95% confidence interval), Busia is the reference category.

	Mandera-Busia difference in prevention behaviour, adjusted for gender and age	+ education level and wealth quintiles	+ trusted sources of information about COVID-19 knowledge	+desired additional information on COVID-19, and perception of risk
Wash hands regularly with soap and water	0.80 (0.74 to 0.87)***	0.81 (0.74 to 0.89)***	0.81 (0.73 to 0.90)***	0.86 (0.78 to 0.96)**

For peer review only - http://bmjopen.bmj.com/site/about/guidelines.xhtml

Cover mouth and nose when coughing or sneezing	0.72 (0.61 to 0.87)***	0.81 (0.64 to 1.03)	0.87 (0.67 to 1.14)	0.99 (0.76 to 1.28)
Hand sanitiser use	0.77 (0.68 to 0.86)***	0.80 (0.69 to 0.92)**	0.88 (0.75 to 1.02)	0.99 (0.85 to 1.16)
Standing 2 meters away from other people	0.52 (0.40 to 0.68)***	0.61 (0.44 to 0.85)**	0.58 (0.40 to 0.84)**	0.71 (0.50 to 1.01)$^{+}$
Wear a face mask	0.77 (0.66 to 0.90)**	0.97 (0.79 to 1.20)	0.93 (0.73 to 1.17)	1.06 (0.83 to 1.36)

$^{+}$ p <0.1 and >0.05, * p<0.05, ** p<0.01, *** p<0.001

The lower knowledge of washing hands in Mandera compared with Busia remained after fully adjusting, with hardly any shift in the risk ratios. The differences between Mandera and Busia in knowledge about covering the mouth when coughing/sneezing and wearing a mask were no longer significant on adjustment for SEC, for using a sanitiser on adjustment for trusted sources of COVID information, and for keeping a 2 metres distance from others on adjustment for all confounders.

Based on the pooled Busia and Mandera sample and after adjusting for confounders including location, the knowledge of prevention behaviours did not vary by education (Supplementary Figure 1). In contrast, regardless of location those in the most disadvantaged 5th wealth quintile remained less likely to report knowledge of three of the five prevention behaviours – washing hands regularly (- 20%, 95% CI -7 to -31%), covering mouth and nose when sneezing or coughing (-34%, -10 to -53%) and standing two metres away from others (-39%, -4 to -61%).

Significant interactions were observed between quintiles of wealth and location for knowing that covering the mouth when coughing or sneezing (F=35.23, p<0.001) and keeping 2 metres distance (F=13.26, p=0.009) could prevent COVID-19. The most socio-economically disadvantaged participants in Mandera were 62% (95% CI 33%-79%) less likely to know that COVID-19 could be prevented by covering their mouth while coughing/sneezing, and 61% (26% -89%) less likely to know that COVID-19 could be prevented by keeping 2 metres distant (See Supplementary Figure 2). No significant differences in the knowledge of this behaviours were observed between the least and the most socioeconomically disadvantaged in Busia.

Qualitative insights into factors impacting on engagement with prevention and control measures

A total of 73 qualitative interviews (Busia: n= 55; Mandera: n= 18) were carried out with policy actors (n=8), healthcare workers (n=34), truckers and traders (n=7), COVID-19 survivors and carers (n=8) and other community members including religious leaders, village elders, persons living with disability, and commercial sex workers (n=16) in the two counties. Age of interviewees ranged between 23-74 years.

For peer review only - http://bmjopen.bmj.com/site/about/guidelines.xhtml

The qualitative interviews provided insights into contextual factors which impacted on engagement with prevention and control measures. Table 3 provides a summary, including quotes from the qualitative interviews, to illustrate factors impacting on engagement with prevention measures in the two border regions. Lack of trust in the national government and political leaders was noted as a key barrier to engagement with prevention measures in the two border regions. Participants reported that this lack of trust mainly stemmed from alleged misappropriations of funds and politicians not adhering to COVID-19 restrictions.

Other reported impacts included socioeconomic factors such as the loss of livelihoods (especially in the informal sector), and increased cost of living due to rising food prices occasioned by disruptions in local trade and food imports, which impacted COVID-19 prevention measures. Psychosocial factors such as the centrality of spiritual support and family social connections in communities' cultures and way of life conflicted with preventive measures.

Additionally, there was reported to be inadequate attention to providing targeted, context specific, accessible risk communication and public health advice which impacted on equitable understanding and adherence. Challenges included use of technical global pandemic language and procedures that were not translatable to the local setting; lack of community engagement in tailoring communication messages; lack of targeted messaging for the pastoralist community in Mandera; language barriers with French-speaking truckers from Congo and Rwanda; and misinformation within the community regarding COVID-19 symptoms and transmission. Some initiatives by the two county governments were reported to facilitate engagement with prevention, including informal provisions of hand washing stations at the border posts, markets, shops, and religious institutions.

Challenges specific to border areas included, lack of preparedness for truck border crossings which led to pooling of truckers at the border thus risking community transmission. Cross-border collaboration in enforcing the preventive measures was enhanced through leveraging an already existing infectious disease surveillance network in Mandera but challenged by stigmatization of truckers in Busia.

For peer review only - http://bmjopen.bmj.com/site/about/guidelines.xhtml

Table 3: Qualitative Insights on impacts on adherence to prevention measures

* (B/M) indicates location (Busia/Mandera), (M/F) indicates gender (male/female).

THEMES	CATEGORIES	ILLUSTRATIVE QUOTES
**Distrust of national government and politicians**	Politicians not adhering to COVID restrictions	BM-Imam-118: "They look at the politicians and how they are conducting themselves. Those breaking the rules do so because those that are supposed to encourage them are not adhering to the rules"<br>BM-Comm.HealthVolunteer-018: "people see the leaders walking without wearing masks [… so] they believe there is no corona"<br>MM-Trader-074 "we see politicians doing things without social distance… it only means there is no COVID"
	Media coverage of misappropriation of COVID funds	BM-Comm.HealthVolunteer-003: "having heard that there is a lot of money that was squandered through Kenya Medical Supplies Authority… majority of them are not doing the right thing [i.e. taking preventative measures]"<br>BM-PublicHealthOfficer-052: "people started relaxing in adhering after this scandal was unearthed whereby they say that billions of money was stolen"
**Health messaging not adequately contextualised to local communities**	Insufficient attention to impact of isolation on livelihoods	BM-COVIDSurvivor-111: "people fear to go and test, because they know you will just dump somebody there [in an isolation centre] for ten days […] you don't know the person, the way they eat, survive, he is just there like that in the name of quarantine, isolation, all those things"
	Inadequate community engagement and tailored public health messaging	BM-Policyactor-087: "we need to have more community engagement, with interpersonal communication kind of approach, so that you do more than just general messages, you are doing specific messages to specific groups of people, truck drivers, sex workers, maybe clearing agents and so on"<br>BM-Policyactor-113: "Communication on protection was not well done. We needed more of community engagement and good communication to ensure that all issues are clarified through question and answers"<br>BM-Policyactor-096: "remember the kind of information which was being given out initially [… led] by the Ministry of Health was more of .. well it was a caution but it really sounded to the public like this thing is going to wipe away so many people […] the words were really confusing, quarantine, isolation and so on"
	Circulation of misinformation in the community	- 'COVID is just like malaria, it should not be feared' (BM-Farmer-085; BF-Comm.HealthVolunteer-004).<br>'COVID only affects people in big cities and truckdrivers' / 'people do not know people who have had COVID - seeing is believing' (BF-Policyactor-025; BM-CommercialSexWorker-068; MM-Policyactor-007).
	Difficulties engaging with pastoralist communities (in Mandera)	MM-Policyactor-112: "the pastoralist nature of our society, you know people moving from here to the other side [of the border], uncontrollable has also been a serious issue [… they get health information from listening to] vernacular radio stations […] [but] compliance is another thing"<br>MM-Policyactor-007: "we had cases that were imported from Somalia by people who went to sell their livestock [and crossed the border unchecked]"
**Psychosocial and socioeconomic impacts on**	Desire for spiritual and social connection	BM-Policyactor-087: "people are social they want to be together but now they have been told to keep a distance… And when churches were closed, this was very strange… you will find it is affecting people spiritually"<br>BM-Policyactor-087: ""where people used to greet you by shaking hands, and now people are supposed to learn a new mode of greeting which is not our culture and to kick Kenyan culture is not so easy"

For peer review only - http://bmjopen.bmj.com/site/about/guidelines.xhtml

**preventive measures**		MF-COVIDSurvivor-098: "To protect yourself from corona, you are supposed to wear masks, don't greet people and also restrain from hugging people but sometimes you forget and greet people using your hands, you hug people because you are used to this"
	Socioeconomic barriers	BM-PLWDisability-117: "where will we disabled people get masks from? […] We have to work hard and buy for ourselves from our own pockets, buy for the children"<br>MM-Healthworker-037: "there's nobody who can provide them with masks, they were buying at […] twenty shillings or fifty shillings, and so it was very expensive?'"<br>MM-Policyactor-112: "There are these small [informal] businesses [e.g. tea/coffee stands], and… when we want to enforce those [social distancing] regulations it becomes very difficult because this is somebody's daily bread".
	Religious and social spaces supporting adherence	MM-Trader-074: "Even at the shop I have sanitiser, running water with soap, so people when they come to the shop they sanitize".<br>MF-COVIDSurvivor-098: "We have [hand]washing stations in hospitals, madrasa, at the mosque"<br>MM-PublicHealthOfficer-039 "we had a meeting with all the religious leaders… [to discuss how some preventive] measures… So after sensitizing, at least people have started accepting [preventive measures], because it was a big challenge for people to accept social distance in the place of worship"
**Issues specific to border areas**	Lack of preparedness for truck border crossings	BM-Policyactor-096: "initially we had lots of challenges... the trucks piled up... [resulting] in drivers not being able to cross over to Uganda on time, they ended up mingling with the rest of the population and so we knew it was just a matter of time before we got the first community cases"<br>BM-PublicHealthOfficer-052: ""you find this community transmission tends to be growing [because truckdrivers must wait for 2-3 days for the COVID test results]… we feel that there is a need for testing to be done across the entire [border] township […] but we lack the resources for doing this"
	Health prevention information not translated into all needed languages	BM-Policyactor-087: "people from Congo, from Rwanda, they speak French and […] most of our communications are in Kiswahili and English and therefore—like now we don't have any material for French here at the border and that is a huge challenge"
	Lack of consistency in cross-border communication	MM-Policyactor-007: "previous cooperation about polio issues […] can also help in responding to this pandemic"<br>BM-PublicHealthOfficer-052: "It is compulsory in Kenya to put on masks…these drivers when in Uganda they are not allowed to mingle with people, they think that anybody coming from Kenya is having Corona [because of the mask wearing]"<br>BM-Policyactor-113: "Kenya and Uganda might not have been reading from the same script. The countries should have the same lab where they collect the samples and the same approach to the communities. I believe they have community health issues at the border points [like us]. In times of a pandemic, no one is to blame. We have our own challenges but those that spread across East Africa would be dealt better with collaboration"
	Disruptions to cross-border trade and food security	BM-TraditionalHealer-072: "you can't cross the border to go to the Ugandan side [of the forest to collect herbs], if you are found, maybe you passed sideways, you get arrested. We had to stop working"<br>BM-Comm.HealthVolunteer-003: "there is food insecurity, people used to do farming freely, do business, they are unable to cross the border"

For peer review only - http://bmjopen.bmj.com/site/about/guidelines.xhtml

For peer review only

DISCUSSION

We assessed the levels of knowledge of key COVID-19 prevention behaviours in the two border counties. The best known preventive behaviour was washing of hands regularly and the least known was standing two metres apart, with Mandera significantly disadvantaged. We observed disparate knowledge of COVID-19 prevention measures based on socioeconomic status. Those with less education and those in lower socioeconomic groups were less likely to adhere to COVID-19 prevention measures. Key factors impacting on engagement with COVID-19 prevention measures included lack of trust in the leadership, deficits in public health communication, livelihood insecurity, prioritization of social support over quarantine/social distancing, and border-specific complexities such as language barriers and congregating of truckers due to lack of preparedness for truck border crossings.

Influence of socioeconomic inequalities on knowledge of COVID-19 prevention behaviours

Our study findings are consistent with previous studies in Kenya and other parts of Africa that observed variations in knowledge and practice of COVID-19 prevention measures based on employment status, access to information, education level, geographical location and gender [24-26], further demonstrating the influence of socioeconomic inequalities on knowledge, awareness and reach of health information.

The high awareness of COVID-19 reported in this study can largely be attributed to adoption of a whole of government and multi-agency approach (policy and technical) in response to the COVID-19 threat, accompanied by widespread media campaigns and news reports. A long history and experience with infectious disease response may have made messaging on hand hygiene and cough/sneeze etiquette more acceptable and readily implementable in Kenya. This however was not achieved with wearing masks and maintaining social distance as they were considered novel interventions. Lags in supply and affordability of masks and sanitising agents, and difficulties in sustaining lockdowns and movement restriction as reported in this study and elsewhere [14], contributed to failure in observing regular use of masks and social distancing. Low adherence to mask wearing and social distancing at a time of widespread consensus on COVID-19 being an airborne disease coupled with the low vaccination rate [15], call for innovative ways to engage communities in developing contextually relevant public health measures, particularly when introducing novel interventions, cognisant of the influence of local culture and the impact of the socio-economic environment on the capacity for adherence. Likewise, emphasis on building community knowledge and not just providing top-down instructions, engaging communities in the adaptation processes as previously shown in Bangladesh [27], has the potential to improve adherence and ownership through better understanding. Further, the reported need for additional information

For peer review only - http://bmjopen.bmj.com/site/about/guidelines.xhtml

on the disease indicates a lag in adjusting the communication messaging to the evolution of the pandemic, a gap that requires addressing.

Public health information

Our study found that community engagement with preventive measures improved with access to appropriate messages from preferred channels of communication. Socio-economic inequalities observed were slightly mitigated by trust placed in the source of information (religious leaders, WhatsApp, radio, and community health workers), signalling the opportunity to capitalize on the trust hierarchy to facilitate adherence to preventive behaviours. The pandemic communication strategy needs to be tailored to the local context and the dynamics of information flow to address gaps in information penetration and engagement. Key in this process is the translation of the standardised pandemic language and procedures to local contexts through participatory processes. In addition, the reported community's loss of trust in the genuineness of the government efforts warrants a concerted effort to win back the public to comply with COVID-19 prevention, possibly through dialogue. The pastoralist communities require special attention as a key population due to their nomadic way of life that can marginalize them in respect of public health awareness and prevention measures and could increase the risk of cross-border transmission and potential recurrent outbreaks [14]. The risk is exacerbated by limited access to water and sanitation. There is need therefore for concerted effort in improving health literacy through tailored education campaigns and messaging in the border regions, and especially among the pastoralists, supported by structural interventions to enable disadvantaged and marginalised communities adhere to prevention measures.

An added dimension to risk communication is the stability and strength of mobile network coverage, which tends to be poorer in rural areas. Representativeness in sampling of participants in both counties could reflect the relative ease of access to local radio when compared to television and internet-based social media, in contrast to other work done in urban settings in Kenya [24,25].

Border dynamics

Border health security is key to safeguarding the EAC's economic and social security. Studies show that implementation of COVID-19 interventions are likely problematic for populations with weak health systems, high density housing, porous borders and insecurity [28]. The disruptions to trade and livelihoods have had profound effects on border communities [29]. Concerns over financial stability (loss of income and food security) have persisted over time. There is a need to economically empower and build resilience among border communities to withstand the current and similar shocks in future. The border counties had to cater for a bigger population than their official figures, and as such, budgetary allocation to these counties should recognize this

For peer review only - http://bmjopen.bmj.com/site/about/guidelines.xhtml

reality. The mandatory testing of truck drivers caused a major backlog in Busia which lies on the Northern Transport Corridor. As gathering points for large numbers of people, border crossings are inherently a risk point for transmission of SARS Cov2. This calls for better organization in designating and preparing holding areas in border regions. This could have potentially protected the local communities in the initial phase of COVID-19 and later with established community spread, the uninfected drivers.

In addition to the harmonised testing protocol of truckers by Kenya and her neighbours, there is also a need to strengthen cross-border surveillance networks to support contact tracing to increase the likelihood of containment of outbreaks and reduce the risk posed by cross-border health threats [30]. Adherence to prevention measures and movement restrictions at the borders were reportedly undermined by porous borders and strong social ties between the cross-border communities. The COVID-19 preventive measures and mitigation measures should thus be accompanied by intensive community engagement to minimise a sense of alienation among these communities. The use of informal crossings should be mitigated by developing criteria accessible to all that recognizes the inherent economic and social challenges in the region.

CONCLUSION

This research highlights the importance of pandemic response and communication in engagement with COVID-19 preventive measures in border settings. As in other contexts, socioeconomic inequalities were reflected in knowledge of and adherence to COVID-19 prevention measures, with the less educated and those in lower socioeconomic groups at greatest risk. The influence of trusted sources of information in mitigating such inequalities demonstrate the need for tailored public health communication strategies that are cognisant and responsive to local context and patterns of information flow. Further effort needs to be made in risk communication by modifying health messaging in tandem with evolution of the pandemic and incorporating community communication needs. Improving health literacy may support better health outcomes for the population.

Livelihood insecurity directly prevents the most disadvantaged from adhering to prevention measures. Economic empowerment and building resilience against future shocks is thus an important investment the country needs going forward. Coordinating response measures across border points is crucial in winning the community's trust and maintaining essential economic and social activities.

CONTRIBUTORS: KL, OJ, KA, MJ, CC, AI, LV, RU, DP, CK, ME and HS participated in conceiving the study, implementation and drafting of the manuscript. All authors contributed to the interpretation and subsequent revision of the paper. All authors approved the final version of the manuscript.

COMPETING INTERESTS: None

For peer review only - http://bmjopen.bmj.com/site/about/guidelines.xhtml

FUNDING: This work was supported by the Kenya Medical Research Institute (KEMRI) Internal Research Grant number KEMRI/COV/SPEC/002.

DATA SHARING AGREEMENT: No additional data available

ACKNOWLEDGEMENTS: We are grateful to all the study participants from Busia and Mandera Counties who took time to participate in this study. We thank the Director KEMRI, the National Commission for Science, Technology and Innovation, and the County Governments of Busia and Mandera for granting permission and providing an enabling environment to undertake this study. Many thanks to our research collaborators at Kings College London for their technical guidance and support, and the KEMRI County Cluster Coordinators Mr. Surrow Adow and Ms. Olipher Makwaga for their support. We thank our research assistants Schiller Mbuka, Rodgers Ochieng, Melvine Obuya, Esther Andia, Miriam Bosire, Doreen Mitaru and Priscilla Maiga for their tireless efforts in bringing the study to fruition.

REFERENCES

1. World Health Organization. WHO Coronavirus (COVID-19) Dashboard | WHO Coronavirus (COVID-19) Dashboard With Vaccination Data. https://covid19.who.int/region/afro/country/ke Accessed 20th January 2022.
2. Pinchoff J, Austrian K, Rajshekhar N, et al. Gendered economic, social and health effects of the COVID-19 pandemic and mitigation policies in Kenya: evidence from a prospective cohort survey in Nairobi informal settlements. *BMJ Open* 2021;11:e042749.
3. World Bank poverty and equity data http://povertydata.worldbank.org/poverty/country/KEN Accessed 28th May 2020.
4. The COVID 19 global pandemic: Impact to the economy and policy options, Parliamentary Budget Office (PBO), Special Bulletin No. 1/2020, Government of Kenya (http://parliament.go.ke/sites/default/files/202004/Special%20Bulletin%20Covid%2019%202.4.2020%20final.pdf)
5. The Seventh Presidential Address on the Coronavirus Pandemic: The 8-Point Economic Stimulus Programme. Government of Kenya (https://www.president.go.ke/2020/05/23/the-seventh-presidential-address-on-the-coronavirus-pandemic-the-8-point-economic-stimulus-programme-saturday-23rd-may-2020/)
6. World Bank. 2021. Socioeconomic Impacts of COVID-19 in Kenya on Households: Rapid Response Phone Survey, Round 1. (https://openknowledge.worldbank.org/handle/10986/35173 License: CC BY 3.0 IGO)
7. Shadmi E, Chen Y, Dourado I, et al. Health equity and COVID-19: global perspectives. *International Journal for Equity in Health* 2020; 19:10

For peer review only - http://bmjopen.bmj.com/site/about/guidelines.xhtml

8. Quaife M, van Zandvoort K, Gimma A, et al. The impact of COVID-19 control measures on social contacts and transmission in Kenyan informal settlements. *BMC Medicine* 2020; 18:316.
9. Ope, M, Sonoiya S, Kariuki J, et al. Regional Initiatives in Support of Surveillance in East Africa: The East Africa Integrated Disease Surveillance Network (EAIDSNet) Experience. *Emerging Health Threats Journal* 2013; *6*.
10. Okongo, O. Malaria pandemic , leading cause of deaths in Busia County. *Talk Africa* 2017. https://www.talkafrica.co.ke/reports-indicate-malaria-cases-cause-deaths-busia-county/
11. Nnnennaya A, & Hagembe J. Increasing Access to Healthcare Services for Cross-Border and Mobile Communities in East Africa – Global Health Innovation Center, 2019. (https://dukeghic.org/2019/07/19/increasing-access-to-healthcare-services-for-cross-border-and-mobile-communities-in-east-africa/)
12. World Health Organisation. Horn of Africa countries in a communique to speed up polio eradication. Regional Office for Africa. 2018. https://www.afro.who.int/pt/node/10538
13. East African Community. Mapping of Health Services along major transport corridors in East Africa. East African Community Report, 2015.
14. African Union. The COVID-19 Pandemic in the IGAD region: Impact on the Health and Economy Vol 1. Horn Economic and Social Policy Institute (HESPI): Pillar 2 An assessment of the health and economic impact on the HoA countries, 2020. (https://media.africaportal.org/documents/The-COVID-19-Pandemic-in-the-IGAD-region_VOL-I.pdf)
15. Kenya Ministry of Health. COVID-19 outbreak in Kenya. Daily Situation Report 673. 19th January 2022.
16. Kenya Ministry of Health. Kenya Harmonized Health Facility Assessment Report (KHFA) 2018/2019 Report.
17. Kenya National Bureau of Statistics. Kenya Population and Housing Census. Volume IV: Distribution of Population by Socio-Economic Characteristics 2019 (https://www.knbs.or.ke/?wpdmpro=2019-kenya-population-and-housing-census-volume-iv-distribution-of-population-by-socio-economic-characteristics)
18. McBurney P. On transferring statistical techniques across cultures: The Kish Grid. Current Anthropology 1998; 29:323-325.
19. World Health Organization. Risk Communication and Community Engagement (RCCE) Action Plan Guidance COVID-19 Preparedness and Response, 2020 (https://www.who.int/publications/i/item/risk-communication-and-community-engagement-(rcce)-action-plan-guidance)
20. Harris PA, Taylor R, Thielke R, et al. Research electronic data capture (REDCap)—A metadata-driven methodology and workflow process for providing translational research informatics support. *Journal of Biomedical Informatics* 2009; 42: 377–381.

For peer review only - http://bmjopen.bmj.com/site/about/guidelines.xhtml

21. StataCorp. 2019. Stata Statistical Software: Release 16. College Station, TX: StataCorp LLC.

22. Zou G. A modified Poisson regression approach to prospective studies with binary data. *Am J Epidemiol.* 2004;159(7):702–6.

23. Gale NK, Heath G, Cameron E, et al. Using the framework method for the analysis of qualitative data in multi-disciplinary health research. *BMC Medical Research Methodology* 2013; 13:117

24. Karijo E, Wamugi S, Lemanyishoe S, et al. Knowledge, attitudes, practices, and the effects of COVID-19 among the youth in Kenya. *BMC Public Health* 2021; 21(1):1020.

25. Austrian K, Pinchoff J, Tidwell JB, et al. COVID-19 related knowledge, attitudes, practices and needs of households in informal settlements in Nairobi, Kenya. [Preprint]. *Bull World Health Organ*. E-pub: 6 April 2020. doi: http://dx.doi.org/10.2471/BLT.20.260281

26. Sengeh P, Jalloh MB, Webber N, et al. Community knowledge, perceptions and practices around COVID-19 in Sierra Leone: a nationwide, crosssectional survey. *BMJ Open* 2020;10:e040328.

27. Abaluck J, Kwong LH, Styczynski A, et al. Impact of community masking on COVID-19: A cluster-randomized trial in Bangladesh. *Science* 2022;14;375(6577):eabi9069.

28. Da'ar OB, Haji M & Jradi H. Coronavirus Disease 2019 (COVID-19): Potential implications for weak health systems and conflict zones in the Middle East and North Africa region. *Int J Health Plann Manage* 2020; 35(5):1240-1245.

29. Quaife M, van Zandvoort K, Gimma A, et al. The impact of COVID-19 control measures on social contacts and transmission in Kenyan informal settlements. *BMC Medicine* 2020; 18:316.

30. East African Community. Standard Operating Procedure - Cross-border Surveillance in the EAC Region East African Community (EAC) Secretariat. www.eac.int

For peer review only - http://bmjopen.bmj.com/site/about/guidelines.xhtml

Figure Legends

Figure 1: Map showing the location of Busia and Mandera Counties in Kenya

For peer review only

For peer review only - http://bmjopen.bmj.com/site/about/guidelines.xhtml

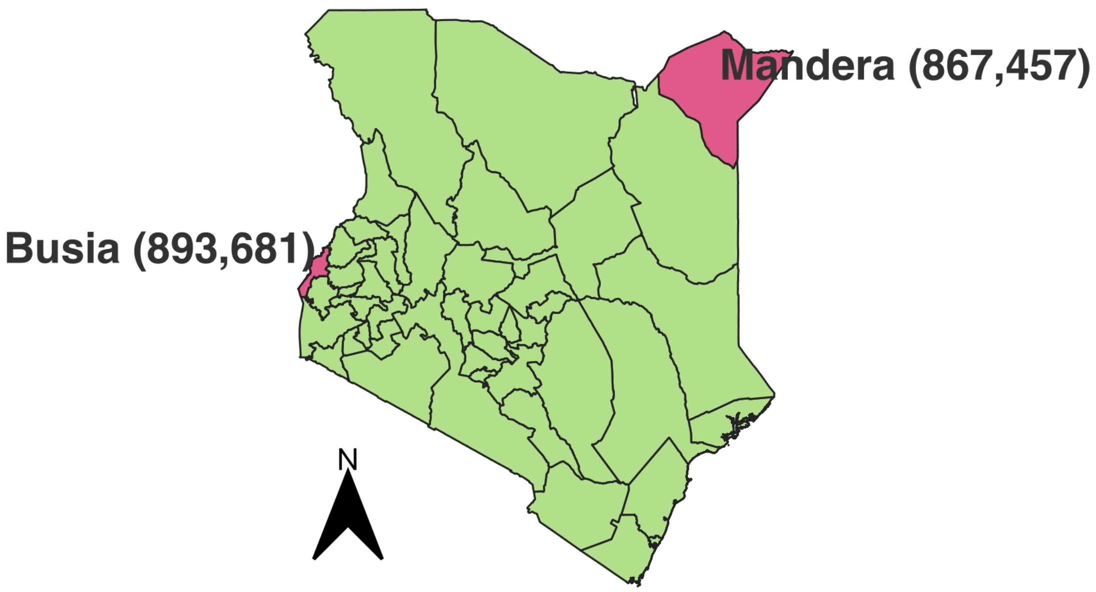
Mandera (867,457)
Busia (893,681)
N

Supplementary Table 1: List of variables used for constructing a wealth index

	Name of Variable	Coding		Factor loadings	
				Factor1	Factor2
1	Ownership of household goods - 1 Clock/Watch	1 = Yes	0 = No		
2	Ownership of household goods - 2 Electricity	1 = Yes	0 = No	.708	
3	Ownership of household goods - 3 Radio	1 = Yes	0 = No		.657
4	Ownership of household goods - 4 Television	1 = Yes	0 = No	.628	.427
5	Ownership of household goods - 5 Mobile telephone	1 = Yes	0 = No	.375	
6	Ownership of household goods - 6 Fixed telephone	1 = Yes	0 = No		
7	Ownership of household goods - 7 Refrigerator	1 = Yes	0 = No		
8	Ownership of household goods - 8 Solar panel	1 = Yes	0 = No		.552
9	Any household member own - 1 Bicycle	1 = Yes	0 = No		.753
10	Any household member own - 2 Motorcyle/Scotter	1 = Yes	0 = No	.427	
11	Any household member own - 3 Animal drawn cart	1 = Yes	0 = No		
12	Any household member own - 4 Car/Truck	1 = Yes	0 = No		
13	Any household member own - 5 Boat with motor	1 = Yes	0 = No		
14	Own house live in	1 = Yes	0 = No		.346
15	Sleeping rooms in the house that live in	1, 2, 3		.640	
16	Finished/cement floor	1 = Yes	0 = No	.681	
	Small coefficients below .30 were suppressed<br>*Eigen values for components one and two were 2.464 & 1.509*				

For peer review only

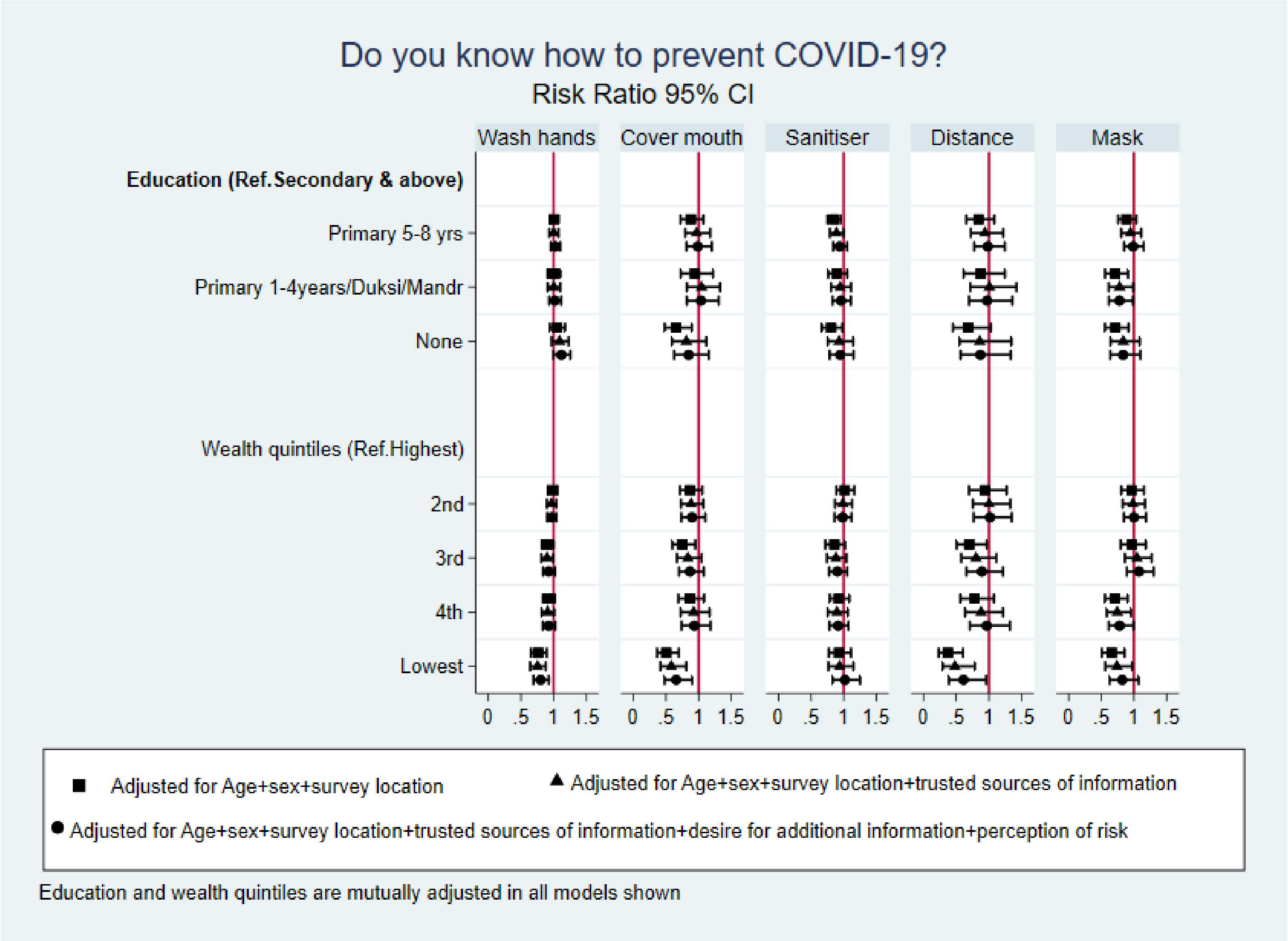

Supplementary Figure 1: Association between knowledge of prevention behaviours and measures of education and wealth. Risk Ratio and 95% confidence intervals.

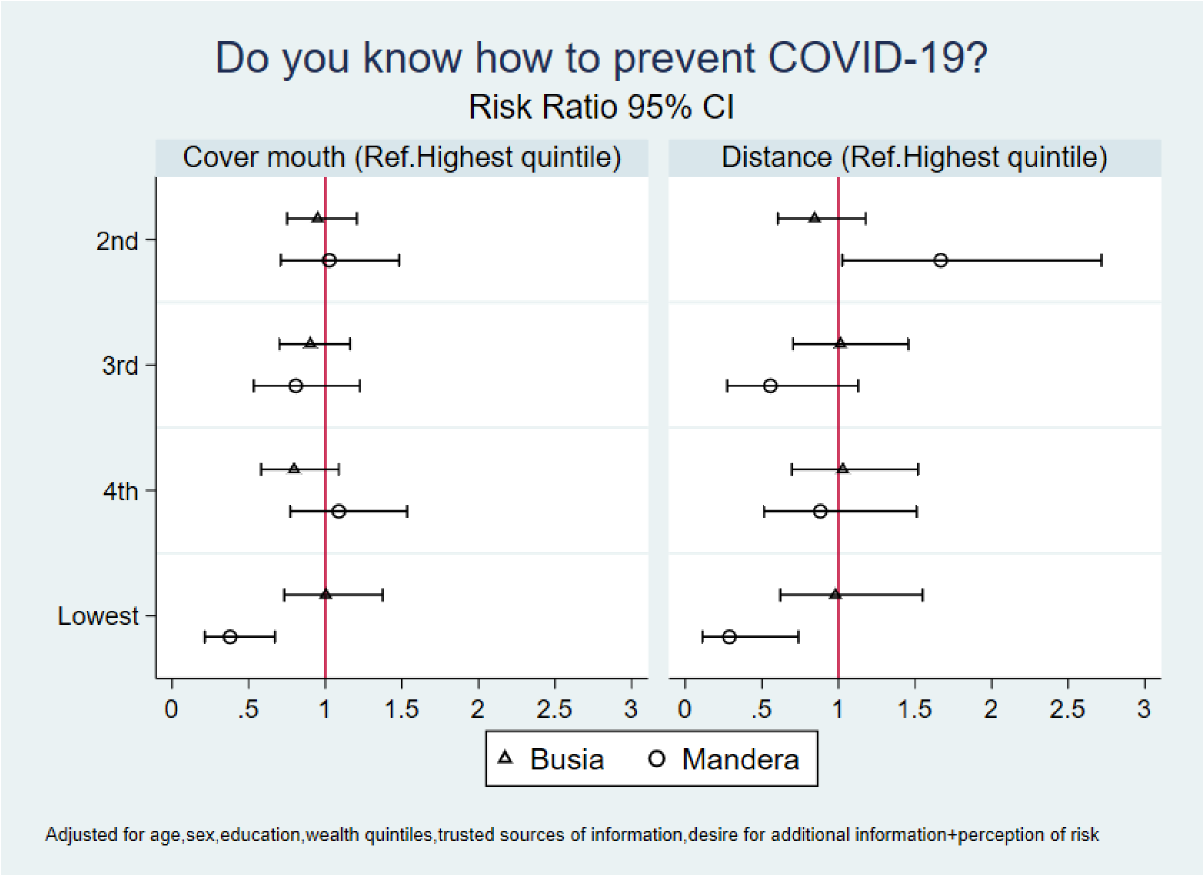

Supplementary Figure 2: Interaction between wealth quintiles and survey area for knowing that covering mouth when coughing/sneezing and keeping 2 metres distance could prevent COVID-19.

For peer review only

STROBE 2007 (v4) checklist of items to be included in reports of observational studies in epidemiology*
Checklist for cohort, case-control, and cross-sectional studies (combined)

Section/Topic	Item #	Recommendation	Reported on page #
**Title and abstract**	1	(*a*) Indicate the study's design with a commonly used term in the title or the abstract	1
		(*b*) Provide in the abstract an informative and balanced summary of what was done and what was found	3
**Introduction**			
Background/rationale	2	Explain the scientific background and rationale for the investigation being reported	4
Objectives	3	State specific objectives, including any pre-specified hypotheses	5
**Methods**			
Study design	4	Present key elements of study design early in the paper	5
Setting	5	Describe the setting, locations, and relevant dates, including periods of recruitment, exposure, follow-up, and data collection	5
Participants	6	(*a*) *Cohort study*—Give the eligibility criteria, and the sources and methods of selection of participants. Describe methods of follow-up<br>*Case-control study*—Give the eligibility criteria, and the sources and methods of case ascertainment and control selection. Give the rationale for the choice of cases and controls<br>*Cross-sectional study*—Give the eligibility criteria, and the sources and methods of selection of participants	5, 7
		(*b*) *Cohort study*—For matched studies, give matching criteria and number of exposed and unexposed<br>*Case-control study*—For matched studies, give matching criteria and the number of controls per case	
Variables	7	Clearly define all outcomes, exposures, predictors, potential confounders, and effect modifiers. Give diagnostic criteria, if applicable	6
Data sources/ measurement	8*	For each variable of interest, give sources of data and details of methods of assessment (measurement). Describe comparability of assessment methods if there is more than one group	6
Bias	9	Describe any efforts to address potential sources of bias	6
Study size	10	Explain how the study size was arrived at	5,7
Quantitative variables	11	Explain how quantitative variables were handled in the analyses. If applicable, describe which groupings were chosen and why	6
Statistical methods	12	(*a*) Describe all statistical methods, including those used to control for confounding	7
		(*b*) Describe any methods used to examine subgroups and interactions	7
		(*c*) Explain how missing data were addressed	7
		(*d*) *Cohort study*—If applicable, explain how loss to follow-up was addressed<br>*Case-control study*—If applicable, explain how matching of cases and controls was addressed	7

For peer review only

		Cross-sectional study—If applicable, describe analytical methods taking account of sampling strategy	
		(*e*) Describe any sensitivity analyses	7
**Results**			
Participants	13*	(a) Report numbers of individuals at each stage of study—eg numbers potentially eligible, examined for eligibility, confirmed eligible, included in the study, completing follow-up, and analysed	8
		(b) Give reasons for non-participation at each stage	
		(c) Consider use of a flow diagram	8
Descriptive data	14*	(a) Give characteristics of study participants (eg demographic, clinical, social) and information on exposures and potential confounders	10
		(b) Indicate number of participants with missing data for each variable of interest	10
		(c) *Cohort study*—Summarise follow-up time (eg, average and total amount)	
Outcome data	15*	*Cohort study*—Report numbers of outcome events or summary measures over time	
		Case-control study—Report numbers in each exposure category, or summary measures of exposure	
		Cross-sectional study—Report numbers of outcome events or summary measures	10
Main results	16	(*a*) Give unadjusted estimates and, if applicable, confounder-adjusted estimates and their precision (eg, 95% confidence interval). Make clear which confounders were adjusted for and why they were included	10
		(*b*) Report category boundaries when continuous variables were categorized	10
		(*c*) If relevant, consider translating estimates of relative risk into absolute risk for a meaningful time period	
Other analyses	17	Report other analyses done—eg analyses of subgroups and interactions, and sensitivity analyses	11-12
**Discussion**			
Key results	18	Summarise key results with reference to study objectives	15
Limitations	19	Discuss limitations of the study, taking into account sources of potential bias or imprecision. Discuss both direction and magnitude of any potential bias	4
Interpretation	20	Give a cautious overall interpretation of results considering objectives, limitations, multiplicity of analyses, results from similar studies, and other relevant evidence	15-17
Generalisability	21	Discuss the generalisability (external validity) of the study results	15-17
**Other information**			
Funding	22	Give the source of funding and the role of the funders for the present study and, if applicable, for the original study on which the present article is based	18

*Give information separately for cases and controls in case-control studies and, if applicable, for exposed and unexposed groups in cohort and cross-sectional studies.

Note: An Explanation and Elaboration article discusses each checklist item and gives methodological background and published examples of transparent reporting. The STROBE checklist is best used in conjunction with this article (freely available on the Web sites of PLoS Medicine at http://www.plosmedicine.org/, Annals of Internal Medicine at http://www.annals.org/, and Epidemiology at http://www.epidem.com/). Information on the STROBE Initiative is available at www.strobe-statement.org.

For peer review only

BMJ Open

Contextually appropriate communication strategies for COVID-19 prevention in Kenya border regions: Evidence from a mixed methods observational study in Busia and Mandera Counties.

Journal:	*BMJ Open*
Manuscript ID	bmjopen-2022-062686.R1
Article Type:	Original research
Date Submitted by the Author:	17-Feb-2023
Complete List of Authors:	Kaduka, Lydia; Kenya Medical Research Institute, Centre for Public Health Research<br>Olale, Joanna; Kenya Medical Research Institute<br>Karamanos, Alexis; King's College London Faculty of Life Sciences and Medicine<br>Mutai, Joseph; Kenya Medical Research Institute<br>Coultas, Clare; King's College London School of Medical Education<br>Ahmed, Ismail; Kenya Medical Research Institute<br>L'Esperance, Veline; King's College London, School of Population Health and Environmental Sciences<br>Read, Ursula; University of Warwick Warwick Medical School<br>Dazzan, Paola; King's College London Institute of Psychiatry Psychology and Neuroscience<br>Cruickshank, John; King's College London Faculty of Life Sciences and Medicine, School of Life-course and Nutritional Sciences<br>Muniu, Erastus; Kenya Medical Research Institute<br>Harding, Seeromanie; King's College London Faculty of Life Sciences and Medicine
**Primary Subject Heading**:	Public health
Secondary Subject Heading:	Epidemiology, Qualitative research, Infectious diseases
Keywords:	COVID-19, EPIDEMIOLOGY, Public health < INFECTIOUS DISEASES, PREVENTIVE MEDICINE

SCHOLARONE™
Manuscripts

I, the Submitting Author has the right to grant and does grant on behalf of all authors of the Work (as defined in the below author licence), an exclusive licence and/or a non-exclusive licence for contributions from authors who are: i) UK Crown employees; ii) where BMJ has agreed a CC-BY licence shall apply, and/or iii) in accordance with the terms applicable for US Federal Government officers or employees acting as part of their official duties; on a worldwide, perpetual, irrevocable, royalty-free basis to BMJ Publishing Group Ltd ("BMJ") its licensees and where the relevant Journal is co-owned by BMJ to the co-owners of the Journal, to publish the Work in this journal and any other BMJ products and to exploit all rights, as set out in our licence.

The Submitting Author accepts and understands that any supply made under these terms is made by BMJ to the Submitting Author unless you are acting as an employee on behalf of your employer or a postgraduate student of an affiliated institution which is paying any applicable article publishing charge ("APC") for Open Access articles. Where the Submitting Author wishes to make the Work available on an Open Access basis (and intends to pay the relevant APC), the terms of reuse of such Open Access shall be governed by a Creative Commons licence – details of these licences and which Creative Commons licence will apply to this Work are set out in our licence referred to above.

Other than as permitted in any relevant BMJ Author's Self Archiving Policies, I confirm this Work has not been accepted for publication elsewhere, is not being considered for publication elsewhere and does not duplicate material already published. I confirm all authors consent to publication of this Work and authorise the granting of this licence.

For peer review only

Contextually Appropriate Communication Strategies for COVID-19 prevention in Kenya Border Regions: Evidence from a mixed methods observational study in Busia and Mandera Counties

Kaduka Lydia, Olale Joanna, Karamanos Alexis, Mutai Joseph, Coultas Clare, Ahmed Ismail, Lesperance Veline, Read Ursula, Dazzan Paola, Cruickshank Kennedy, Muniu Erastus, Harding Seeromanie.

Kaduka Lydia, Centre for Public Health Research, Kenya Medical Research Institute, Nairobi, Kenya.

Olale Joanna, Centre for Clinical Research, Kenya Medical Research Institute, Nairobi, Kenya.

Karamanos Alexis, School of Population Sciences and Health Services Research and School of Life Course Sciences, King's College London, London, UK.

Mutai Joseph, Centre for Public Health Research, Kenya Medical Research Institute, Nairobi, Kenya.

Coultas Clare, School of Education, Communication and Society, King's College London, London, UK.

Ahmed Ismail, Centre for Public Health Research, Kenya Medical Research Institute, Nairobi, Kenya.

Lesperance Veline, School of Population Sciences and Health Services Research and School of Life-Course Sciences, King's College London, London, UK.

Read Ursula, Department of Mental Health and Wellbeing, Warwick Medical School, University of Warwick, Coventry, UK.

Dazzan Paola, Department of Psychological Medicine, Institute of Psychiatry, Psychology and Neuroscience, King's College London, London, UK.

Cruickshank Kennedy, School of Life-course and Nutritional Sciences, Kings College London, London, UK.

Muniu Erastus, Centre for Public Health Research, Kenya Medical Research Institute, Nairobi, Kenya.

For peer review only - http://bmjopen.bmj.com/site/about/guidelines.xhtml

For peer review only

Harding Seeromanie, School of Population Sciences and Health Services Research and School of Life Course Sciences, King's College London, London, UK.

Corresponding author

Kaduka Lydia

Kenya Medical Research Institute

Centre for Public Health Research

P.O. Box 20752-00202, Nairobi, Kenya

Tel: (254)-2-2725016/7. Fax: (254)-2-2720030

Email: lkaduka@kemri.go.ke

Keywords: COVID-19, Border regions, Kenya

Word Count: 4457

For peer review only

ABSTRACT

Objectives: Kenya has long and porous borders with its neighbouring countries. These regions, predominantly inhabited by highly mobile rural communities with strong cross-border cultural ties, present major challenges in managing movement of people and COVID-19 preventive measures. Our study sought to assess knowledge of COVID-19 prevention behaviours, how these varied by socio-economic (SEC) factors, and the challenges of engagement and implementation, in two border counties of Kenya.

Methods: We conducted a mixed methods study using a household e-survey (Busia N=294; Mandera N=288; 57% females, 43% males), and qualitative telephone interviews (N=73: Busia 55; Mandera 18) with policy actors, healthcare workers, truckers and traders, and community members. Interviews were transcribed, English-translated and analysed using the Framework Method. Associations between SEC (wealth quintiles, educational level) and knowledge of COVID-19 preventive behaviours were explored using Poisson regression.

Results: Participants were mostly educated to primary school level (54.4% Busia, 61.6% Mandera). Knowledge of COVID-19 prevention varied by behaviour: hand washing - 86.5%, use of hand sanitiser -74.8%, wearing a face mask - 63.1%, covering the mouth when sneezing or coughing -56.3% and social distancing- 40.1%. Differences in knowledge by area, educational level and the wealth index were marked, greatest for Mandera, the less educated and the poor. Interviews with stakeholders revealed challenges in health messaging, psychosocial and socioeconomic factors, lack of preparedness for truck border crossings, language barrier, denial and livelihood insecurity as key challenges to engagement with and implementation of COVID-19 prevention behaviours in the border regions.

Conclusion: The influence of SEC disparities and border dynamics on knowledge and engagement with COVID-19 prevention behaviours call for contextually appropriate risk communication strategies that are cognisant of community needs and local patterns of information flow. Coordinating response measures across border points is crucial in winning communities' trust and maintaining essential economic and social activities.

For peer review only - http://bmjopen.bmj.com/site/about/guidelines.xhtml

STRENGTHS AND LIMITATIONS

- The study sampling strategy and high e-survey response rate (97%) were key strengths, which point to the versatility required in the conduct of research in emergency response with community engagement being central to the success of similar research projects.
- The study did not objectively assess the knowledge of preventive behaviours. To overcome the potential bias of self-reported information, qualitative interviews were used to explore factors influencing the ability of the community to adhere to preventive measures.
- Whereas Kenya's median age is 21, majority of the e-survey participants were $\geq$55 years. This group tends to be less tech savvy and may explain the trust hierarchy observed in this study.
- The study recorded a lower number of Mandera interviews. However, the context-specific challenges presented what may be considered socio-culturally appropriate recommendations to inform future local efforts.

INTRODUCTION

Evidence continues to accrue on the dramatic global direct and indirect impacts of the COVID-19 pandemic. The pandemic has put a toll on both the social and economic structures of societies, contributing to global social, economic and health disparities [1-3]. Kenya has a population of ≈48 million, median age 20.1 years and 66.2% living on <US $3.20 /day [4]. It has long and porous borders with Tanzania, Uganda, Ethiopia, South Sudan, and Somalia, predominantly inhabited by highly mobile rural communities with strong cross-border cultural ties. Eight weeks after the first reported COVID-19 case in Kenya on March 13th 2020, a quarter of all 781 reported cases came from these border counties [1]. A single-wave epidemic peaked within 100 to 200 days after the first reported case [3]. Recognising its vulnerability to COVID-19 due to its position as a major travel and transport hub and relatively weak health system, Kenya implemented several mitigation measures including: a dusk to dawn curfew; mandatory mask wearing; prohibition of large public gatherings; and closure of borders [5,6]. Indirect impacts of the measures resulted in increased poverty levels, disrupted livelihoods, deepened inequalities and decelerated progress towards the Sustainable Development Goals [7-9].

The needs of communities at border regions are an important consideration for Kenya and its neighbours due to their shared vision of regional health security. The porous borders in

For peer review only - http://bmjopen.bmj.com/site/about/guidelines.xhtml

the Mandera triangle (Kenya, Ethiopia, and Somalia borders) and Busia (Kenya -Uganda border) limits the ability of partner states to monitor and identify active cases during pandemics [10-15]. With only 18% of the Kenyan population fully vaccinated by January 19th 2022 [16], the mainstay of the response remained adoption of preventive behaviours to reduce transmission.

In this study, we aimed to assess the levels of knowledge of key COVID-19 prevention behaviours, and whether these varied by socioeconomic factors, and obtained qualitative narratives to illuminate contextual factors impacting on engagement with prevention measures in Busia and Mandera border counties of Kenya. These two border counties continue to record poorer health outcomes due to long-standing issues in accessing reasonable quality healthcare because of poor health infrastructure, remoteness from the capital city, and high poverty rates. They are also among the eight lowest ranked counties in Kenya with the least healthcare workers per capita [17].

METHODS

Study design

A convergent parallel mixed methods study design was used to collect both quantitative and qualitative data in the two counties between September and October 2020. The design allows concurrent collection and analysis of both data sets, and convergence at interpretation stage [18].

Study settings: Mandera County located in Northern Kenya shares a border of 861km with Ethiopia to the North and 682 km with Somalia to the East (Figure 1). It is located 1052km from Kenya's capital city Nairobi, covers an area of 25,798 km², has a population of 867,457, population density of 33 persons/km², high poverty level (77.6%), and is inhabited predominantly by Muslim Somalis. Mandera's main economic activities are pastoralism and cross-border trade. Of Mandera's 775,085 population aged $\geq$3 years, 72% have never been to a learning institution. Busia County located 475km from Nairobi, borders Lake Victoria to the North and Uganda to the West (total border distance, 772 Km). With a 1,700Km² area and population of 893,000, population density is 527 persons/km², and poverty level 69%. Its main economic activities are cross-border trade, fishing, and agriculture, and is inhabited predominantly by Christians. 12.5% of Busia's 820,788 population aged $\geq$3 years have never been to school [19].

Quantitative survey

For peer review only - http://bmjopen.bmj.com/site/about/guidelines.xhtml

Sample size and sampling procedure: A cross-sectional household e-survey targeting adults aged ≥18 years was undertaken in September 2020. Sample size calculation was based on a prevalence of 50% for the indicator of interest (knowledge) and a maximum relative standard error for the estimated proportion of 7% to give a sample size of 205. Assuming 20 participants per cluster (communities), an intra-class correlation coefficient between clusters of 0.01, and a non-response rate of 10%, the resulting sample size was 269 rounded off to 300 participants, equivalent to 15 clusters per county. A three-stage sampling process was used. The first stage involved simple random sampling of Community Health Units (CHUs) in each County. The second stage involved sampling households within CHUs using systematic sampling with a random start, and lastly respondent selection in sampled households using the Kish Grid method [20].

Data collection and analysis: The e-survey data was collected using the WHO COVID-19 Rapid Quantitative Assessment Tool (Supplementary File 1) adapted to the local context [21]. The study used the REDCap software [22] to automate and share the survey tool with study participants. The e-questionnaire was administered either electronically on a smartphone or via telephone interview for participants with literacy or technology challenges. The study worked with trained community health workers (CHWs) in verifying respondent's eligibility and access to a smart phone with internet connection. A REDCap link was shared which allowed the respondent to consent, fill and submit the e-survey. Due to erratic internet in the study sites, the participants were allowed to submit their survey forms any time within the day using a unique return code generated specific to each participant to safeguard completion. Technical support was offered via WhatsApp.

Outcome measures: Knowledge about COVID-19 related preventive health behaviours was assessed by asking survey participants the following question *"Do you know how to prevent COVID-19?"*, to which they responded with a yes/no on the following individual statements; prevent COVID-19 by (i) wearing a face mask, (ii) keeping a distance of 2 metres from others, (iii) covering the mouth and nose when sneezing or coughing, (iv) using a hand sanitiser, and (v) washing hands with soap and water regularly. Affirmative responses were categorised as the reference category. Exposure status was assessed by asking participants if they had experienced

For peer review only - http://bmjopen.bmj.com/site/about/guidelines.xhtml

any of the following symptoms - fever, cough, shortness of breath, sore throat, fatigue, loss of taste or smell, or eye infection, two weeks prior to the survey.

Correlates and confounders: Socio-economic circumstances (SEC) were measured using education and the wealth index, two commonly used SEC indices in Kenya [19]. The wealth index was calculated using data on household's ownership of 14 selected assets (Supplementary Table 1). The weights (factor scores) for each of the assets were generated through principal components analysis (PCA). Years of education and adult education were categorised as following; 1) Secondary education and above (more than 9 years of compulsory years of education), 2) upper primary education (5-8 years of compulsory education), 3) lower primary education (1-4 years of compulsory education) or/and Islamic education (Duksi or Madrassa), and 4) no education.

Participant's sex, age (categorised in 4 age bands 18-24, 25-54, 55-65 and 65+) and survey location (Mandera/Busia) were considered as potential confounders. Trusted sources of information, desire for additional information on COVID-19 and perception of risks stemming from SARS CoV2 were considered as potential mediators and were assessed by asking participants to respond with a yes/no to each category for the following questions: *"Which source/who do you trust the most to receive information related to the new Coronavirus?" with response categories* (i) the radio, (ii) the TV, (iii) the WhatsApp messenger, (iv) Community Health Workers (CHWs) and (v) religious leaders. Desire for additional information on COVID-19 was measured using the question: *"What more would you like to know about COVID-19?"* with response categories (i) Symptoms of the new Coronavirus, (ii) How it is transmitted, (iii) what to do if you have symptoms; Perceived risks from COVID-19 (i) *"How dangerous do you find the new Coronavirus?"*, (ii) *"Do you think you are likely to become sick with the new Coronavirus"*, and (iii) "*Do you think any of your family members are at risk of becoming sick from the new Coronavirus?"*. Responses to the first question were categorised as very dangerous, dangerous or "not at all". The latter two were combined due to small numbers reporting 'not at all'. Response to the other two questions consisted of yes, no and do not know. Information on gender, survey location, and age group were also collected.

For peer review only - http://bmjopen.bmj.com/site/about/guidelines.xhtml

Statistical analyses: All statistical analyses used the Stata V.16 software [23] and the statistical significance level set at 5%. The association between the exposures (wealth quintiles and educational level) and the outcomes of interest (knowing that COVID-19 can be prevented by specific behaviours) was explored using Poisson regression [24]. Poisson regression was preferred over logistic regression to allow relative risks to be presented, rather than odds ratios which are often misrepresented. All regression analyses applied survey weights to account for the higher proportion of women included in the survey (57% vs. 43% for males) and the higher proportion of survey participants with secondary education and above (28.5% vs. 23.7% for those with no education). The multivariable regression analysis adjusted for wealth quintiles, educational level, gender, survey location, age group, trusted sources of information about COVID-19, desired additional information on COVID-19 and perception of risks.

Qualitative interviews

Sampling procedure: Purposive sampling was used to identify and select respondents with professional or personal experience of COVID-19. Semi-structured, in-depth telephone interviews were done with policy makers, healthcare workers, truckers, traders and COVID-19 survivors and carers to assess their experiences and needs in relation to the pandemic and mitigation measures specific to border counties' populations. Interviewees were mapped using an initial stakeholders list which included the counties' leadership, health management teams, and the COVID-19 response teams, to which further additions were made through snowballing. Interviews were completed between September and October 2020.

Data collection and analysis**:** Key informant interviews were carried out to explore understanding and experiences of the pandemic (Supplementary File 2). The interviews were conducted in either Kiswahili or English based on preference of the interviewees, time range of 30-60 minutes per interview. Questions related to the following topics were explored: community response to, and experience of the pandemic; health systems' preparedness and response to the pandemic; and border-specific issues related to the pandemic (only data pertaining to prevention behaviors are presented in this paper). The recorded interviews were transcribed, translated, and checked for quality by the Kenyan research team, and a UK-based co-author (CC) who is fluent in Swahili. The transcripts were then analysed using the framework

For peer review only - http://bmjopen.bmj.com/site/about/guidelines.xhtml

method (Gale et al. (2013) [25]: open-coding of the data was undertaken in pairs or small teams (with at least one Kenyan, and one UK-based individual) so that sociocultural differences in interpretations of the data could be identified and engaged with. The team then met altogether to develop an analytical framework with key categories (i.e. groupings of codes that are clearly defined). The analytical framework was then applied to all of the transcripts. Illustrative quotes were charted into a framework matrix and the data was interpreted and organized into themes.

Ethical considerations: Informed consent was sought and obtained from participants prior to their participation. E-consenting was done on REDCap, which involved mandatorily displaying the informed consent document. At the end of the consent document, the participant would click a mandatory check box in which they either provided or withdrew consent. The survey was only accessible upon consent. The digital record that the box was checked was archived as evidence of consent.

Patient and Public Involvement: The research question was arrived at upon interrogating the existing covid-19 response measures and realising a potential gap in addressing the needs of border communities. The County administration assessed the relevance of the proposed research and gave permission for community participation in the study. Community health workers and village elders assisted in sensitising and mobilising community participation, participated in mapping and selection of households, and sampling of study participants. The study findings were discussed in stakeholder forums with representation from the counties, community-based organisations and members of health committees of the local legislature. Recommendation items actionable at county level were taken up by respective county teams for implementation.

RESULTS

Knowledge of key COVID-19 prevention behaviours in Busia and Mandera

We assessed levels of knowledge of key COVID-19 prevention behaviours, and whether these varied by socioeconomic factors. Table 1 shows the sample profiles for the 582 adults (Busia: 50.5%; Mandera 49.5%) who participated in the e-survey.

For peer review only - http://bmjopen.bmj.com/site/about/guidelines.xhtml

Table 1: Sample profiles in Busia and Mandera Counties

	Busia (N=294)	Mandera (N=288)	All (N=582)
	**Proportion (95% CI)**	**Proportion (95% CI)**	**Proportion (95% CI)**
**Age group**			
18-24 (Early working age)	8.6 (5.6 to 12 .9) §	1.4 (0.6 to 3.5)	5.0 (3.4 to 7.3)
25-54 (Prime working age)	6.8 (4.3 to 10.6)	7.2 (4.7 to 10.8)	7.0 (5.1 to 9.5)
55-64 (Mature working age)	59.9 (52.8 to 66.7)	52.1 (45 to 59.1)	56.0 (51 to 60.9)
65 plus (Elderly)	24.7 (18.5 to 32) §	39.3 (32.3 to 46.8)	32 (27.2 to 37.3)
**Education level**			
Secondary and above	40.9 (34.4 to 47.6) §	15.1 (11.4 to 19.9)	28.0 (24.0 to 32.3)
Primary 5-8 yrs	28.6 (23.6 to 34.1) §	3.4 (2 to 5.8)	16.0 (13.4 to 19.0)
Primary 1-4years/Duksi/Madrasa	25.8 (19.1 to 34) §	58.2 (51.6 to 64.5)	42.0 (36.9 to 47.3)
None	4.7 (3.0 to 7.4) §	23.3 (18.9 to 28.3)	14.0 (11.6 to 16.8)
**Wealth quintiles**			
Highest quintile	16.9 (2.5 to 12.6) §	23.2 (17.8 to 29.7)	20.1 (16.5 to 24.2)
2nd	21.5 (2.7 to 16.7) §	12.7 (9.1 to 17.4)	17.1 (14.0 to 20.7)
3rd	29.7 (3.4 to 23.4) §	16.3 (11.6 to 22.4)	23.0 (18.9 to 27.7)
4th	17.8 (2.7 to 13.1) §	18.4 (14 to 23.9)	18.1 (14.8 to 22.0)
Lowest	14 (2.4 to 10) §	29.4 (23.1 to 36.6)	21.7 (17.8 to 26.2)
**Preventative behaviours**			
Wash hands regularly with soap and water	95.9 (92.3 to 97.9) §	77 (70.4 to 82.5)	86.5 (82.6 to 89.6)
Cover mouth and nose when coughing or sneezing	64.3 (57.3 to 70.7) §	48.3 (41.3 to 55.3)	56.3 (51.4 to 61.1)
Hand sanitiser use	84.4 (78.7 to 88.9) §	65.2 (58.3 to 71.6)	74.8 (70.3 to 78.9)
Standing 2 meters away from other people	52.2 (45.3 to 59.1) §	27.9 (22.2 to 34.6)	40.1 (35.4 to 45.0)
Wear a face mask	70.9 (64.2 to 76.9) §	55.3 (48.2 to 62.3)	63.1 (58.2 to 67.9)
**Trusted source of information on COVID-19**			
Radio	92.8 (88.6 to 95.5) §	54.8 (47.8 to 61.7)	73.8 (69.4 to 77.8)
TV	51.6 (44.7 to 58.4) §	11.8 (7.9 to 17.4)	31.7 (27.3 to 36.4)
WhatsApp	25.2 (19.8 to 31.5)	19.1 (14.2 to 25.3)	22.2 (18.4 to 26.5)
Community Health Workers	56.4 (49.4 to 63.1) §	43.7 (37.0 to 50.7)	50 (45.1 to 55)
Religious leaders	33.3 (27.3 to 39.9) §	46.2 (39.3 to 53.3)	39.7 (35 to 44.6)
**Kind of information received**			
Symptoms of COVID-19	82.3 (76.5 to 86.9) §	60.3 (53.2 to 66.9)	71.3 (66.6 to 75.5)
How SARS CoV2 is transmitted	74 (67.5 to 79.5) §	40.9 (34.2 to 47.9)	57.4 (52.5 to 62.2)

For peer review only - http://bmjopen.bmj.com/site/about/guidelines.xhtml

	Busia (N=294)	Mandera (N=288)	All (N=582)
	**Proportion (95% CI)**	**Proportion (95% CI)**	**Proportion (95% CI)**
What to do if you have symptoms	65.7 (58.9 to 72) §	28.7 (22.7 to 35.6)	47.2 (42.3 to 52.2)
**Perception of risk**			
**How dangerous COVID-19 is?**			
Very dangerous	89.3 (84.4 to 92.8) §	73.2 (66.7 to 78.9)	81.3 (77.2 to 84.8)
Less dangerous/Not dangerous	10.7 (7.2 to 15.6) §	26.8 (21.1 to 33.3)	18.7 (15.2 to 22.8)
**Do you think any of your family member are at higher risk of becoming sick?**			
Yes	53.3 (46.4 to 60)§	20.4 (15.6 to 26.2)	36.8 (32.3 to 41.7)
No	13.8 (9.8 to 19.2)	13.8 (9.9 to 18.9)	13.8 (10.9 to 17.4)
Don't know	32.9 (26.8 to 39.6)§	65.8 (59.1 to 71.9)	49.4 (44.4 to 54.3)
**Do you think you are likely to become sick with COVID-19?**			
Yes	56.1 (49.2 to 62.8)§	16.3 (12.2 to 21.4)	36.2 (31.6 to 41)
No	14 (9.9 to 19.3)	16.8 (12.3 to 22.6)	15.4 (12.2 to 19.2)
Don't know	29.9 (24.1 to 36.6)§	66.9 (60.3 to 72.9)	48.4 (43.5 to 53.4)
COVID-19 symptoms			
Fever	12.9 (9 to 18.1)	20.2 (15.6 to 25.8)	16.5 (13.4 to 20.2)
Cough	12.5 (8.6 to 17.7)	21.1 (16.3 to 26.9)	16.8 (13.6 to 20.6)
Shortness of breath	7.7 (4.6 to 12.5)	9.9 (6.6 to 14.6)	8.8 (6.4 to 12)
Sore throat	7.1 (4.3 to 11.7)	6.4 (3.8 to 10.3)	6.7 (4.7 to 9.6)
Fatigue	5.2 (2.8 to 9.5)	4.5 (2.5 to 9.1)	4.9 (3.1 to 7.4)
Loss of taste or smell	5.9 (3.2 to 10.7)	2.1 (0.9 to 5)	4 (2.4 to 6.6)

§ 95% CIs do not overlap

Majority (95%) of the participants were 25 years and above, 14% lacked formal education. Socio-economic disadvantage was high in both locations, with just over a quarter in Busia and over half of those in Mandera reporting having received only Primary 1-4 years, Duksi or Madrasa education. Mandera was consistently more disadvantaged than Busia across all measures. Overall, 17% reported a fever and cough, while 4% reported loss of smell/taste two weeks before the survey.

In both areas, the most known common prevention behaviours identified were washing hands regularly 86.5% (Busia 95.9%, 95% CI 92.3-97.9; Mandera 77%, 70.4-82.5) and use of a hand sanitiser 74.8% (Busia 84.4%, CI 78.7-88.9; Mandera 65.2%, CI 58.3-71.6) and the least commonly

For peer review only - http://bmjopen.bmj.com/site/about/guidelines.xhtml

known was standing two metres away from others 40.1% (Busia 52%, 45.3-59.1; Mandera 28%, 22.2-34.6). Significantly lower proportions in Mandera than in Busia reported knowledge of prevention behaviours, having had secondary education, being in the most disadvantaged 4th and 5th wealth quintiles, having trust in COVID-19 related information from various sources including the radio and TV, and having received COVID-19 related knowledge on symptoms, transmission and what to do if they have symptoms. There were some notable differences for trusted sources of COVID-19 information. In Busia, 93% cited the radio as the most trusted source, whereas in Mandera the radio (55%), community health workers (44%) and religious leaders (46%) shared the top ranking for trusted source. Table 2 shows the influence of adjusting for SEC, trusted sources of information, additional information and perception of risk on the Mandera-Busia differences in knowledge of prevention behaviours.

Table 2: The influence of adjusting for socio-economic circumstances, trusted sources of COVID-19 information, and additional information and perception of risk on the Mandera-Busia differences in knowledge of prevention behaviours. Risk Ratio (95% confidence interval), Busia is the reference category.

	Mandera-Busia difference in prevention behaviour, adjusted for gender and age	+ education level and wealth quintiles	+ trusted sources of information about COVID-19 knowledge	+desired additional information on COVID-19, and perception of risk
Wash hands regularly with soap and water	0.80 (0.74 to 0.87)***	0.81 (0.74 to 0.89)***	0.81 (0.73 to 0.90)***	0.86 (0.78 to 0.96)**
Cover mouth and nose when coughing or sneezing	0.72 (0.61 to 0.87)***	0.81 (0.64 to 1.03)	0.87 (0.67 to 1.14)	0.99 (0.76 to 1.28)
Hand sanitiser use	0.77 (0.68 to 0.86)***	0.80 (0.69 to 0.92)**	0.88 (0.75 to 1.02)	0.99 (0.85 to 1.16)
Standing 2 meters away from other people	0.52 (0.40 to 0.68)***	0.61 (0.44 to 0.85)**	0.58 (0.40 to 0.84)**	0.71 (0.50 to 1.01)+
Wear a face mask	0.77 (0.66 to 0.90)**	0.97 (0.79 to 1.20)	0.93 (0.73 to 1.17)	1.06 (0.83 to 1.36)

*+ p <0.1 and >0.05, * p<0.05, ** p<0.01, *** p<0.001*

For peer review only - http://bmjopen.bmj.com/site/about/guidelines.xhtml

The lower knowledge of washing hands in Mandera compared with Busia remained after fully adjusting, with hardly any shift in the risk ratios. The differences between Mandera and Busia in knowledge about covering the mouth when coughing/sneezing and wearing a mask were no longer significant on adjustment for SEC, for using a sanitiser on adjustment for trusted sources of COVID information, and for keeping a 2 metres distance from others on adjustment for all confounders.

Based on the pooled Busia and Mandera sample and after adjusting for confounders including location, the knowledge of prevention behaviours did not vary by education (Supplementary Figure 1). In contrast, regardless of location those in the most disadvantaged 5th wealth quintile remained less likely to report knowledge of three of the five prevention behaviours – washing hands regularly (- 20%, 95% CI -7 to -31%), covering mouth and nose when sneezing or coughing (-34%, -10 to -53%) and standing two metres away from others (-39%, -4 to -61%).

Significant interactions were observed between quintiles of wealth and location for knowing that covering the mouth when coughing or sneezing (F=35.23, p<0.001) and keeping 2 metres distance (F=13.26, p=0.009) could prevent COVID-19. The most socio-economically disadvantaged participants in Mandera were 62% (95% CI 33%-79%) less likely to know that COVID-19 could be prevented by covering their mouth while coughing/sneezing, and 61% (26% - 89%) less likely to know that COVID-19 could be prevented by keeping 2 metres distant (See Supplementary Figure 2). No significant differences in the knowledge of this behaviours were observed between the least and the most socioeconomically disadvantaged in Busia.

Qualitative insights into factors impacting on engagement with prevention and control measures

A total of 73 qualitative interviews (Busia: n= 55; Mandera: n= 18) were carried out with policy actors (n=8), healthcare workers (n=34), truckers and traders (n=7), COVID-19 survivors and carers (n=8) and other community members including religious leaders, village elders, persons living with disability, and commercial sex workers (n=16) in the two counties. Age of interviewees ranged between 23-74 years.

The qualitative interviews provided insights into contextual factors which impacted on engagement with prevention and control measures. Table 3 provides a summary, including

For peer review only - http://bmjopen.bmj.com/site/about/guidelines.xhtml

quotes from the qualitative interviews, to illustrate factors impacting on engagement with prevention measures in the two border regions. Lack of trust in the national government and political leaders was noted as a key barrier to engagement with prevention measures in the two border regions. Participants reported that this lack of trust mainly stemmed from alleged misappropriations of funds and politicians not adhering to COVID-19 restrictions.

Other reported impacts included socioeconomic factors such as the loss of livelihoods (especially in the informal sector), and increased cost of living due to rising food prices occasioned by disruptions in local trade and food imports, which impacted COVID-19 prevention measures. Psychosocial factors such as the centrality of spiritual support and family social connections in communities' cultures and way of life conflicted with preventive measures.

Additionally, there was reported to be inadequate attention to providing targeted, context specific, accessible risk communication and public health advice which impacted on equitable understanding and adherence. Challenges included use of technical global pandemic language and procedures that were not translatable to the local setting; lack of community engagement in tailoring communication messages; lack of targeted messaging for the pastoralist community in Mandera; language barriers with French-speaking truckers from Congo and Rwanda; and misinformation within the community regarding COVID-19 symptoms and transmission. Some initiatives by the two county governments were reported to facilitate engagement with prevention, including informal provisions of hand washing stations at the border posts, markets, shops, and religious institutions.

Challenges specific to border areas included, lack of preparedness for truck border crossings which led to pooling of truckers at the border especially in Busia, thus risking community transmission. Cross-border collaboration in enforcing the preventive measures was enhanced through leveraging an already existing infectious disease surveillance network in Mandera but challenged by stigmatization of truckers in Busia.

For peer review only - http://bmjopen.bmj.com/site/about/guidelines.xhtml

Table 3: Qualitative Insights on impacts on adherence to prevention measures
* (B/M) indicates location (Busia/Mandera), (M/F) indicates gender (male/female).

THEMES	CATEGORIES	ILLUSTRATIVE QUOTES
**Distrust of national government and politicians**	Politicians not adhering to COVID restrictions	BM-Imam-118: "They look at the politicians and how they are conducting themselves. Those breaking the rules do so because those that are supposed to encourage them are not adhering to the rules"<br>BM-Comm.HealthVolunteer-018: "people see the leaders walking without wearing masks [… so] they believe there is no corona"<br>MM-Trader-074 "we see politicians doing things without social distance… it only means there is no COVID"
	Media coverage of misappropriation of COVID funds	BM-Comm.HealthVolunteer-003: "having heard that there is a lot of money that was squandered through Kenya Medical Supplies Authority… majority of them are not doing the right thing [i.e. taking preventative measures]"<br>BM-PublicHealthOfficer-052: "people started relaxing in adhering after this scandal was unearthed whereby they say that billions of money was stolen"
**Health messaging not adequately contextualised to local communities**	Insufficient attention to impact of isolation on livelihoods	BM-COVIDSurvivor-111: "people fear to go and test, because they know you will just dump somebody there [in an isolation centre] for ten days […] you don't know the person, the way they eat, survive, he is just there like that in the name of quarantine, isolation, all those things"
	Inadequate community engagement and tailored public health messaging	BM-Policyactor-087: "we need to have more community engagement, with interpersonal communication kind of approach, so that you do more than just general messages, you are doing specific messages to specific groups of people, truck drivers, sex workers, maybe clearing agents and so on"<br>BM-Policyactor-113: "Communication on protection was not well done. We needed more of community engagement and good communication to ensure that all issues are clarified through question and answers"<br>BM-Policyactor-096: "remember the kind of information which was being given out initially [… led] by the Ministry of Health was more of .. well it was a caution but it really sounded to the public like this thing is going to wipe away so many people […] the words were really confusing, quarantine, isolation and so on"
	Circulation of misinformation in the community	- 'COVID is just like malaria, it should not be feared' (BM-Farmer-085; BF-Comm.HealthVolunteer-004).<br>'COVID only affects people in big cities and truckdrivers' / 'people do not know people who have had COVID - seeing is believing' (BF-Policyactor-025; BM-CommercialSexWorker-068; MM-Policyactor-007).
	Difficulties engaging with pastoralist communities (in Mandera)	MM-Policyactor-112: "the pastoralist nature of our society, you know people moving from here to the other side [of the border], uncontrollable has also been a serious issue [… they get health information from listening to] vernacular radio stations […] [but] compliance is another thing"<br>MM-Policyactor-007: "we had cases that were imported from Somalia by people who went to sell their livestock [and crossed the border unchecked]"
**Psychosocial and socioeconomic impacts on**	Desire for spiritual and social connection	BM-Policyactor-087: "people are social they want to be together but now they have been told to keep a distance… And when churches were closed, this was very strange… you will find it is affecting people spiritually"<br>BM-Policyactor-087: ""where people used to greet you by shaking hands, and now people are supposed to learn a new mode of greeting which is not our culture and to kick Kenyan culture is not so easy"

For peer review only - http://bmjopen.bmj.com/site/about/guidelines.xhtml

**preventive measures**		MF-COVIDSurvivor-098: "To protect yourself from corona, you are supposed to wear masks, don't greet people and also restrain from hugging people but sometimes you forget and greet people using your hands, you hug people because you are used to this"
	Socioeconomic barriers	BM-PLWDisability-117: "where will we disabled people get masks from? […] We have to work hard and buy for ourselves from our own pockets, buy for the children"<br>MM-Healthworker-037: "there's nobody who can provide them with masks, they were buying at […] twenty shillings or fifty shillings, and so it was very expensive?'"<br>MM-Policyactor-112: "There are these small [informal] businesses [e.g. tea/coffee stands], and… when we want to enforce those [social distancing] regulations it becomes very difficult because this is somebody's daily bread".
	Religious and social spaces supporting adherence	MM-Trader-074: "Even at the shop I have sanitiser, running water with soap, so people when they come to the shop they sanitize".<br>MF-COVIDSurvivor-098: "We have [hand]washing stations in hospitals, madrasa, at the mosque"<br>MM-PublicHealthOfficer-039 "we had a meeting with all the religious leaders… [to discuss how some preventive] measures… So after sensitizing, at least people have started accepting [preventive measures], because it was a big challenge for people to accept social distance in the place of worship"
**Issues specific to border areas**	Lack of preparedness for truck border crossings	BM-Policyactor-096: "initially we had lots of challenges... the trucks piled up… [resulting] in drivers not being able to cross over to Uganda on time, they ended up mingling with the rest of the population and so we knew it was just a matter of time before we got the first community cases"<br>BM-PublicHealthOfficer-052: ""you find this community transmission tends to be growing [because truckdrivers must wait for 2-3 days for the COVID test results]… we feel that there is a need for testing to be done across the entire [border] township […] but we lack the resources for doing this"
	Health prevention information not translated into all needed languages	BM-Policyactor-087: "people from Congo, from Rwanda, they speak French and […] most of our communications are in Kiswahili and English and therefore—like now we don't have any material for French here at the border and that is a huge challenge"
	Lack of consistency in cross-border communication	MM-Policyactor-007: "previous cooperation about polio issues […] can also help in responding to this pandemic"<br>BM-PublicHealthOfficer-052: "It is compulsory in Kenya to put on masks…these drivers when in Uganda they are not allowed to mingle with people, they think that anybody coming from Kenya is having Corona [because of the mask wearing]"<br>BM-Policyactor-113: "Kenya and Uganda might not have been reading from the same script. The countries should have the same lab where they collect the samples and the same approach to the communities. I believe they have community health issues at the border points [like us]. In times of a pandemic, no one is to blame. We have our own challenges but those that spread across East Africa would be dealt better with collaboration"
	Disruptions to cross-border trade and food security	BM-TraditionalHealer-072: "you can't cross the border to go to the Ugandan side [of the forest to collect herbs], if you are found, maybe you passed sideways, you get arrested. We had to stop working"<br>BM-Comm.HealthVolunteer-003: "there is food insecurity, people used to do farming freely, do business, they are unable to cross the border"

For peer review only - http://bmjopen.bmj.com/site/about/guidelines.xhtml

DISCUSSION

We assessed the levels of knowledge of key COVID-19 prevention behaviours in the two border counties. The best known preventive behaviour was washing of hands regularly and the least known was standing two metres apart, with Mandera significantly disadvantaged. We observed disparate knowledge of COVID-19 prevention measures based on socioeconomic status. Those with less education and those in lower socioeconomic groups were less likely to adhere to COVID-19 prevention measures. Key factors impacting on engagement with COVID-19 prevention measures included lack of trust in the leadership, deficits in public health communication, livelihood insecurity, prioritization of social support over quarantine/social distancing, and border-specific complexities such as language barriers and congregating of truckers due to lack of preparedness for truck border crossings.

Influence of socioeconomic inequalities on knowledge of COVID-19 prevention behaviours

Our study findings are consistent with previous studies in Kenya and other parts of Africa that observed variations in knowledge of COVID-19 prevention measures based on employment status, access to information, education level, geographical location and gender [26-28,]. A study in coastal Kenya [29] reported 60% knowledge of COVID-19 prevention measures, citing inequalities in knowledge, awareness and reach of health information.

The high awareness of COVID-19 reported in this study can largely be attributed to adoption of a whole of government and multi-agency approach (policy and technical) in response to the COVID-19 threat, accompanied by widespread media campaigns and news reports. A long history and experience with infectious disease response may have made messaging on hand hygiene and cough/sneeze etiquette more acceptable and readily implementable in Kenya. This however was not achieved with wearing masks and maintaining social distance as they were considered novel interventions. Lags in supply and affordability of masks and sanitising agents, and difficulties in sustaining lockdowns and movement restriction as reported in this study and elsewhere [14], contributed to failure in observing regular use of masks and social distancing. Low adherence to mask wearing and social distancing at a time of widespread consensus on COVID-19 being an airborne disease coupled with the low vaccination rate [16], call for innovative ways to engage communities in developing contextually relevant public health measures, particularly when

For peer review only - http://bmjopen.bmj.com/site/about/guidelines.xhtml

introducing novel interventions, cognisant of the influence of local culture and the impact of the socio-economic environment on the capacity for adherence. Likewise, emphasis on building community knowledge and not just providing top-down instructions, engaging communities in the adaptation processes as previously shown in Bangladesh [30], has the potential to improve adherence and ownership through better understanding. Further, the reported need for additional information on the disease indicates a lag in adjusting the communication messaging to the evolution of the pandemic, a gap that requires addressing.

Public health information

Our study found that community engagement with preventive measures improved with access to appropriate messages from preferred channels of communication. Socio-economic inequalities observed were slightly mitigated by trust placed in the source of information (religious leaders, WhatsApp, radio, and community health workers), signalling the opportunity to capitalize on the trust hierarchy to facilitate adherence to preventive behaviours. The pandemic communication strategy needs to be tailored to the local context [31], and the dynamics of information flow to address gaps in information penetration and engagement. Key in this process is the translation of the standardised pandemic language and procedures to local contexts through participatory processes. In addition, the reported community's loss of trust in the genuineness of the government efforts warrants a concerted effort to win back the public to comply with COVID-19 prevention, possibly through dialogue. The pastoralist communities require special attention as a key population due to their nomadic way of life that can marginalize them in respect of public health awareness and prevention measures and could increase the risk of cross-border transmission and potential recurrent outbreaks [15]. The risk is exacerbated by limited access to water and sanitation. There is need therefore for concerted effort in improving health literacy through tailored education campaigns and messaging in the border regions, and especially among the pastoralists, supported by structural interventions to enable disadvantaged and marginalised communities adhere to prevention measures.

An added dimension to risk communication is the stability and strength of mobile network coverage, which tends to be poorer in rural areas. Representativeness in sampling of participants in both counties could reflect the relative ease of access to local radio when compared to

For peer review only - http://bmjopen.bmj.com/site/about/guidelines.xhtml

television and internet-based social media, in contrast to other work done in urban settings in Kenya [26-27].

Border dynamics

Border health security is key to safeguarding the EAC's economic and social security. Studies show that implementation of COVID-19 interventions are likely problematic for populations with weak health systems, high density housing, porous borders and insecurity [32]. The disruptions to trade and livelihoods have had profound effects on border communities [33]. Concerns over financial stability (loss of income and food security) have persisted over time. There is a need to economically empower and build resilience among border communities to withstand the current and similar shocks in future. The border counties had to cater for a bigger population than their official figures, and as such, budgetary allocation to these counties should recognize this reality. The mandatory testing of truck drivers caused a major backlog in Busia which lies on the Northern Transport Corridor. As gathering points for large numbers of people, border crossings are inherently a risk point for transmission of SARS Cov2. This calls for better organization in designating and preparing holding areas in border regions. This could have potentially protected the local communities in the initial phase of COVID-19 and later with established community spread, the uninfected drivers.

In addition to the harmonised testing protocol of truckers by Kenya and her neighbours, there is also a need to strengthen cross-border surveillance networks to support contact tracing to increase the likelihood of containment of outbreaks and reduce the risk posed by cross-border health threats [34]. Adherence to prevention measures and movement restrictions at the borders were reportedly undermined by porous borders and strong social ties between the cross-border communities. The COVID-19 preventive measures and mitigation measures should thus be accompanied by intensive community engagement to minimise a sense of alienation among these communities. The use of informal crossings should be mitigated by developing criteria accessible to all that recognizes the inherent economic and social challenges in the region.

STUDY LIMITATIONS

The study did not objectively assess the knowledge of preventive behaviours, nor sought participants' self-reported levels of adherence. To overcome the potential bias of self-reported

For peer review only - http://bmjopen.bmj.com/site/about/guidelines.xhtml

information, qualitative interviews were used to explore factors influencing ability of the community to adhere to preventive measures which would be useful going forward than adherence reported at a single time point. Adopting standardized international research tools enables comparison of findings even though the tools do not comprehensively capture all possible sources of information, reflecting an issue of adapting Western-designed surveys. Whereas Kenya's median age is 21, majority of the e-survey participants were ≥55 years. This group tends to be less tech savvy. To minimise the digital divide and barriers to participant participation, the study trained CHWs, deployed a 'light' data collection tool and adaptive workflows, and provided robust technical support. The study recorded a lower number of Mandera interviews due to the geographical expansiveness of the area. However, the context-specific challenges presented what may be considered socio-culturally appropriate recommendations to inform future local efforts.

CONCLUSION

This research highlights the importance of pandemic response and communication in engagement with COVID-19 preventive measures in border settings. As in other contexts, socioeconomic inequalities were reflected in knowledge of and adherence to COVID-19 prevention measures, with the less educated and those in lower socioeconomic groups at greatest risk. The influence of trusted sources of information in mitigating such inequalities demonstrate the need for tailored public health communication strategies that are cognisant and responsive to local context and patterns of information flow. Further effort needs to be made in risk communication by modifying health messaging in tandem with evolution of the pandemic and incorporating community communication needs. Improving health literacy may support better health outcomes for the population.

Livelihood insecurity directly prevents the most disadvantaged from adhering to prevention measures. Economic empowerment and building resilience against future shocks is thus an important investment the country needs going forward. Coordinating response measures across border points is crucial in winning the community's trust and maintaining essential economic and social activities.

For peer review only - http://bmjopen.bmj.com/site/about/guidelines.xhtml

CONTRIBUTORS: KL, OJ, KA, MJ, CC, AI, LV, RU, DP, CK, ME and HS participated in conceiving the study, implementation and drafting of the manuscript. All authors contributed to the interpretation and subsequent revision of the paper. All authors approved the final version of the manuscript.

COMPETING INTERESTS: None

FUNDING: This work was supported by the Kenya Medical Research Institute (KEMRI) Internal Research Grant number KEMRI/COV/SPEC/002.

DATA SHARING AGREEMENT: All data relevant to the study are included in the article or uploaded as supplementary information.

ACKNOWLEDGEMENTS: We are grateful to all the study participants from Busia and Mandera Counties who took time to participate in this study. We thank the Director KEMRI, the National Commission for Science, Technology and Innovation, and the County Governments of Busia and Mandera for granting permission and providing an enabling environment to undertake this study. Many thanks to our research collaborators at Kings College London for their technical guidance and support, and the KEMRI County Cluster Coordinators Mr. Surrow Adow and Ms. Olipher Makwaga for their support. We thank our research assistants Schiller Mbuka, Rodgers Ochieng, Melvine Obuya, Esther Andia, Miriam Bosire, Doreen Mitaru and Priscilla Maiga for their tireless efforts in bringing the study to fruition.

ETHICS APPROVAL: The study protocol was approved by the KEMRI Scientific and Ethics Review Committee (Ref. No. KEMRI/SERU/CPHR/4021), and the National Commission for Science, Technology and Innovation (Ref. No. 864492).

REFERENCES

1. World Health Organization. WHO Coronavirus (COVID-19) Dashboard | WHO Coronavirus (COVID-19) Dashboard With Vaccination Data. https://covid19.who.int/region/afro/country/ke Accessed 20th January 2022.
2. Pinchoff J, Austrian K, Rajshekhar N, et al. Gendered economic, social and health effects of the COVID-19 pandemic and mitigation policies in Kenya: evidence from a prospective cohort survey in Nairobi informal settlements. *BMJ Open* 2021;11:e042749.

For peer review only - http://bmjopen.bmj.com/site/about/guidelines.xhtml

3. Brand SPC, Ojal J, Aziza R, et al. COVID-19 transmission dynamics underlying epidemic waves in Kenya. *Science* 2021;374:989-994.
4. World Bank poverty and equity data http://povertydata.worldbank.org/poverty/country/KEN Accessed 28th May 2020.
5. The COVID 19 global pandemic: Impact to the economy and policy options, Parliamentary Budget Office (PBO), Special Bulletin No. 1/2020, Government of Kenya (http://parliament.go.ke/sites/default/files/202004/Special%20Bulletin%20Covid%2019%202.4.2020%20final.pdf)
6. The Seventh Presidential Address on the Coronavirus Pandemic: The 8-Point Economic Stimulus Programme. Government of Kenya (https://www.president.go.ke/2020/05/23/the-seventh-presidential-address-on-the-coronavirus-pandemic-the-8-point-economic-stimulus-programme-saturday-23rd-may-2020/)
7. World Bank. 2021. Socioeconomic Impacts of COVID-19 in Kenya on Households: Rapid Response Phone Survey, Round 1. (https://openknowledge.worldbank.org/handle/10986/35173 License: CC BY 3.0 IGO)
8. Shadmi E, Chen Y, Dourado I, et al. Health equity and COVID-19: global perspectives. *International Journal for Equity in Health* 2020; 19:10
9. Quaife M, van Zandvoort K, Gimma A, et al. The impact of COVID-19 control measures on social contacts and transmission in Kenyan informal settlements. *BMC Medicine* 2020; 18:316.
10. Ope, M, Sonoiya S, Kariuki J, et al. Regional Initiatives in Support of Surveillance in East Africa: The East Africa Integrated Disease Surveillance Network (EAIDSNet) Experience. *Emerging Health Threats Journal* 2013; *6*.
11. Okongo, O. Malaria pandemic , leading cause of deaths in Busia County. *Talk Africa* 2017. https://www.talkafrica.co.ke/reports-indicate-malaria-cases-cause-deaths-busia-county/
12. Nnnennaya A, & Hagembe J. Increasing Access to Healthcare Services for Cross-Border and Mobile Communities in East Africa – Global Health Innovation Center, 2019. (https://dukeghic.org/2019/07/19/increasing-access-to-healthcare-services-for-cross-border-and-mobile-communities-in-east-africa/)

For peer review only - http://bmjopen.bmj.com/site/about/guidelines.xhtml

13. World Health Organisation. Horn of Africa countries in a communique to speed up polio eradication. Regional Office for Africa. 2018. https://www.afro.who.int/pt/node/10538
14. East African Community. Mapping of Health Services along major transport corridors in East Africa. East African Community Report, 2015.
15. African Union. The COVID-19 Pandemic in the IGAD region: Impact on the Health and Economy Vol 1. Horn Economic and Social Policy Institute (HESPI): Pillar 2 An assessment of the health and economic impact on the HoA countries, 2020. (https://media.africaportal.org/documents/The-COVID-19-Pandemic-in-the-IGAD-region_VOL-I.pdf)
16. Kenya Ministry of Health. COVID-19 outbreak in Kenya. Daily Situation Report 673. 19th January 2022.
17. Kenya Ministry of Health. Kenya Harmonized Health Facility Assessment Report (KHFA) 2018/2019 Report.
18. Creswell, J. W., & Plano Clark, V. L. (2011). Designing and conducting mixed methods research. Thousand Oaks, CA: Sage.
19. Kenya National Bureau of Statistics. Kenya Population and Housing Census. Volume IV: Distribution of Population by Socio-Economic Characteristics 2019 (https://www.knbs.or.ke/?wpdmpro=2019-kenya-population-and-housing-census-volume-iv-distribution-of-population-by-socio-economic-characteristics)
20. McBurney P. On transferring statistical techniques across cultures: The Kish Grid. Current Anthropology 1998; 29:323-325.
21. World Health Organization. Risk Communication and Community Engagement (RCCE) Action Plan Guidance COVID-19 Preparedness and Response, 2020 (https://www.who.int/publications/i/item/risk-communication-and-community-engagement-(rcce)-action-plan-guidance)
22. Harris PA, Taylor R, Thielke R, et al. Research electronic data capture (REDCap)—A metadata-driven methodology and workflow process for providing translational research informatics support. *Journal of Biomedical Informatics* 2009; 42: 377–381.
23. StataCorp. 2019. Stata Statistical Software: Release 16. College Station, TX: StataCorp LLC.

For peer review only - http://bmjopen.bmj.com/site/about/guidelines.xhtml

24. Zou G. A modified Poisson regression approach to prospective studies with binary data. *Am J Epidemiol.* 2004;159(7):702–6.

25. Gale NK, Heath G, Cameron E, et al. Using the framework method for the analysis of qualitative data in multi-disciplinary health research. *BMC Medical Research Methodology* 2013; 13:117

26. Karijo E, Wamugi S, Lemanyishoe S, et al. Knowledge, attitudes, practices, and the effects of COVID-19 among the youth in Kenya. *BMC Public Health* 2021; 21(1):1020.

27. Austrian K, Pinchoff J, Tidwell JB, et al. COVID-19 related knowledge, attitudes, practices and needs of households in informal settlements in Nairobi, Kenya. [Preprint]. *Bull World Health Organ*. E-pub: 6 April 2020. doi: http://dx.doi.org/10.2471/BLT.20.260281

28. Sengeh P, Jalloh MB, Webber N, et al. Community knowledge, perceptions and practices around COVID-19 in Sierra Leone: a nationwide, crosssectional survey. *BMJ Open* 2020;10:e040328.

29. Mwai J, Mutai J, Kaduka L, et al. Association between sociodemographic characteristics and knowledge and practice of COVID-19 measures among households in Mombasa and Kilifi County, Kenya. *Int Health*. 2022:ihac049.

30. Abaluck J, Kwong LH, Styczynski A, et al. Impact of community masking on COVID-19: A cluster-randomized trial in Bangladesh. *Science* 2022;14;375(6577):eabi9069.

31. Sachs JD, Karim SSA, Aknin L, et al. The Lancet Commission on lessons for the future from the COVID-19 pandemic. *Lancet*. 2022;400:1224-1280.

32. Da'ar OB, Haji M & Jradi H. Coronavirus Disease 2019 (COVID-19): Potential implications for weak health systems and conflict zones in the Middle East and North Africa region. *Int J Health Plann Manage* 2020; 35(5):1240-1245.

33. Quaife M, van Zandvoort K, Gimma A, et al. The impact of COVID-19 control measures on social contacts and transmission in Kenyan informal settlements. *BMC Medicine* 2020; 18:316.

34. East African Community. Standard Operating Procedure - Cross-border Surveillance in the EAC Region East African Community (EAC) Secretariat. www.eac.int

For peer review only - http://bmjopen.bmj.com/site/about/guidelines.xhtml

Figure Legends

Figure 1: Map showing the location of Busia and Mandera Counties in Kenya

For peer review only

For peer review only - http://bmjopen.bmj.com/site/about/guidelines.xhtml

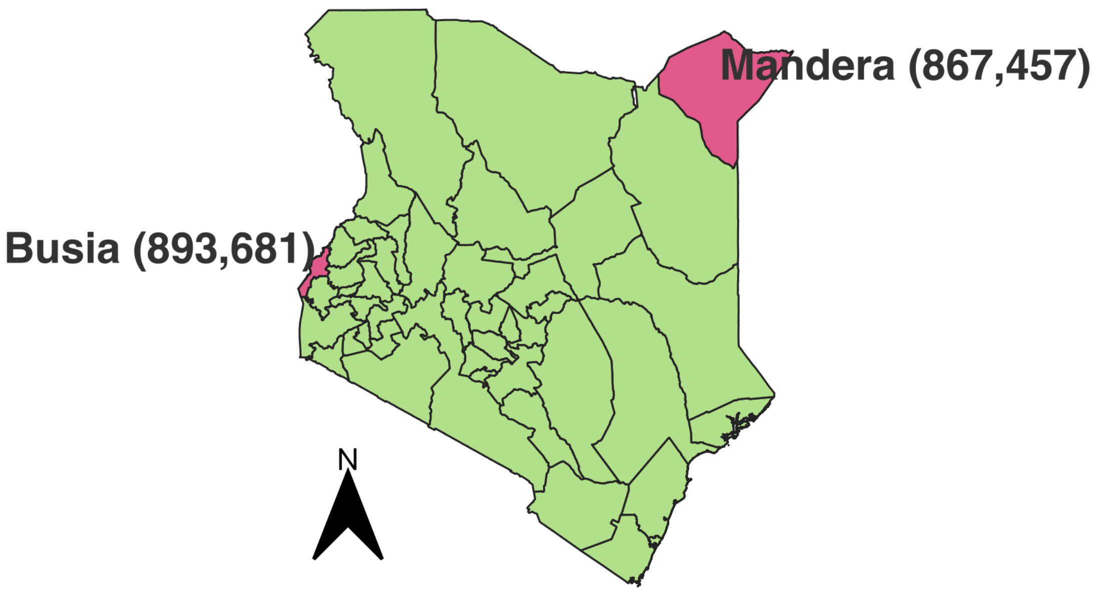
Mandera (867,457)
Busia (893,681)
N

COVID-19 RAPID QUANTITATIVE ASSESSMENT TOOL

ID of Study Participant: …………………………………………

Date of Interview: ………………………………………………

Physical Location: ………………………………………………

**1. SOCIO-DEMOGRAPHIC INFORMATION**		
1.1	Sex	[1] Male [2] Female
1.2	Age of respondent (in complete years)	
1.3	What is your current marital status?	[1] Single (not married and not living with a partner)<br>[2] married (monogamous)<br>[3] married (polygamous)<br>[4] Separated (currently not living together but not divorced)<br>[5] Divorced<br>[6] Widowed/widower<br>[7] Co-habiting (not married but live with a partner)
1.4	What level of education or years of schooling did you finish?	[1] None<br>[2] Primary education, 1-4 years<br>[3] Primary education, 5-8 years<br>[4] Secondary Education, 9-12 years<br>[5] Secondary Education, 13-14<br>[6] University Education > 14 years<br>[7] Adult education
1.5	What is your main occupation?	[1] Student<br>[2] Employed/Professional(Teacher, doctor, nurse, manager, accountant)<br>[3] Casual worker (attendant in hotel, bar, shop, housemaid, gardener)<br>[4] Housewife<br>[5] Business (self-employed)<br>[6] Farmer (large scale, subsistent farming, gardening)<br>[7] Unemployed<br>[8]Other(specify) ____________________

**2. SOCIO-ECONOMIC INFORMATION**		
2.1	Currently, who are you living with? (*Circle all that apply)*	[1] Alone<br>[2] Parents<br>[3] Spouse<br>[4] Children<br>[5] Other relatives<br>[6] Friends
2.2	What is the **MAIN source** of income for your household? (*Circle only one)*	[1] Formal employment<br>[2] Small scale farming<br>[3] Farming and selling of produce<br>[4] Small business, self-employed (non-farming)<br>[5] Petty trade<br>[6] Casual/informal labour<br>[7] Relatives/remittances<br>[8] Welfare/NGO support<br>[9] Other (specify)____________________
2.3	What is the **MAIN** source of food for your	[1] Purchase (market/grocery store)<br>[2] Household farm/garden

For peer review only - http://bmjopen.bmj.com/site/about/guidelines.xhtml

	household? (*Choose one response only*)	[3] Relatives and friends<br>[4] Welfare/NGO support<br>[5] Other (specify) ____________<br>[88] Don't Know
2.4	How much on average do you spend on food in your household? (*choose from categories*)	[1] Less than 1000<br>[2] 1,000-2,999<br>[3] 3,000-4,999<br>[4] 5,000-9,999<br>[5] 10,000-19,999<br>[6] 20,000-49,999<br>[7] 50,000- 99,999<br>[8] 100, 000+
2.5	What is the main source of your domestic water?	[1] Tap/rain water within compound (piped into the house)<br>[2] Tap water outside the house compound (public tap)<br>[3] Tap water, purchased from trolley (mkokoteni)<br>[4] Well/bore hole<br>[5] Pond water<br>[6] Other (specify) ______________________________________<br>[88] Don't Know
2.6	Do you usually treat water for drinking?	[1] Yes<br>[2] No<br>[88] Don't know
2.7	If **YES to 3.6**, how do you usually treat water for drinking?	[1] Boil<br>[2] Filter<br>[3] Boil and Filter<br>[4] Use bottled water<br>[5] Do nothing<br>[6] Other (specify) ___________________________________<br>[88] Don't Know
2.8	What kind of latrine/toilet facility do members of your household usually use?	[1] Flush or pour flush toilet<br>[2] Pit latrine<br>[3] Bucket toilet<br>[4] Hanging toilet/hanging latrine<br>[5] No facility/bush/field<br>[6]Other (Specify)_______________________________________
2.9	Do you share this toilet facility with other households?	[1] Yes<br>[2] No
2.10	Does your household have *(tick as many)*	[1] Clock/watch<br>[2] Electricity<br>[3] Radio<br>[4] Television<br>[5] Mobile telephone<br>[6] Fixed telephone<br>[7] Refrigerator<br>[8] Solar panel
2.11	Does any member of your household own:	[1] Bicycle<br>[2] Motorcycle/scooter<br>[3] Animal-drawn cart<br>[4] Car/truck

For peer review only - http://bmjopen.bmj.com/site/about/guidelines.xhtml

		[5] Boat with motor
2.12	What type of fuel does your household **MAINLY** use for cooking?	[1] Firewood<br>[2] Charcoal<br>[3] Kerosene<br>[4] Electricity<br>[5] Gas<br>[88] Other (Specify)________________________________
2.13	The house you live is it,	[1] Rented<br>[2] Live without pay?<br>[3] Owns<br>[4] Squatting
2.14	How many sleeping rooms are there in the house you live?	[1] Zero or one sleeping rooms<br>[2] Two sleeping rooms<br>[3] Three or more sleeping rooms.
2.15	What quality /type of material is the floor of the house you live in?	[1] Finished floor with parquet, carpet, tiles, ceramic Tiles<br>[2] Cement, concrete, raw wood, etc<br>[3] None, earth, dung Wood<br>[4] Other (specify) ________________________
**3. KNOWLEDGE ABOUT COVID-19**		
3.1	Have you heard about the new Coronavirus disease (COVID-19)	[1] Yes<br>[2] No
3.2	What do you know about the new coronavirus disease? (Choose all that apply)	Choose one best answer:<br>**[1]** I don't know anything<br>**[2]** It's a virus that can cause a disease<br>**[3]** It's a government's programme<br>**[4]** It's a TV/radio campaign<br>**[5]** Other: Specify________________
3.3	What kind of information have you received about the disease? (Legends to be added to the questionnaire)	Mark all answers that are true:<br>**[1]** How to protect yourself from the disease?<br>**[2]** Symptoms of the new coronavirus disease<br>**[3]** How it is transmitted<br>**[4]** What to do if you have the symptoms<br>**[5]** People who are at high risk of getting the disease<br>**[6]** Complications of the disease<br>**[7]** Other (please specify)_________

For peer review only - http://bmjopen.bmj.com/site/about/guidelines.xhtml

3.4	Where did you hear about the new coronavirus disease from? What channels or sources? (Tick all that apply)	Mark all channels that you heard about the disease from:<br>**[1]** Radio<br>**[2]** TV<br>**[3]** WhatsApp<br>**[4]** Social Media (e.g. Facebook, Instagram, Twitter)<br>**[5]** Health unit/Healthcare worker<br>**[6]** Family members<br>**[7]** Friends<br>**[8]** Community health workers<br>**[9]** Red Cross/ Red Crescent volunteers<br>**[10]** Other community mobilisers<br>[11] Community leaders<br>[12] Religious Leaders<br>[13] Traditional Healers<br>[14] Traditional Midwives<br>[15] Any other person from the community<br>[16] Other (please specify) __________
3.5	Which channel/who do you trust the most to receive information related to coronavirus? (one or more options)	Check the best channel for you to receive coronavirus information from:<br>**[1]** Radio<br>**[2]** TV<br>**[3]** WhatsApp<br>**[4]** Social Media (not WhatsApp)<br>**[5]** Health unit/Healthcare worker<br>**[6]** Family members<br>**[7]** Friends<br>**[8]** Community health workers<br>**[9]** Red Cross Red Crescent volunteers<br>**[10]** Other community mobilisers<br>[11] Community leaders<br>[12] Religious Leaders<br>[13] Traditional Healers<br>[14] Traditional Midwives<br>[15] Any other person from the community<br>[16] Other (Specify) ____________________
3.6	How dangerous do you think the new coronavirus risk is?	Choose the best answer that matches your belief about the disease:<br>**[1]** Very dangerous<br>**[2]** More or less dangerous<br>**[3]** Is not dangerous<br>**[4]** Other (Specify)______________________
3.7	Who do you think is at highest risk to get the coronavirus?	

For peer review only - http://bmjopen.bmj.com/site/about/guidelines.xhtml

		Mark all those you think are likely to become sick:<br>**[1]** Children under 5 years old<br>**[2]** Adolescents up to 15 years old<br>**[3]** Youth<br>**[4]** Adults<br>**[5]** Elderly persons<br>**[6]** Pregnant women<br>**[7]** Health workers<br>**[8]** Persons with chronic diseases such as diabetes and high blood pressure<br>**[9]** Other (Specify)_________________
3.8	Do you think you are likely to become sick with the new coronavirus?	[1] Yes<br>[2] No<br>[3] Don´t know<br>If Yes or No, tell us Why_
3.9	Do you think any of your family members are at risk of becoming sick with coronavirus?	[1] Yes<br>[2] No<br>[3] Don´t know<br>If Yes or No, tell us Why_
3.10	How does the coronavirus spread?	Mark all the ways you think the disease spreads:<br>**[1]** Blood transfusion<br>**[2]** Droplets from infected people<br>**[3]** Airborne<br>**[4]** Direct contact with infected people.<br>**[5]** Touching contaminated objects/surfaces<br>**[6]** Sexual intercourse contact<br>**[7]** Contact with contaminated animals<br>**[8]** Mosquito bites<br>**[9]** Eating contaminated food<br>**[10]** Drinking unclean water<br>**[11]** Don't know<br>[12] Other (Specify)_________________
3.11	What are the main symptoms of COVID-19?	Mark all the symptoms you think are caused by the new coronavirus:<br>**[1]** Fever<br>**[2]** Cough<br>**[3]** Shortness of breath and breathing difficulties<br>**[4]** Muscle pain<br>**[5]** Headache<br>**[6]** Diarrhoea<br>**[7]** Don't know<br>**[8]** No symptoms<br>**[9]** Other (Specify) _________________________

For peer review only

For peer review only - http://bmjopen.bmj.com/site/about/guidelines.xhtml

3.12	Do you know how to prevent it? (One or more options)	Choose all the prevention steps you know about:<br>**[1]** Sleep under the mosquito net<br>**[2]** Wash your hands regularly using soap and water<br>[3] Use hand sanitiser<br>**[4]** Drink only treated water<br>**[5]** Cover your mouth and nose when coughing or sneezing<br>**[6]** Avoid close contact with anyone who has a fever and cough<br>**[7]** Eliminate standing water<br>**[8]** Cook meat and eggs well<br>**[9]** Avoid unprotected direct contact with live animals and surfaces in contact with animals<br>**[10]** Standing 2 metres away from other people<br>**[11]** Wearing a mask<br>**[12]** Don't know<br>**[13]** Other Specify___________________________
3.13	What have you and your family been doing to prevent becoming sick with coronavirus in the recent days? (Tick all that apply)	Mark all steps that you have taken:<br>**[1]** Washing hands regularly using soap / water<br>[2] Using alcohol-based hand sanitiser<br>**[3]** Covering mouth and nose when coughing or sneezing<br>**[4]** Avoid close contact with anyone who has a fever and cough<br>**[5]** Eliminate standing water<br>**[6]** Cook meat and eggs well<br>**[7]** Avoid unprotected direct contact with live animals and surfaces in contact with animals.<br>**[8]** Standing 2 metres away from other people<br>**[9]** Wearing a mask<br>**[10]** Don't know<br>**[11]** Nothing<br>**[12]** Other (Specify)_________________________________
3.14	If Nothing to question 3.13, what makes it difficult for you to follow advice on preventing yourself from the disease?	**[1]** Need to go to work to earn money<br>**[2]** Live with a large family in a small house<br>**[3]** Don't know<br>**[4]** Other (Please specify)_____________________
3.15	Do you consider it important to take actions to prevent the spread of coronavirus in your community?	[1] Yes<br>[2] No<br>[3] Don't know<br>[4] Other (Specify) ________________________
3.16	What would you do if you or someone from your family has symptoms of this disease?	

For peer review only - http://bmjopen.bmj.com/site/about/guidelines.xhtml

		Tick all the actions you would take:<br>**[1]** I will look for a more experienced relative to advise me on what to do<br>**[2]** I will go to the hospital / health unit<br>**[3]** I will go to the neighbourhood nurse<br>**[4]** I will buy medicines at the market<br>**[5]** I will look for the traditional healer<br>**[6]** I would stay in quarantine<br>**[7]** Other (Specify) ______________________________
3.17	What more would you like to know about the disease?	Choose all topics that you would like to know about;<br>**[1]** How to protect yourself from the disease?<br>**[2]** Symptoms of the new coronavirus disease<br>**[3]** How it is transmitted<br>**[4]** What to do if you have the symptoms<br>**[5]** Most at risk groups<br>**[6]** How to treat it<br>**[7]** Other (Specify)___________________________
3.18	Do you think the coronavirus disease is generating stigma against specific people?	[1] Yes<br>[2] No<br>[3] Don't know
3.19	(If yes) Which group is being dis-criminated in your community because of coronavirus?	1.____<br>2.____<br>3.____
3.20	Do you think Kenya is adequately prepared in dealing with this disease?	[1] Yes<br>[2] No<br>[3] Don't know
3.21	Do you know of any special measures that have been put in place nationally to deal with this new coronavirus? (Tick all that apply)	[1] Social distancing<br>[2] Closing borders<br>[3] Coronavirus telephone line/help<br>[4] Temporary expansion of healthcare facilities<br>[5] National Curfew<br>[6] Other (please specify)
3.22	If restrictions are in place, have you had difficulty in acquiring essential supplies such as food?	[1] Yes<br>[2] No<br>[3] Don't know
3.23	How do you think vulnerable groups can access essential supplies such as food and medicine? (Tick all that apply)	(1). Friends and family<br>(2). Community programmes<br>(3). National government run service<br>(4). NGO<br>(5). Religious organisations<br>(6). Other (please specify)

For peer review only - http://bmjopen.bmj.com/site/about/guidelines.xhtml

4. KNOWLEDGE ABOUT SIMILAR DISEASES: SEASONAL OR REGULAR FLU		
4.1	What do you usually do if you have a regular flu?	Mark the 1-2 most typical actions you take:<br>**[1]** I have never had the flu<br>**[2]** Nothing, I continue with my normal life, after some time the flu goes away<br>**[3]** I stay at home, so I don't infect others<br>**[4]** I wear a mask<br>**[5]** I drink tea until I recover<br>**[6]** I seek advice from my neighbours<br>**[7]** I seek advice from local healers<br>**[7]** I go to the health unit<br>**[8]** Other (Specify) ___________________________________
4.2	From where/who do you usually receive information on flu and other diseases?	Choose as many channels as you have heard about regular flu from:<br>**[1]** Radio<br>**[2]** TV<br>**[3]** WhatsApp<br>**[4]** Social Media (not WhatsApp)<br>**[5]** Health unit/Healthcare worker<br>**[6]** Family members<br>**[7]** Friends<br>**[8]** Community health workers<br>**[9]** Red Cross Red Crescent volunteers<br>**[10]** Other community mobilisers<br>[11] Community leaders<br>[12] Religious Leaders<br>[13] Traditional Healers<br>[14] Traditional Midwives<br>[15] Any other person from the community<br>[16] Other (Specify) __________________

<u>THANK YOU VERY MUCH FOR YOUR TIME</u>

For peer review only

For peer review only - http://bmjopen.bmj.com/site/about/guidelines.xhtml

COVID-19 QUALITATIVE INTERVIEW GUIDE

This will be a semi structured interview with people who have knowledge of and experienced the COVID-19 disease. We wish to obtain views/opinions from policy actors, healthcare workers, COVID-19 survivors and carers, COVID-19 bereaved, truckers and traders.

Introductory Script

Enumerator:
Good morning/ afternoon,
My name is {**Enumerator Name**} from **KEMRI**.
Welcome to this session and thank you for taking the time to be here. We are conducting a research study on behalf of KEMRI and Kings College London (UK) to assess the response strategies and community/practitioner experiences of COVID-19 in border counties in Kenya.

We would like you to be as honest and as open as you can. Also take note that there are no correct, right or wrong response(s) to the issues raised. Feel free to chip in or interject at any point. All your responses will be both confidential and anonymous, and your name and details will not be divulged during analysis. Your responses will not affect your standing with anyone or any organization/company mentioned in this discussion.

In order to make sure that we capture the most useful account of this discussion, we would like to audio record our conversation. This recording will only be available to the research team involved. Will you give us permission to record?
Thank you.

Demographic Information

1. Participant geographic Location
2. Sex
3. Age
4. Occupation
5. Level of Education

Awareness of COVID-19

1. Have you heard about the New Coronavirus Disease (COVID-19)? Please tell me what you know about it (*Explain to the participant what the disease is in case s/he doesn't know what it is*)
2. From whom/ where did you get this information from (*Prompt for details of the information given through the specified source e.g. symptoms, transmission, self-care, risks, complications etc.*)

For peer review only - http://bmjopen.bmj.com/site/about/guidelines.xhtml

3. Are there some people, information sources or channels that you trust more than others to give you accurate information about the disease? Which people or sources are these and why do you trust them?

4. In your view how dangerous do you think the COVID-19 disease is? (*Probe for reasons as to why*)

5. Who in your view do you think is most at risk of contracting this disease? (*Probe for reasons as to why*)

6. Please explain to me how you think the COVID-19 is spread in in your County?

7. In your view, how would you identify someone who is infected with the disease? (*Probe for main symptoms*)

8. Do you think a person can be infected and spread the infection to others before they start showing the symptoms you have just mentioned? (*probe for reason for their opinion*)

9. Have you heard of any test that is done by medical professionals to identify people who have the disease? (*probe for what they know about testing*)

10. How would you prevent yourself or your family from contracting this disease?

11. What have you been doing to prevent yourself and your family from contracting the disease? (*Probe for prevention measures*)

12. What role can you play to prevent the spread of the disease within your community? (*Probe for self-quarantine measures, creation of awareness*)

13. What would you do if a member of your family contracted the disease?

14. Where do you think would be the best place for your family member to receive treatment if they contracted the disease? If it is a place away from home, would you be able to take them there? (*probe for why this is so both yes and no answers*)

15. What myths and misconceptions have you heard about the disease? (Probe for myths and misconceptions around symptoms, transmission, prevention, treatment)

16. Is there any more information you would like to know about the disease? (*Probe for preferred source for the additional information*)

17. In your view, do you think the COVID-19 disease pandemic is generation stigma against specific people? (*Probe the specific groups of people being stigmatised and how they are being stigmatized*)

18. Have you ever been sick with seasonal or regular flu? What do you usually do when you have a seasonal flu? (*Probe for home remedies, seeking care from hospital, doing nothing about it etc.*)

For peer review only - http://bmjopen.bmj.com/site/about/guidelines.xhtml

Additional questions for policy actors and healthcare workers

19. What is your role in the current organization that you serve?
20. How would you describe the preparedness and response of Kenya's healthcare system to COVID 19?
21. What has been your personal experience in regard to COVID-19?
22. What are some of the facilitators and barriers in the healthcare system when it comes to dealing with COVID-19 pandemic?
23. What would you wish to see done differently?

Thank you very much for your time and response.

For peer review only

For peer review only - http://bmjopen.bmj.com/site/about/guidelines.xhtml

Supplementary Table 1: List of variables used for constructing a wealth index

	Name of Variable	Coding		Factor loadings	
				Factor1	Factor2
1	Ownership of household goods - 1 Clock/Watch	1 = Yes	0 = No		
2	Ownership of household goods - 2 Electricity	1 = Yes	0 = No	.708	
3	Ownership of household goods - 3 Radio	1 = Yes	0 = No		.657
4	Ownership of household goods - 4 Television	1 = Yes	0 = No	.628	.427
5	Ownership of household goods - 5 Mobile telephone	1 = Yes	0 = No	.375	
6	Ownership of household goods - 6 Fixed telephone	1 = Yes	0 = No		
7	Ownership of household goods - 7 Refrigerator	1 = Yes	0 = No		
8	Ownership of household goods - 8 Solar panel	1 = Yes	0 = No		.552
9	Any household member own - 1 Bicycle	1 = Yes	0 = No		.753
10	Any household member own - 2 Motorcyle/Scotter	1 = Yes	0 = No	.427	
11	Any household member own - 3 Animal drawn cart	1 = Yes	0 = No		
12	Any household member own - 4 Car/Truck	1 = Yes	0 = No		
13	Any household member own - 5 Boat with motor	1 = Yes	0 = No		
14	Own house live in	1 = Yes	0 = No		.346
15	Sleeping rooms in the house that live in	1, 2, 3		.640	
16	Finished/cement floor	1 = Yes	0 = No	.681	
	Small coefficients below .30 were suppressed<br>*Eigen values for components one and two were 2.464 & 1.509*				

For peer review only

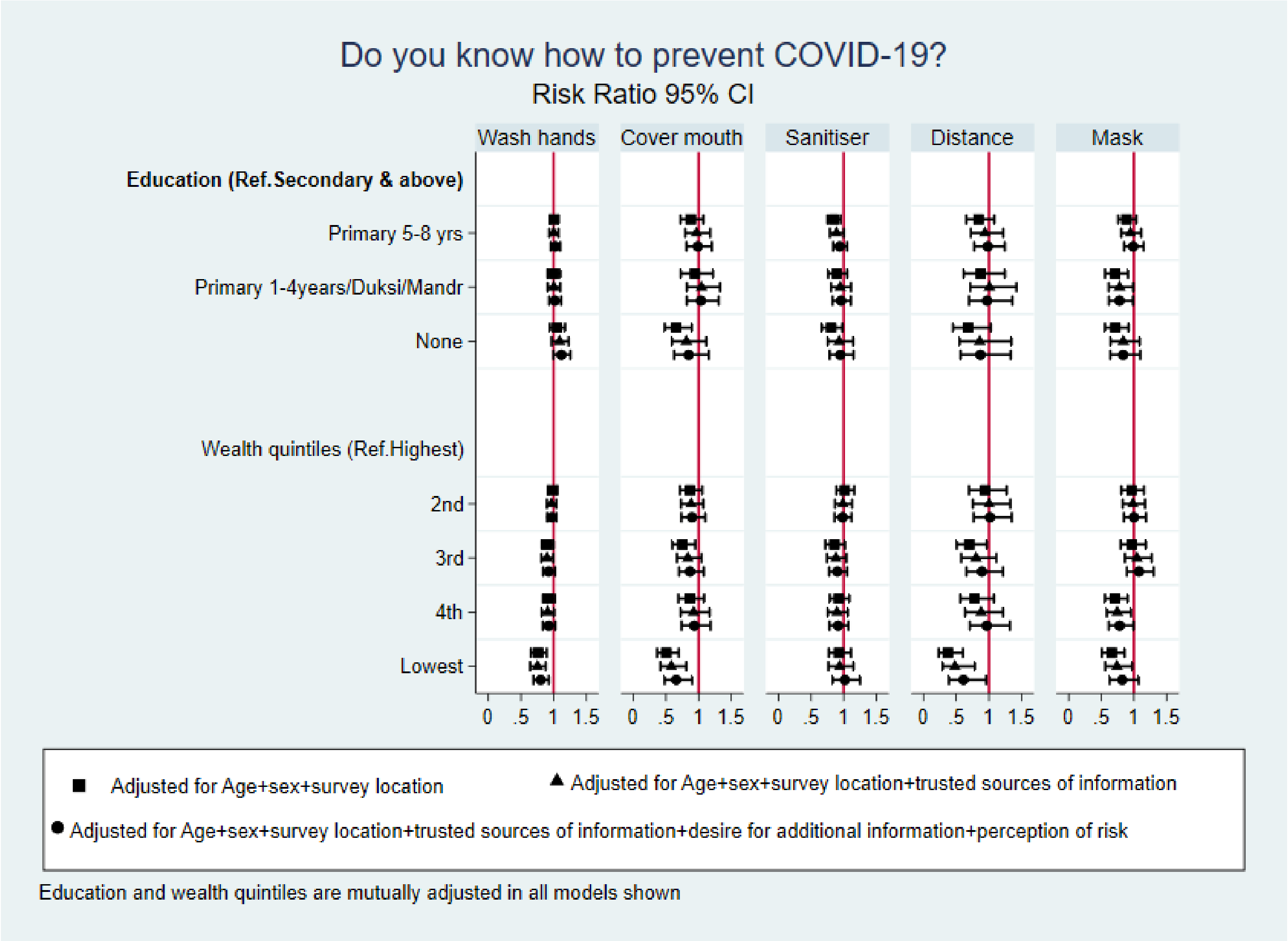

Supplementary Figure 1: Association between knowledge of prevention behaviours and measures of education and wealth. Risk Ratio and 95% confidence intervals.

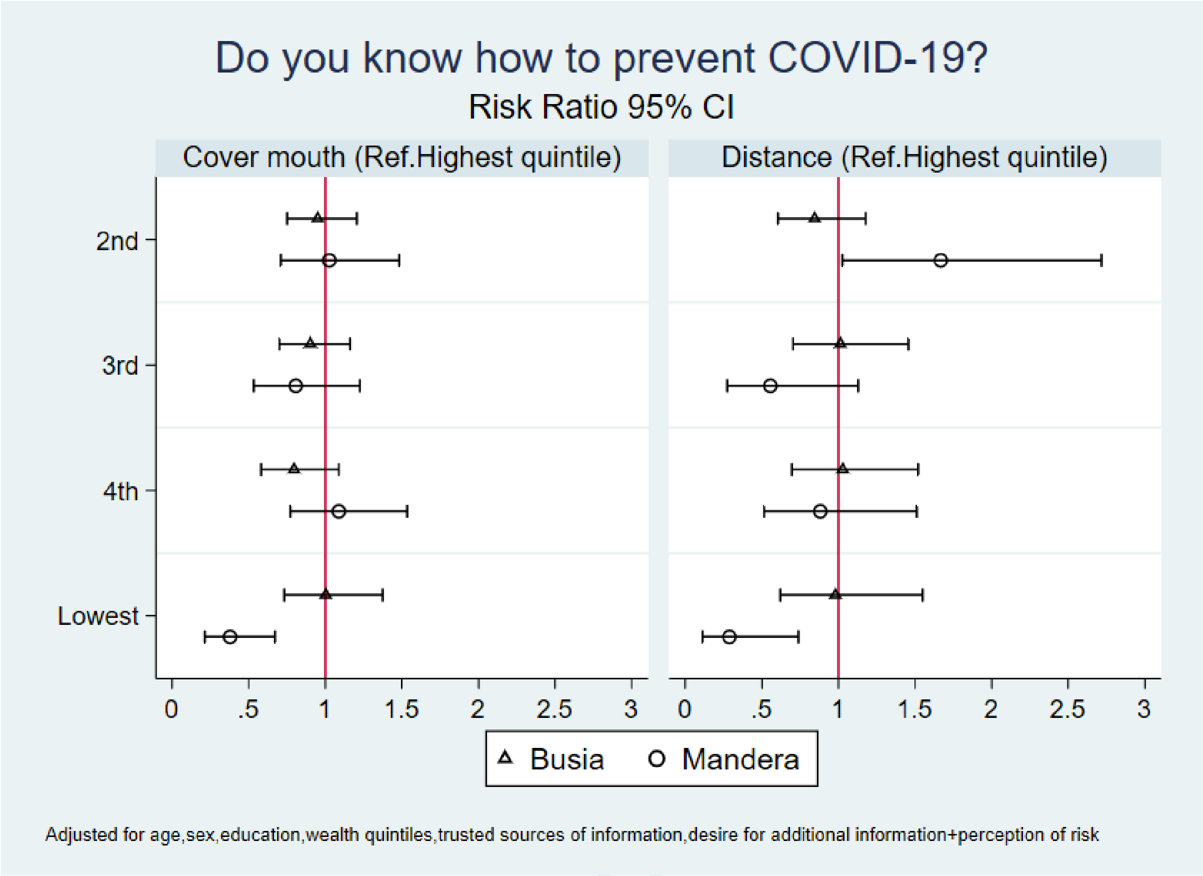

Supplementary Figure 2: Interaction between wealth quintiles and survey area for knowing that covering mouth when coughing/sneezing and keeping 2 metres distance could prevent COVID-19.

STROBE 2007 (v4) checklist of items to be included in reports of observational studies in epidemiology*
Checklist for cohort, case-control, and cross-sectional studies (combined)

Section/Topic	Item #	Recommendation	Reported on page #
**Title and abstract**	1	(*a*) Indicate the study's design with a commonly used term in the title or the abstract	1
		(*b*) Provide in the abstract an informative and balanced summary of what was done and what was found	3
**Introduction**			
Background/rationale	2	Explain the scientific background and rationale for the investigation being reported	4
Objectives	3	State specific objectives, including any pre-specified hypotheses	5
**Methods**			
Study design	4	Present key elements of study design early in the paper	5
Setting	5	Describe the setting, locations, and relevant dates, including periods of recruitment, exposure, follow-up, and data collection	5
Participants	6	(*a*) *Cohort study*—Give the eligibility criteria, and the sources and methods of selection of participants. Describe methods of follow-up<br>*Case-control study*—Give the eligibility criteria, and the sources and methods of case ascertainment and control selection. Give the rationale for the choice of cases and controls<br>*Cross-sectional study*—Give the eligibility criteria, and the sources and methods of selection of participants	5, 7
		(*b*) *Cohort study*—For matched studies, give matching criteria and number of exposed and unexposed<br>*Case-control study*—For matched studies, give matching criteria and the number of controls per case	
Variables	7	Clearly define all outcomes, exposures, predictors, potential confounders, and effect modifiers. Give diagnostic criteria, if applicable	6
Data sources/ measurement	8*	For each variable of interest, give sources of data and details of methods of assessment (measurement). Describe comparability of assessment methods if there is more than one group	6
Bias	9	Describe any efforts to address potential sources of bias	6
Study size	10	Explain how the study size was arrived at	5,7
Quantitative variables	11	Explain how quantitative variables were handled in the analyses. If applicable, describe which groupings were chosen and why	6
Statistical methods	12	(*a*) Describe all statistical methods, including those used to control for confounding	7
		(*b*) Describe any methods used to examine subgroups and interactions	7
		(*c*) Explain how missing data were addressed	7
		(*d*) *Cohort study*—If applicable, explain how loss to follow-up was addressed<br>*Case-control study*—If applicable, explain how matching of cases and controls was addressed	7

For peer review only

		Cross-sectional study—If applicable, describe analytical methods taking account of sampling strategy	
		(*e*) Describe any sensitivity analyses	7
**Results**			
Participants	13*	(a) Report numbers of individuals at each stage of study—eg numbers potentially eligible, examined for eligibility, confirmed eligible, included in the study, completing follow-up, and analysed	8
		(b) Give reasons for non-participation at each stage	
		(c) Consider use of a flow diagram	8
Descriptive data	14*	(a) Give characteristics of study participants (eg demographic, clinical, social) and information on exposures and potential confounders	10
		(b) Indicate number of participants with missing data for each variable of interest	10
		(c) *Cohort study*—Summarise follow-up time (eg, average and total amount)	
Outcome data	15*	*Cohort study*—Report numbers of outcome events or summary measures over time	
		Case-control study—Report numbers in each exposure category, or summary measures of exposure	
		Cross-sectional study—Report numbers of outcome events or summary measures	10
Main results	16	(*a*) Give unadjusted estimates and, if applicable, confounder-adjusted estimates and their precision (eg, 95% confidence interval). Make clear which confounders were adjusted for and why they were included	10
		(*b*) Report category boundaries when continuous variables were categorized	10
		(*c*) If relevant, consider translating estimates of relative risk into absolute risk for a meaningful time period	
Other analyses	17	Report other analyses done—eg analyses of subgroups and interactions, and sensitivity analyses	11-12
**Discussion**			
Key results	18	Summarise key results with reference to study objectives	15
Limitations	19	Discuss limitations of the study, taking into account sources of potential bias or imprecision. Discuss both direction and magnitude of any potential bias	4
Interpretation	20	Give a cautious overall interpretation of results considering objectives, limitations, multiplicity of analyses, results from similar studies, and other relevant evidence	15-17
Generalisability	21	Discuss the generalisability (external validity) of the study results	15-17
**Other information**			
Funding	22	Give the source of funding and the role of the funders for the present study and, if applicable, for the original study on which the present article is based	18

*Give information separately for cases and controls in case-control studies and, if applicable, for exposed and unexposed groups in cohort and cross-sectional studies.

Note: An Explanation and Elaboration article discusses each checklist item and gives methodological background and published examples of transparent reporting. The STROBE checklist is best used in conjunction with this article (freely available on the Web sites of PLoS Medicine at http://www.plosmedicine.org/, Annals of Internal Medicine at http://www.annals.org/, and Epidemiology at http://www.epidem.com/). Information on the STROBE Initiative is available at www.strobe-statement.org.

For peer review only

BMJ Open

Contextually appropriate communication strategies for COVID-19 prevention in Kenya border regions: Evidence from a mixed methods observational study in Busia and Mandera Counties.

Journal:	*BMJ Open*
Manuscript ID	bmjopen-2022-062686.R2
Article Type:	Original research
Date Submitted by the Author:	02-May-2023

Complete List of Authors:	Kaduka, Lydia; Kenya Medical Research Institute, Centre for Public Health Research; King's College London Faculty of Life Sciences & Medicine, School of Life Course Science<br>Olale, Joanna; Kenya Medical Research Institute<br>Karamanos, Alexis; King's College London Faculty of Life Sciences and Medicine<br>Mutai, Joseph; Kenya Medical Research Institute<br>Coultas, Clare; King's College London School of Medical Education<br>Ahmed, Ismail; Kenya Medical Research Institute<br>L'Esperance, Veline; King's College London, School of Population Health and Environmental Sciences<br>Read, Ursula; University of Warwick Warwick Medical School<br>Dazzan, Paola; King's College London Institute of Psychiatry Psychology and Neuroscience<br>Cruickshank, John; King's College London Faculty of Life Sciences and Medicine, School of Life-course and Nutritional Sciences<br>Muniu, Erastus; Kenya Medical Research Institute<br>Harding, Seeromanie; King's College London Faculty of Life Sciences and Medicine

**Primary Subject Heading**:	Public health
Secondary Subject Heading:	Epidemiology, Qualitative research, Infectious diseases
Keywords:	COVID-19, EPIDEMIOLOGY, Public health < INFECTIOUS DISEASES, PREVENTIVE MEDICINE

SCHOLARONE™ Manuscripts

I, the Submitting Author has the right to grant and does grant on behalf of all authors of the Work (as defined in the below author licence), an exclusive licence and/or a non-exclusive licence for contributions from authors who are: i) UK Crown employees; ii) where BMJ has agreed a CC-BY licence shall apply, and/or iii) in accordance with the terms applicable for US Federal Government officers or employees acting as part of their official duties; on a worldwide, perpetual, irrevocable, royalty-free basis to BMJ Publishing Group Ltd ("BMJ") its licensees and where the relevant Journal is co-owned by BMJ to the co-owners of the Journal, to publish the Work in this journal and any other BMJ products and to exploit all rights, as set out in our licence.

The Submitting Author accepts and understands that any supply made under these terms is made by BMJ to the Submitting Author unless you are acting as an employee on behalf of your employer or a postgraduate student of an affiliated institution which is paying any applicable article publishing charge ("APC") for Open Access articles. Where the Submitting Author wishes to make the Work available on an Open Access basis (and intends to pay the relevant APC), the terms of reuse of such Open Access shall be governed by a Creative Commons licence – details of these licences and which Creative Commons licence will apply to this Work are set out in our licence referred to above.

Other than as permitted in any relevant BMJ Author's Self Archiving Policies, I confirm this Work has not been accepted for publication elsewhere, is not being considered for publication elsewhere and does not duplicate material already published. I confirm all authors consent to publication of this Work and authorise the granting of this licence.

For peer review only

Contextually Appropriate Communication Strategies for COVID-19 prevention in Kenya Border Regions: Evidence from a mixed methods observational study in Busia and Mandera Counties

Kaduka Lydia, Olale Joanna, Karamanos Alexis, Mutai Joseph, Coultas Clare, Ahmed Ismail, Lesperance Veline, Read Ursula, Dazzan Paola, Cruickshank Kennedy, Muniu Erastus, Harding Seeromanie.

Kaduka Lydia, Centre for Public Health Research, Kenya Medical Research Institute, Nairobi, Kenya.

Olale Joanna, Centre for Clinical Research, Kenya Medical Research Institute, Nairobi, Kenya.

Karamanos Alexis, School of Population Sciences and Health Services Research and School of Life Course Sciences, King's College London, London, UK.

Mutai Joseph, Centre for Public Health Research, Kenya Medical Research Institute, Nairobi, Kenya.

Coultas Clare, School of Education, Communication and Society, King's College London, London, UK.

Ahmed Ismail, Centre for Public Health Research, Kenya Medical Research Institute, Nairobi, Kenya.

Lesperance Veline, School of Population Sciences and Health Services Research and School of Life-Course Sciences, King's College London, London, UK.

Read Ursula, Department of Mental Health and Wellbeing, Warwick Medical School, University of Warwick, Coventry, UK.

Dazzan Paola, Department of Psychological Medicine, Institute of Psychiatry, Psychology and Neuroscience, King's College London, London, UK.

Cruickshank Kennedy, School of Life-course and Nutritional Sciences, Kings College London, London, UK.

Muniu Erastus, Centre for Public Health Research, Kenya Medical Research Institute, Nairobi, Kenya.

For peer review only - http://bmjopen.bmj.com/site/about/guidelines.xhtml

Harding Seeromanie, School of Population Sciences and Health Services Research and School of Life Course Sciences, King's College London, London, UK.

Corresponding author

Kaduka Lydia

Kenya Medical Research Institute

Centre for Public Health Research

P.O. Box 20752-00202, Nairobi, Kenya

Tel: (254)-2-2725016/7. Fax: (254)-2-2720030

Email: lkaduka@kemri.go.ke

Keywords: COVID-19, Border regions, Kenya

Word Count: 4529

For peer review only - http://bmjopen.bmj.com/site/about/guidelines.xhtml

ABSTRACT

Objectives: Kenya has long and porous borders with its neighbouring countries. These regions, predominantly inhabited by highly mobile rural communities with strong cross-border cultural ties, present major challenges in managing movement of people and COVID-19 preventive measures. Our study sought to assess knowledge of COVID-19 prevention behaviours, how these varied by socio-economic (SEC) factors, and the challenges of engagement and implementation, in two border counties of Kenya.

Methods: We conducted a mixed methods study using a household e-survey (Busia N=294; Mandera N=288; 57% females, 43% males), and qualitative telephone interviews (N=73: Busia 55; Mandera 18) with policy actors, healthcare workers, truckers and traders, and community members. Interviews were transcribed, English-translated and analysed using the Framework Method. Associations between SEC (wealth quintiles, educational level) and knowledge of COVID-19 preventive behaviours were explored using Poisson regression.

Results: Participants were mostly educated to primary school level (54.4% Busia, 61.6% Mandera). Knowledge of COVID-19 prevention varied by behaviour: hand washing - 86.5%, use of hand sanitiser -74.8%, wearing a face mask - 63.1%, covering the mouth when sneezing or coughing -56.3% and social distancing- 40.1%. Differences in knowledge by area, educational level and the wealth index were marked, greatest for Mandera, the less educated and the poor. Interviews with stakeholders revealed challenges in health messaging, psychosocial and socioeconomic factors, lack of preparedness for truck border crossings, language barrier, denial and livelihood insecurity as key challenges to engagement with and implementation of COVID-19 prevention behaviours in the border regions.

Conclusion: The influence of SEC disparities and border dynamics on knowledge and engagement with COVID-19 prevention behaviours call for contextually appropriate risk communication strategies that are cognisant of community needs and local patterns of information flow. Coordinating response measures across border points is crucial in winning communities' trust and maintaining essential economic and social activities.

For peer review only - http://bmjopen.bmj.com/site/about/guidelines.xhtml

STRENGTHS AND LIMITATIONS

- The study sampling strategy and high e-survey response rate (97%) were key strengths, which point to the versatility required in the conduct of research in emergency response with community engagement being central to the success of similar research projects.
- The study did not objectively assess the knowledge of preventive behaviours. To overcome the potential bias of self-reported information, qualitative interviews were used to explore factors influencing the ability of the community to adhere to preventive measures.
- Whereas Kenya's median age is 21, majority of the e-survey participants were $\geq$55 years. This group tends to be less tech savvy and may explain the trust hierarchy observed in this study.
- The study recorded a lower number of Mandera interviews. However, the context-specific challenges presented what may be considered socio-culturally appropriate recommendations to inform future local efforts.

INTRODUCTION

Evidence continues to accrue on the dramatic global direct and indirect impacts of the COVID-19 pandemic. The pandemic has put a toll on both the social and economic structures of societies, contributing to global social, economic and health disparities [1-3]. Kenya has a population of ≈48 million, median age 20.1 years and 66.2% living on <US $3.20 /day [4]. It has long and porous borders with Tanzania, Uganda, Ethiopia, South Sudan, and Somalia, predominantly inhabited by highly mobile rural communities with strong cross-border cultural ties. Eight weeks after the first reported COVID-19 case in Kenya on March 13th 2020, a quarter of all 781 reported cases came from these border counties [1]. A single-wave epidemic peaked within 100 to 200 days after the first reported case [3]. Recognising its vulnerability to COVID-19 due to its position as a major travel and transport hub and relatively weak health system, Kenya implemented several mitigation measures including: a dusk to dawn curfew; mandatory mask wearing; prohibition of large public gatherings; and closure of borders [5,6]. Indirect impacts of the measures resulted in increased poverty levels, disrupted livelihoods, deepened inequalities and decelerated progress towards the Sustainable Development Goals [7-9].

The needs of communities at border regions are an important consideration for Kenya and its neighbours due to their shared vision of regional health security. The porous borders in

For peer review only - http://bmjopen.bmj.com/site/about/guidelines.xhtml

the Mandera triangle (Kenya, Ethiopia, and Somalia borders) and Busia (Kenya -Uganda border) limits the ability of partner states to monitor and identify active cases during pandemics [10-15]. With only 18% of the Kenyan population fully vaccinated by January 19th 2022 [16], the mainstay of the response remained adoption of preventive behaviours to reduce transmission.

In this study, we aimed to assess the levels of knowledge of key COVID-19 prevention behaviours, and whether these varied by socioeconomic factors, and obtained qualitative narratives to illuminate contextual factors impacting on engagement with prevention measures in Busia and Mandera border counties of Kenya. These two border counties continue to record poorer health outcomes due to long-standing issues in accessing reasonable quality healthcare because of poor health infrastructure, remoteness from the capital city, and high poverty rates. They are also among the eight lowest ranked counties in Kenya with the least healthcare workers per capita [17].

METHODS

Study design

A convergent parallel mixed methods study design was used to collect both quantitative and qualitative data in the two counties between September and October 2020. The design allows concurrent collection and analysis of both data sets, and convergence at interpretation stage [18].

Study settings: Mandera County located in Northern Kenya shares a border of 861km with Ethiopia to the North and 682 km with Somalia to the East (Figure 1). It is located 1052km from Kenya's capital city Nairobi, covers an area of 25,798 km², has a population of 867,457, population density of 33 persons/km², high poverty level (77.6%), and is inhabited predominantly by Muslim Somalis. Mandera's main economic activities are pastoralism and cross-border trade. Of Mandera's 775,085 population aged $\geq$3 years, 72% have never been to a learning institution. Busia County located 475km from Nairobi, borders Lake Victoria to the North and Uganda to the West (total border distance, 772 Km). With a 1,700Km² area and population of 893,000, population density is 527 persons/km², and poverty level 69%. Its main economic activities are cross-border trade, fishing, and agriculture, and is inhabited predominantly by Christians. 12.5% of Busia's 820,788 population aged $\geq$3 years have never been to school [19].

Quantitative survey

For peer review only - http://bmjopen.bmj.com/site/about/guidelines.xhtml

Sample size and sampling procedure: A cross-sectional household e-survey targeting adults aged ≥18 years was undertaken in September 2020. Sample size calculation was based on a prevalence of 50% for the indicator of interest (knowledge) and a maximum relative standard error for the estimated proportion of 7% to give a sample size of 205. Assuming 20 participants per cluster (communities), an intra-class correlation coefficient between clusters of 0.01, and a non-response rate of 10%, the resulting sample size was 269 rounded off to 300 participants, equivalent to 15 clusters per county. A three-stage sampling process was used. "The first stage involved listing of all public health facilities in the study areas (Busia 66; Mandera 79) and the square root allocation method used to determine the health facilities sampled per area. Fifteen facilities per area were selected using systematic sampling with a random start. For each selected health facility, one community health unit (CHU) served by the health facility was selected using simple random sampling. Community health workers (CHWs) in selected CHUs assisted in developing the household sampling frame. The second stage involved sampling 20 households within CHUs using systematic sampling with a random start. The third stage involved respondent selection in sampled households using the Kish Grid method [20] as a way of balancing representation".

Data collection and analysis: The e-survey data was collected using the WHO COVID-19 Rapid Quantitative Assessment Tool (Supplementary File 1) adapted to the local context [21]. The study used the REDCap software [22] to automate and share the survey tool with study participants. The e-questionnaire was administered either electronically on a smartphone or via telephone interview for participants with literacy or technology challenges. The study worked with trained CHWs in verifying respondent's eligibility and access to a smart phone with internet connection. A REDCap link was shared which allowed the respondent to consent, fill and submit the e-survey. Due to erratic internet in the study sites, the participants were allowed to submit their survey forms any time within the day using a unique return code generated specific to each participant to safeguard completion. Technical support was offered via WhatsApp.

Outcome measures: Knowledge about COVID-19 related preventive health behaviours was assessed by asking survey participants the following question *"Do you know how to prevent COVID-19?"*, to which they responded with a yes/no on the following individual statements;

For peer review only - http://bmjopen.bmj.com/site/about/guidelines.xhtml

prevent COVID-19 by (i) wearing a face mask, (ii) keeping a distance of 2 metres from others, (iii) covering the mouth and nose when sneezing or coughing, (iv) using a hand sanitiser, and (v) washing hands with soap and water regularly. Affirmative responses were categorised as the reference category. Exposure status was assessed by asking participants if they had experienced any of the following symptoms - fever, cough, shortness of breath, sore throat, fatigue, loss of taste or smell, or eye infection, two weeks prior to the survey.

Correlates and confounders: Socio-economic circumstances (SEC) were measured using education and the wealth index, two commonly used SEC indices in Kenya [19]. The wealth index was calculated using data on household's ownership of 14 selected assets (Supplementary Table 1). The weights (factor scores) for each of the assets were generated through principal components analysis (PCA). Years of education and adult education were categorised as following; 1) Secondary education and above (more than 9 years of compulsory years of education), 2) upper primary education (5-8 years of compulsory education), 3) lower primary education (1-4 years of compulsory education) or/and Islamic education (Duksi or Madrassa), and 4) no education.

Participant's sex, age (categorised in 4 age bands 18-24, 25-54, 55-65 and 65+) and survey location (Mandera/Busia) were considered as potential confounders. Trusted sources of information, desire for additional information on COVID-19 and perception of risks stemming from SARS CoV2 were considered as potential mediators and were assessed by asking participants to respond with a yes/no to each category for the following questions: *"Which source/who do you trust the most to receive information related to the new Coronavirus?" with response categories* (i) the radio, (ii) the TV, (iii) the WhatsApp messenger, (iv) Community Health Workers (CHWs) and (v) religious leaders. Desire for additional information on COVID-19 was measured using the question: *"What more would you like to know about COVID-19?"* with response categories (i) Symptoms of the new Coronavirus, (ii) How it is transmitted, (iii) what to do if you have symptoms; Perceived risks from COVID-19 (i) *"How dangerous do you find the new Coronavirus?"*, (ii) *"Do you think you are likely to become sick with the new Coronavirus"*, and (iii) "*Do you think any of your family members are at risk of becoming sick from the new Coronavirus?"*. Responses to the first question were categorised as very dangerous, dangerous

For peer review only - http://bmjopen.bmj.com/site/about/guidelines.xhtml

or “not at all”. The latter two were combined due to small numbers reporting ‘not at all’. Response to the other two questions consisted of yes, no and do not know. Information on gender, survey location, and age group were also collected.

Statistical analyses: All statistical analyses used the Stata V.16 software [23] and the statistical significance level set at 5%. The association between the exposures (wealth quintiles and educational level) and the outcomes of interest (knowing that COVID-19 can be prevented by specific behaviours) was explored using Poisson regression [24]. Poisson regression was preferred over logistic regression to allow relative risks to be presented, rather than odds ratios which are often misrepresented. All regression analyses applied survey weights to account for the higher proportion of women included in the survey (57% vs. 43% for males) and the higher proportion of survey participants with secondary education and above (28.5% vs. 23.7% for those with no education). The multivariable regression analysis adjusted for wealth quintiles, educational level, gender, survey location, age group, trusted sources of information about COVID-19, desired additional information on COVID-19 and perception of risks.

Qualitative interviews

Sampling procedure: Purposive sampling was used to identify and select respondents with professional or personal experience of COVID-19. Semi-structured, in-depth telephone interviews were done with policy makers, healthcare workers, truckers, traders and COVID-19 survivors and carers to assess their experiences and needs in relation to the pandemic and mitigation measures specific to border counties’ populations. Interviewees were mapped using an initial stakeholders list which included the counties’ leadership, health management teams, and the COVID-19 response teams, to which further additions were made through snowballing. Interviews were completed between September and October 2020.

***Data collection and analysis*:** Key informant interviews were carried out to explore understanding and experiences of the pandemic (Supplementary File 2). The interviews were conducted in either Kiswahili or English based on preference of the interviewees, time range of 30-60 minutes per interview. Questions related to the following topics were explored: community response to, and experience of the pandemic; health systems’ preparedness and response to the pandemic; and border-specific issues related to the pandemic (only data

For peer review only - http://bmjopen.bmj.com/site/about/guidelines.xhtml

pertaining to prevention behaviors are presented in this paper). The recorded interviews were transcribed, translated, and checked for quality by the Kenyan research team, and a UK-based co-author (CC) who is fluent in Swahili. The transcripts were then analysed using the framework method (Gale et al. (2013) [25]: open-coding of the data was undertaken in pairs or small teams (with at least one Kenyan, and one UK-based individual) so that sociocultural differences in interpretations of the data could be identified and engaged with. The team then met altogether to develop an analytical framework with key categories (i.e. groupings of codes that are clearly defined). The analytical framework was then applied to all of the transcripts. Illustrative quotes were charted into a framework matrix and the data was interpreted and organized into themes.

Ethical considerations: Informed consent was sought and obtained from participants prior to their participation. E-consenting was done on REDCap, which involved mandatorily displaying the informed consent document. At the end of the consent document, the participant would click a mandatory check box in which they either provided or withdrew consent. The survey was only accessible upon consent. The digital record that the box was checked was archived as evidence of consent.

Patient and Public Involvement: The research question was arrived at upon interrogating the existing covid-19 response measures and realising a potential gap in addressing the needs of border communities. The County administration assessed the relevance of the proposed research and gave permission for community participation in the study. Community health workers and village elders assisted in sensitising and mobilising community participation, participated in mapping and selection of households, and sampling of study participants. The study findings were discussed in stakeholder forums with representation from the counties, community-based organisations and members of health committees of the local legislature. Recommendation items actionable at county level were taken up by respective county teams for implementation.

RESULTS

Knowledge of key COVID-19 prevention behaviours in Busia and Mandera

We assessed levels of knowledge of key COVID-19 prevention behaviours, and whether these varied by socioeconomic factors. Table 1 shows the sample profiles for the 582 adults (Busia: 50.5%; Mandera 49.5%) who participated in the e-survey.

For peer review only - http://bmjopen.bmj.com/site/about/guidelines.xhtml

Table 1: Sample profiles in Busia and Mandera Counties

	Busia (N=294)	Mandera (N=288)	All (N=582)
	**Proportion (95% CI)**	**Proportion (95% CI)**	**Proportion (95% CI)**
**Age group**			
18-24 (Early working age)	8.6 (5.6 to 12 .9) §	1.4 (0.6 to 3.5)	5.0 (3.4 to 7.3)
25-54 (Prime working age)	6.8 (4.3 to 10.6)	7.2 (4.7 to 10.8)	7.0 (5.1 to 9.5)
55-64 (Mature working age)	59.9 (52.8 to 66.7)	52.1 (45 to 59.1)	56.0 (51 to 60.9)
65 plus (Elderly)	24.7 (18.5 to 32) §	39.3 (32.3 to 46.8)	32 (27.2 to 37.3)
**Education level**			
Secondary and above	40.9 (34.4 to 47.6) §	15.1 (11.4 to 19.9)	28.0 (24.0 to 32.3)
Primary 5-8 yrs	28.6 (23.6 to 34.1) §	3.4 (2 to 5.8)	16.0 (13.4 to 19.0)
Primary 1-4years/Duksi/Madrasa	25.8 (19.1 to 34) §	58.2 (51.6 to 64.5)	42.0 (36.9 to 47.3)
None	4.7 (3.0 to 7.4) §	23.3 (18.9 to 28.3)	14.0 (11.6 to 16.8)
**Wealth quintiles**			
Highest quintile	16.9 (2.5 to 12.6) §	23.2 (17.8 to 29.7)	20.1 (16.5 to 24.2)
2nd	21.5 (2.7 to 16.7) §	12.7 (9.1 to 17.4)	17.1 (14.0 to 20.7)
3rd	29.7 (3.4 to 23.4) §	16.3 (11.6 to 22.4)	23.0 (18.9 to 27.7)
4th	17.8 (2.7 to 13.1) §	18.4 (14 to 23.9)	18.1 (14.8 to 22.0)
Lowest	14 (2.4 to 10) §	29.4 (23.1 to 36.6)	21.7 (17.8 to 26.2)
**Preventative behaviours**			
Wash hands regularly with soap and water	95.9 (92.3 to 97.9) §	77 (70.4 to 82.5)	86.5 (82.6 to 89.6)
Cover mouth and nose when coughing or sneezing	64.3 (57.3 to 70.7) §	48.3 (41.3 to 55.3)	56.3 (51.4 to 61.1)
Hand sanitiser use	84.4 (78.7 to 88.9) §	65.2 (58.3 to 71.6)	74.8 (70.3 to 78.9)
Standing 2 meters away from other people	52.2 (45.3 to 59.1) §	27.9 (22.2 to 34.6)	40.1 (35.4 to 45.0)
Wear a face mask	70.9 (64.2 to 76.9) §	55.3 (48.2 to 62.3)	63.1 (58.2 to 67.9)
**Trusted source of information on COVID-19**			
Radio	92.8 (88.6 to 95.5) §	54.8 (47.8 to 61.7)	73.8 (69.4 to 77.8)
TV	51.6 (44.7 to 58.4) §	11.8 (7.9 to 17.4)	31.7 (27.3 to 36.4)
WhatsApp	25.2 (19.8 to 31.5)	19.1 (14.2 to 25.3)	22.2 (18.4 to 26.5)
Community Health Workers	56.4 (49.4 to 63.1) §	43.7 (37.0 to 50.7)	50 (45.1 to 55)
Religious leaders	33.3 (27.3 to 39.9) §	46.2 (39.3 to 53.3)	39.7 (35 to 44.6)
**Kind of information received**			
Symptoms of COVID-19	82.3 (76.5 to 86.9) §	60.3 (53.2 to 66.9)	71.3 (66.6 to 75.5)
How SARS CoV2 is transmitted	74 (67.5 to 79.5) §	40.9 (34.2 to 47.9)	57.4 (52.5 to 62.2)

For peer review only - http://bmjopen.bmj.com/site/about/guidelines.xhtml

	Busia (N=294)	Mandera (N=288)	All (N=582)
	**Proportion (95% CI)**	**Proportion (95% CI)**	**Proportion (95% CI)**
What to do if you have symptoms	65.7 (58.9 to 72) §	28.7 (22.7 to 35.6)	47.2 (42.3 to 52.2)
**Perception of risk**			
**How dangerous COVID-19 is?**			
Very dangerous	89.3 (84.4 to 92.8) §	73.2 (66.7 to 78.9)	81.3 (77.2 to 84.8)
Less dangerous/Not dangerous	10.7 (7.2 to 15.6) §	26.8 (21.1 to 33.3)	18.7 (15.2 to 22.8)
**Do you think any of your family member are at higher risk of becoming sick?**			
Yes	53.3 (46.4 to 60)§	20.4 (15.6 to 26.2)	36.8 (32.3 to 41.7)
No	13.8 (9.8 to 19.2)	13.8 (9.9 to 18.9)	13.8 (10.9 to 17.4)
Don't know	32.9 (26.8 to 39.6)§	65.8 (59.1 to 71.9)	49.4 (44.4 to 54.3)
**Do you think you are likely to become sick with COVID-19?**			
Yes	56.1 (49.2 to 62.8)§	16.3 (12.2 to 21.4)	36.2 (31.6 to 41)
No	14 (9.9 to 19.3)	16.8 (12.3 to 22.6)	15.4 (12.2 to 19.2)
Don't know	29.9 (24.1 to 36.6)§	66.9 (60.3 to 72.9)	48.4 (43.5 to 53.4)
COVID-19 symptoms			
Fever	12.9 (9 to 18.1)	20.2 (15.6 to 25.8)	16.5 (13.4 to 20.2)
Cough	12.5 (8.6 to 17.7)	21.1 (16.3 to 26.9)	16.8 (13.6 to 20.6)
Shortness of breath	7.7 (4.6 to 12.5)	9.9 (6.6 to 14.6)	8.8 (6.4 to 12)
Sore throat	7.1 (4.3 to 11.7)	6.4 (3.8 to 10.3)	6.7 (4.7 to 9.6)
Fatigue	5.2 (2.8 to 9.5)	4.5 (2.5 to 9.1)	4.9 (3.1 to 7.4)
Loss of taste or smell	5.9 (3.2 to 10.7)	2.1 (0.9 to 5)	4 (2.4 to 6.6)

§ 95% CIs do not overlap

Majority (95%) of the participants were 25 years and above, 14% lacked formal education. Socio-economic disadvantage was high in both locations, with just over a quarter in Busia and over half of those in Mandera reporting having received only Primary 1-4 years, Duksi or Madrasa education. Mandera was consistently more disadvantaged than Busia across all measures. Overall, 17% reported a fever and cough, while 4% reported loss of smell/taste two weeks before the survey.

In both areas, the most known common prevention behaviours identified were washing hands regularly 86.5% (Busia 95.9%, 95% CI 92.3-97.9; Mandera 77%, 70.4-82.5) and use of a hand sanitiser 74.8% (Busia 84.4%, CI 78.7-88.9; Mandera 65.2%, CI 58.3-71.6) and the least commonly

For peer review only - http://bmjopen.bmj.com/site/about/guidelines.xhtml

known was standing two metres away from others 40.1% (Busia 52%, 45.3-59.1; Mandera 28%, 22.2-34.6). Significantly lower proportions in Mandera than in Busia reported knowledge of prevention behaviours, having had secondary education, being in the most disadvantaged 4th and 5th wealth quintiles, having trust in COVID-19 related information from various sources including the radio and TV, and having received COVID-19 related knowledge on symptoms, transmission and what to do if they have symptoms. There were some notable differences for trusted sources of COVID-19 information. In Busia, 93% cited the radio as the most trusted source, whereas in Mandera the radio (55%), community health workers (44%) and religious leaders (46%) shared the top ranking for trusted source. Table 2 shows the influence of adjusting for SEC, trusted sources of information, additional information and perception of risk on the Mandera-Busia differences in knowledge of prevention behaviours.

Table 2: The influence of adjusting for socio-economic circumstances, trusted sources of COVID-19 information, and additional information and perception of risk on the Mandera-Busia differences in knowledge of prevention behaviours. Risk Ratio (95% confidence interval), Busia is the reference category.

	Mandera-Busia difference in prevention behaviour, adjusted for gender and age	+ education level and wealth quintiles	+ trusted sources of information about COVID-19 knowledge	+desired additional information on COVID-19, and perception of risk
Wash hands regularly with soap and water	0.80 (0.74 to 0.87)***	0.81 (0.74 to 0.89)***	0.81 (0.73 to 0.90)***	0.86 (0.78 to 0.96)**
Cover mouth and nose when coughing or sneezing	0.72 (0.61 to 0.87)***	0.81 (0.64 to 1.03)	0.87 (0.67 to 1.14)	0.99 (0.76 to 1.28)
Hand sanitiser use	0.77 (0.68 to 0.86)***	0.80 (0.69 to 0.92)**	0.88 (0.75 to 1.02)	0.99 (0.85 to 1.16)
Standing 2 meters away from other people	0.52 (0.40 to 0.68)***	0.61 (0.44 to 0.85)**	0.58 (0.40 to 0.84)**	0.71 (0.50 to 1.01)+
Wear a face mask	0.77 (0.66 to 0.90)**	0.97 (0.79 to 1.20)	0.93 (0.73 to 1.17)	1.06 (0.83 to 1.36)

*+ p <0.1 and >0.05, * p<0.05, ** p<0.01, *** p<0.001*

For peer review only - http://bmjopen.bmj.com/site/about/guidelines.xhtml

The lower knowledge of washing hands in Mandera compared with Busia remained after fully adjusting, with hardly any shift in the risk ratios. The differences between Mandera and Busia in knowledge about covering the mouth when coughing/sneezing and wearing a mask were no longer significant on adjustment for SEC, for using a sanitiser on adjustment for trusted sources of COVID information, and for keeping a 2 metres distance from others on adjustment for all confounders.

Based on the pooled Busia and Mandera sample and after adjusting for confounders including location, the knowledge of prevention behaviours did not vary by education (Supplementary Figure 1). In contrast, regardless of location those in the most disadvantaged 5th wealth quintile remained less likely to report knowledge of three of the five prevention behaviours – washing hands regularly (- 20%, 95% CI -7 to -31%), covering mouth and nose when sneezing or coughing (-34%, -10 to -53%) and standing two metres away from others (-39%, -4 to -61%).

Significant interactions were observed between quintiles of wealth and location for knowing that covering the mouth when coughing or sneezing ($F=35.23$, $p<0.001$) and keeping 2 metres distance ($F=13.26$, $p=0.009$) could prevent COVID-19. The most socio-economically disadvantaged participants in Mandera were 62% (95% CI 33%-79%) less likely to know that COVID-19 could be prevented by covering their mouth while coughing/sneezing, and 61% (26% - 89%) less likely to know that COVID-19 could be prevented by keeping 2 metres distant (See Supplementary Figure 2). No significant differences in the knowledge of this behaviours were observed between the least and the most socioeconomically disadvantaged in Busia.

Qualitative insights into factors impacting on engagement with prevention and control measures

A total of 73 qualitative interviews (Busia: n= 55; Mandera: n= 18) were carried out with policy actors (n=8), healthcare workers (n=34), truckers and traders (n=7), COVID-19 survivors and carers (n=8) and other community members including religious leaders, village elders, persons living with disability, and commercial sex workers (n=16) in the two counties. Age of interviewees ranged between 23-74 years.

The qualitative interviews provided insights into contextual factors which impacted on engagement with prevention and control measures. Table 3 provides a summary, including

For peer review only - http://bmjopen.bmj.com/site/about/guidelines.xhtml

quotes from the qualitative interviews, to illustrate factors impacting on engagement with prevention measures in the two border regions. Lack of trust in the national government and political leaders was noted as a key barrier to engagement with prevention measures in the two border regions. Participants reported that this lack of trust mainly stemmed from alleged misappropriations of funds and politicians not adhering to COVID-19 restrictions.

Other reported impacts included socioeconomic factors such as the loss of livelihoods (especially in the informal sector), and increased cost of living due to rising food prices occasioned by disruptions in local trade and food imports, which impacted COVID-19 prevention measures. Psychosocial factors such as the centrality of spiritual support and family social connections in communities' cultures and way of life conflicted with preventive measures.

Additionally, there was reported to be inadequate attention to providing targeted, context specific, accessible risk communication and public health advice which impacted on equitable understanding and adherence. Challenges included use of technical global pandemic language and procedures that were not translatable to the local setting; lack of community engagement in tailoring communication messages; lack of targeted messaging for the pastoralist community in Mandera; language barriers with French-speaking truckers from Congo and Rwanda; and misinformation within the community regarding COVID-19 symptoms and transmission. Some initiatives by the two county governments were reported to facilitate engagement with prevention, including informal provisions of hand washing stations at the border posts, markets, shops, and religious institutions.

Challenges specific to border areas included, lack of preparedness for truck border crossings which led to pooling of truckers at the border especially in Busia, thus risking community transmission. Cross-border collaboration in enforcing the preventive measures was enhanced through leveraging an already existing infectious disease surveillance network in Mandera but challenged by stigmatization of truckers in Busia.

For peer review only - http://bmjopen.bmj.com/site/about/guidelines.xhtml

Table 3: Qualitative Insights on impacts on adherence to prevention measures
* (B/M) indicates location (Busia/Mandera), (M/F) indicates gender (male/female).

THEMES	CATEGORIES	ILLUSTRATIVE QUOTES
**Distrust of national government and politicians**	Politicians not adhering to COVID restrictions	BM-Imam-118: "They look at the politicians and how they are conducting themselves. Those breaking the rules do so because those that are supposed to encourage them are not adhering to the rules"<br>BM-Comm.HealthVolunteer-018: "people see the leaders walking without wearing masks [… so] they believe there is no corona"<br>MM-Trader-074 "we see politicians doing things without social distance… it only means there is no COVID"
	Media coverage of misappropriation of COVID funds	BM-Comm.HealthVolunteer-003: "having heard that there is a lot of money that was squandered through Kenya Medical Supplies Authority… majority of them are not doing the right thing [i.e. taking preventative measures]"<br>BM-PublicHealthOfficer-052: "people started relaxing in adhering after this scandal was unearthed whereby, they say that billions of money was stolen"
**Health messaging not adequately contextualised to local communities**	Insufficient attention to impact of isolation on livelihoods	BM-COVIDSurvivor-111: "people fear to go and test, because they know you will just dump somebody there [in an isolation centre] for ten days […] you don't know the person, the way they eat, survive, he is just there like that in the name of quarantine, isolation, all those things"
	Inadequate community engagement and tailored public health messaging	BM-Policyactor-087: "we need to have more community engagement, with interpersonal communication kind of approach, so that you do more than just general messages, you are doing specific messages to specific groups of people, truck drivers, sex workers, maybe clearing agents and so on"<br>BM-Policyactor-113: "Communication on protection was not well done. We needed more of community engagement and good communication to ensure that all issues are clarified through question and answers"<br>BM-Policyactor-096: "remember the kind of information which was being given out initially [… led] by the Ministry of Health was more of .. well it was a caution but it really sounded to the public like this thing is going to wipe away so many people […] the words were really confusing, quarantine, isolation and so on"
	Circulation of misinformation in the community	- 'COVID is just like malaria, it should not be feared' (BM-Farmer-085; BF-Comm.HealthVolunteer-004).<br>'COVID only affects people in big cities and truckdrivers' / 'people do not know people who have had COVID - seeing is believing' (BF-Policyactor-025; BM-CommercialSexWorker-068; MM-Policyactor-007).
	Difficulties engaging with pastoralist communities (in Mandera)	MM-Policyactor-112: "the pastoralist nature of our society, you know people moving from here to the other side [of the border], uncontrollable has also been a serious issue [… they get health information from listening to] vernacular radio stations […] [but] compliance is another thing"<br>MM-Policyactor-007: "we had cases that were imported from Somalia by people who went to sell their livestock [and crossed the border unchecked]"
**Psychosocial and socioeconomic impacts on**	Desire for spiritual and social connection	BM-Policyactor-087: "people are social they want to be together but now they have been told to keep a distance… And when churches were closed, this was very strange… you will find it is affecting people spiritually"<br>BM-Policyactor-087: ""where people used to greet you by shaking hands, and now people are supposed to learn a new mode of greeting which is not our culture and to kick Kenyan culture is not so easy"

For peer review only - http://bmjopen.bmj.com/site/about/guidelines.xhtml

**preventive measures**		MF-COVIDSurvivor-098: "To protect yourself from corona, you are supposed to wear masks, don't greet people and also restrain from hugging people but sometimes you forget and greet people using your hands, you hug people because you are used to this"
	Socioeconomic barriers	BM-PLWDisability-117: "where will we disabled people get masks from? […] We have to work hard and buy for ourselves from our own pockets, buy for the children"<br>MM-Healthworker-037: "there's nobody who can provide them with masks, they were buying at […] twenty shillings or fifty shillings, and so it was very expensive?'"<br>MM-Policyactor-112: "There are these small [informal] businesses [e.g. tea/coffee stands], and… when we want to enforce those [social distancing] regulations it becomes very difficult because this is somebody's daily bread".
	Religious and social spaces supporting adherence	MM-Trader-074: "Even at the shop I have sanitiser, running water with soap, so people when they come to the shop they sanitize".<br>MF-COVIDSurvivor-098: "We have [hand]washing stations in hospitals, madrasa, at the mosque"<br>MM-PublicHealthOfficer-039 "we had a meeting with all the religious leaders… [to discuss how some preventive] measures… So after sensitizing, at least people have started accepting [preventive measures], because it was a big challenge for people to accept social distance in the place of worship"
**Issues specific to border areas**	Lack of preparedness for truck border crossings	BM-Policyactor-096: "initially we had lots of challenges... the trucks piled up… [resulting] in drivers not being able to cross over to Uganda on time, they ended up mingling with the rest of the population and so we knew it was just a matter of time before we got the first community cases"<br>BM-PublicHealthOfficer-052: ""you find this community transmission tends to be growing [because truckdrivers must wait for 2-3 days for the COVID test results]… we feel that there is a need for testing to be done across the entire [border] township […] but we lack the resources for doing this"
	Health prevention information not translated into all needed languages	BM-Policyactor-087: "people from Congo, from Rwanda, they speak French and […] most of our communications are in Kiswahili and English and therefore—like now we don't have any material for French here at the border and that is a huge challenge"
	Lack of consistency in cross-border communication	MM-Policyactor-007: "previous cooperation about polio issues […] can also help in responding to this pandemic"<br>BM-PublicHealthOfficer-052: "It is compulsory in Kenya to put on masks…these drivers when in Uganda they are not allowed to mingle with people, they think that anybody coming from Kenya is having Corona [because of the mask wearing]"<br>BM-Policyactor-113: "Kenya and Uganda might not have been reading from the same script. The countries should have the same lab where they collect the samples and the same approach to the communities. I believe they have community health issues at the border points [like us]. In times of a pandemic, no one is to blame. We have our own challenges but those that spread across East Africa would be dealt better with collaboration"
	Disruptions to cross-border trade and food security	BM-TraditionalHealer-072: "you can't cross the border to go to the Ugandan side [of the forest to collect herbs], if you are found, maybe you passed sideways, you get arrested. We had to stop working"<br>BM-Comm.HealthVolunteer-003: "there is food insecurity, people used to do farming freely, do business, they are unable to cross the border"

For peer review only - http://bmjopen.bmj.com/site/about/guidelines.xhtml

DISCUSSION

We assessed the levels of knowledge of key COVID-19 prevention behaviours in the two border counties. The best-known preventive behaviour was washing of hands regularly and the least known was standing two metres apart, with Mandera significantly disadvantaged. We observed disparate knowledge of COVID-19 prevention measures based on socioeconomic status. Those with less education and those in lower socioeconomic groups were less likely to adhere to COVID-19 prevention measures. Key factors impacting on engagement with COVID-19 prevention measures included lack of trust in the leadership, deficits in public health communication, livelihood insecurity, prioritization of social support over quarantine/social distancing, and border-specific complexities such as language barriers and congregating of truckers due to lack of preparedness for truck border crossings.

Influence of socioeconomic inequalities on knowledge of COVID-19 prevention behaviours

Our study findings are consistent with previous studies in Kenya and other parts of Africa that observed variations in knowledge of COVID-19 prevention measures based on employment status, access to information, education level, geographical location and gender [26-28,]. A study in coastal Kenya [29] reported 60% knowledge of COVID-19 prevention measures, citing inequalities in knowledge, awareness and reach of health information.

The high awareness of COVID-19 reported in this study can largely be attributed to adoption of a whole of government and multi-agency approach (policy and technical) in response to the COVID-19 threat, accompanied by widespread media campaigns and news reports. A long history and experience with infectious disease response may have made messaging on hand hygiene and cough/sneeze etiquette more acceptable and readily implementable in Kenya. This however was not achieved with wearing masks and maintaining social distance as they were considered novel interventions. Lags in supply and affordability of masks and sanitising agents, and difficulties in sustaining lockdowns and movement restriction as reported in this study and elsewhere [14], contributed to failure in observing regular use of masks and social distancing. Low adherence to mask wearing and social distancing at a time of widespread consensus on COVID-19 being an airborne disease coupled with the low vaccination rate [16], call for innovative ways to engage communities in developing contextually relevant public health measures, particularly when

For peer review only - http://bmjopen.bmj.com/site/about/guidelines.xhtml

introducing novel interventions, cognisant of the influence of local culture and the impact of the socio-economic environment on the capacity for adherence. Likewise, emphasis on building community knowledge and not just providing top-down instructions, engaging communities in the adaptation processes as previously shown in Bangladesh [30], has the potential to improve adherence and ownership through better understanding. Further, the reported need for additional information on the disease indicates a lag in adjusting the communication messaging to the evolution of the pandemic, a gap that requires addressing.

Public health information

Our study found that community engagement with preventive measures improved with access to appropriate messages from preferred channels of communication. Socio-economic inequalities observed were slightly mitigated by trust placed in the source of information (religious leaders, WhatsApp, radio, and community health workers), signalling the opportunity to capitalize on the trust hierarchy to facilitate adherence to preventive behaviours. The pandemic communication strategy needs to be tailored to the local context [31], and the dynamics of information flow to address gaps in information penetration and engagement. Key in this process is the translation of the standardised pandemic language and procedures to local contexts through participatory processes. In addition, the reported community's loss of trust in the genuineness of the government efforts warrants a concerted effort to win back the public to comply with COVID-19 prevention, possibly through dialogue. The pastoralist communities require special attention as a key population due to their nomadic way of life that can marginalize them in respect of public health awareness and prevention measures and could increase the risk of cross-border transmission and potential recurrent outbreaks [15]. The risk is exacerbated by limited access to water and sanitation. There is need therefore for concerted effort in improving health literacy through tailored education campaigns and messaging in the border regions, and especially among the pastoralists, supported by structural interventions to enable disadvantaged and marginalised communities adhere to prevention measures.

An added dimension to risk communication is the stability and strength of mobile network coverage, which tends to be poorer in rural areas. Representativeness in sampling of participants in both counties could reflect the relative ease of access to local radio when compared to

For peer review only - http://bmjopen.bmj.com/site/about/guidelines.xhtml

television and internet-based social media, in contrast to other work done in urban settings in Kenya [26-27].

Border dynamics

Border health security is key to safeguarding the EAC's economic and social security. Studies show that implementation of COVID-19 interventions are likely problematic for populations with weak health systems, high density housing, porous borders and insecurity [32]. The disruptions to trade and livelihoods have had profound effects on border communities [33]. Concerns over financial stability (loss of income and food security) have persisted over time. There is a need to economically empower and build resilience among border communities to withstand the current and similar shocks in future. The border counties had to cater for a bigger population than their official figures, and as such, budgetary allocation to these counties should recognize this reality. The mandatory testing of truck drivers caused a major backlog in Busia which lies on the Northern Transport Corridor. As gathering points for large numbers of people, border crossings are inherently a risk point for transmission of SARS Cov2. This calls for better organization in designating and preparing holding areas in border regions. This could have potentially protected the local communities in the initial phase of COVID-19 and later with established community spread, the uninfected drivers.

In addition to the harmonised testing protocol of truckers by Kenya and her neighbours, there is also a need to strengthen cross-border surveillance networks to support contact tracing to increase the likelihood of containment of outbreaks and reduce the risk posed by cross-border health threats [34]. Adherence to prevention measures and movement restrictions at the borders were reportedly undermined by porous borders and strong social ties between the cross-border communities. The COVID-19 preventive measures and mitigation measures should thus be accompanied by intensive community engagement to minimise a sense of alienation among these communities. The use of informal crossings should be mitigated by developing criteria accessible to all that recognizes the inherent economic and social challenges in the region.

STUDY LIMITATIONS

The study did not objectively assess the knowledge of preventive behaviours, nor sought participants' self-reported levels of adherence. To overcome the potential bias of self-reported

For peer review only - http://bmjopen.bmj.com/site/about/guidelines.xhtml

information, qualitative interviews were used to explore factors influencing ability of the community to adhere to preventive measures which would be useful going forward than adherence reported at a single time point. Adopting standardized international research tools enables comparison of findings even though the tools do not comprehensively capture all possible sources of information, reflecting an issue of adapting Western-designed surveys. Whereas Kenya's median age is 21, majority of the e-survey participants were ≥55 years. This group tends to be less tech savvy. To minimise the digital divide and barriers to participant participation, the study trained CHWs, deployed a 'light' data collection tool and adaptive workflows, and provided robust technical support. The study recorded a lower number of Mandera interviews due to the geographical expansiveness of the area. However, the context-specific challenges presented what may be considered socio-culturally appropriate recommendations to inform future local efforts.

CONCLUSION

This research highlights the importance of pandemic response and communication in engagement with COVID-19 preventive measures in border settings. As in other contexts, socioeconomic inequalities were reflected in knowledge of and adherence to COVID-19 prevention measures, with the less educated and those in lower socioeconomic groups at greatest risk. The influence of trusted sources of information in mitigating such inequalities demonstrate the need for tailored public health communication strategies that are cognisant and responsive to local context and patterns of information flow. Further effort needs to be made in risk communication by modifying health messaging in tandem with evolution of the pandemic and incorporating community communication needs. Improving health literacy may support better health outcomes for the population.

Livelihood insecurity directly prevents the most disadvantaged from adhering to prevention measures. Economic empowerment and building resilience against future shocks is thus an important investment the country needs going forward. Coordinating response measures across border points is crucial in winning the community's trust and maintaining essential economic and social activities.

For peer review only - http://bmjopen.bmj.com/site/about/guidelines.xhtml

CONTRIBUTORS: KL, OJ, KA, MJ, CC, AI, LV, RU, DP, CK, ME and HS participated in conceiving the study, implementation and drafting of the manuscript. All authors contributed to the interpretation and subsequent revision of the paper. All authors approved the final version of the manuscript.

COMPETING INTERESTS: None

FUNDING: This work was supported by the Kenya Medical Research Institute (KEMRI) Internal Research Grant number KEMRI/COV/SPEC/002. HS, CK, RU funded by MR/N015959/1, HS, KA, KL funded by MR/S009035/1, HS funded by NIHR7\1004, LV was funded by DRF 2017-10-132; HS and DP were also funded by MR/R022739/1; DP funded by NIHR203318.

DATA SHARING AGREEMENT: All data relevant to the study are included in the article or uploaded as supplementary information.

ACKNOWLEDGEMENTS: We are grateful to all the study participants from Busia and Mandera Counties who took time to participate in this study. We thank the Director KEMRI, the National Commission for Science, Technology and Innovation, and the County Governments of Busia and Mandera for granting permission and providing an enabling environment to undertake this study. Many thanks to our research collaborators at Kings College London for their technical guidance and support, and the KEMRI County Cluster Coordinators Mr. Surrow Adow and Ms. Olipher Makwaga for their support. We thank our research assistants Schiller Mbuka, Rodgers Ochieng, Melvine Obuya, Esther Andia, Miriam Bosire, Doreen Mitaru and Priscilla Maiga for their tireless efforts in bringing the study to fruition.

ETHICS APPROVAL: The study protocol was approved by the KEMRI Scientific and Ethics Review Committee (Ref. No. KEMRI/SERU/CPHR/4021), and the National Commission for Science, Technology and Innovation (Ref. No. 864492).

REFERENCES

1. World Health Organization. WHO Coronavirus (COVID-19) Dashboard | WHO Coronavirus (COVID-19) Dashboard With Vaccination Data. https://covid19.who.int/region/afro/country/ke Accessed 20th January 2022.

For peer review only - http://bmjopen.bmj.com/site/about/guidelines.xhtml

2. Pinchoff J, Austrian K, Rajshekhar N, et al. Gendered economic, social and health effects of the COVID-19 pandemic and mitigation policies in Kenya: evidence from a prospective cohort survey in Nairobi informal settlements. *BMJ Open* 2021;11:e042749.
3. Brand SPC, Ojal J, Aziza R, et al. COVID-19 transmission dynamics underlying epidemic waves in Kenya. *Science* 2021;374:989-994.
4. World Bank poverty and equity data http://povertydata.worldbank.org/poverty/country/KEN Accessed 28th May 2020.
5. The COVID 19 global pandemic: Impact to the economy and policy options, Parliamentary Budget Office (PBO), Special Bulletin No. 1/2020, Government of Kenya (http://parliament.go.ke/sites/default/files/202004/Special%20Bulletin%20Covid%2019%202.4.2020%20final.pdf)
6. The Seventh Presidential Address on the Coronavirus Pandemic: The 8-Point Economic Stimulus Programme. Government of Kenya (https://www.president.go.ke/2020/05/23/the-seventh-presidential-address-on-the-coronavirus-pandemic-the-8-point-economic-stimulus-programme-saturday-23rd-may-2020/)
7. World Bank. 2021. Socioeconomic Impacts of COVID-19 in Kenya on Households: Rapid Response Phone Survey, Round 1. (https://openknowledge.worldbank.org/handle/10986/35173 License: CC BY 3.0 IGO)
8. Shadmi E, Chen Y, Dourado I, et al. Health equity and COVID-19: global perspectives. *International Journal for Equity in Health* 2020; 19:10
9. Quaife M, van Zandvoort K, Gimma A, et al. The impact of COVID-19 control measures on social contacts and transmission in Kenyan informal settlements. *BMC Medicine* 2020; 18:316.
10. Ope, M, Sonoiya S, Kariuki J, et al. Regional Initiatives in Support of Surveillance in East Africa: The East Africa Integrated Disease Surveillance Network (EAIDSNet) Experience. *Emerging Health Threats Journal* 2013; *6*.
11. Okongo, O. Malaria pandemic , leading cause of deaths in Busia County. *Talk Africa* 2017. https://www.talkafrica.co.ke/reports-indicate-malaria-cases-cause-deaths-busia-county/

For peer review only - http://bmjopen.bmj.com/site/about/guidelines.xhtml

12. Nnnennaya A, & Hagembe J. Increasing Access to Healthcare Services for Cross-Border and Mobile Communities in East Africa – Global Health Innovation Center, 2019. (https://dukeghic.org/2019/07/19/increasing-access-to-healthcare-services-for-cross-border-and-mobile-communities-in-east-africa/)
13. World Health Organisation. Horn of Africa countries in a communique to speed up polio eradication. Regional Office for Africa. 2018. https://www.afro.who.int/pt/node/10538
14. East African Community. Mapping of Health Services along major transport corridors in East Africa. East African Community Report, 2015.
15. African Union. The COVID-19 Pandemic in the IGAD region: Impact on the Health and Economy Vol 1. Horn Economic and Social Policy Institute (HESPI): Pillar 2 An assessment of the health and economic impact on the HoA countries, 2020. (https://media.africaportal.org/documents/The-COVID-19-Pandemic-in-the-IGAD-region_VOL-I.pdf)
16. Kenya Ministry of Health. COVID-19 outbreak in Kenya. Daily Situation Report 673. 19th January 2022.
17. Kenya Ministry of Health. Kenya Harmonized Health Facility Assessment Report (KHFA) 2018/2019 Report.
18. Creswell, J. W., & Plano Clark, V. L. (2011). Designing and conducting mixed methods research. Thousand Oaks, CA: Sage.
19. Kenya National Bureau of Statistics. Kenya Population and Housing Census. Volume IV: Distribution of Population by Socio-Economic Characteristics 2019 (https://www.knbs.or.ke/?wpdmpro=2019-kenya-population-and-housing-census-volume-iv-distribution-of-population-by-socio-economic-characteristics)
20. McBurney P. On transferring statistical techniques across cultures: The Kish Grid. Current Anthropology 1998; 29:323-325.
21. World Health Organization. Risk Communication and Community Engagement (RCCE) Action Plan Guidance COVID-19 Preparedness and Response, 2020 (https://www.who.int/publications/i/item/risk-communication-and-community-engagement-(rcce)-action-plan-guidance)

For peer review only - http://bmjopen.bmj.com/site/about/guidelines.xhtml

22. Harris PA, Taylor R, Thielke R, et al. Research electronic data capture (REDCap)—A metadata-driven methodology and workflow process for providing translational research informatics support. *Journal of Biomedical Informatics* 2009; 42: 377–381.

23. StataCorp. 2019. Stata Statistical Software: Release 16. College Station, TX: StataCorp LLC.

24. Zou G. A modified Poisson regression approach to prospective studies with binary data. *Am J Epidemiol.* 2004;159(7):702–6.

25. Gale NK, Heath G, Cameron E, et al. Using the framework method for the analysis of qualitative data in multi-disciplinary health research. *BMC Medical Research Methodology* 2013; 13:117

26. Karijo E, Wamugi S, Lemanyishoe S, et al. Knowledge, attitudes, practices, and the effects of COVID-19 among the youth in Kenya. *BMC Public Health* 2021; 21(1):1020.

27. Austrian K, Pinchoff J, Tidwell JB, et al. COVID-19 related knowledge, attitudes, practices and needs of households in informal settlements in Nairobi, Kenya. [Preprint]. *Bull World Health Organ*. E-pub: 6 April 2020. doi: http://dx.doi.org/10.2471/BLT.20.260281

28. Sengeh P, Jalloh MB, Webber N, et al. Community knowledge, perceptions and practices around COVID-19 in Sierra Leone: a nationwide, crosssectional survey. *BMJ Open* 2020;10:e040328.

29. Mwai J, Mutai J, Kaduka L, et al. Association between sociodemographic characteristics and knowledge and practice of COVID-19 measures among households in Mombasa and Kilifi County, Kenya. *Int Health*. 2022:ihac049.

30. Abaluck J, Kwong LH, Styczynski A, et al. Impact of community masking on COVID-19: A cluster-randomized trial in Bangladesh. *Science* 2022;14;375(6577):eabi9069.

31. Sachs JD, Karim SSA, Aknin L, et al. The Lancet Commission on lessons for the future from the COVID-19 pandemic. *Lancet*. 2022;400:1224-1280.

32. Da'ar OB, Haji M & Jradi H. Coronavirus Disease 2019 (COVID-19): Potential implications for weak health systems and conflict zones in the Middle East and North Africa region. *Int J Health Plann Manage* 2020; 35(5):1240-1245.

For peer review only

For peer review only - http://bmjopen.bmj.com/site/about/guidelines.xhtml

33. Quaife M, van Zandvoort K, Gimma A, et al. The impact of COVID-19 control measures on social contacts and transmission in Kenyan informal settlements. *BMC Medicine* 2020; 18:316.

34. East African Community. Standard Operating Procedure - Cross-border Surveillance in the EAC Region East African Community (EAC) Secretariat. www.eac.int

For peer review only

Figure Legends

Figure 1: Map showing the location of Busia and Mandera Counties in Kenya

For peer review only - http://bmjopen.bmj.com/site/about/guidelines.xhtml

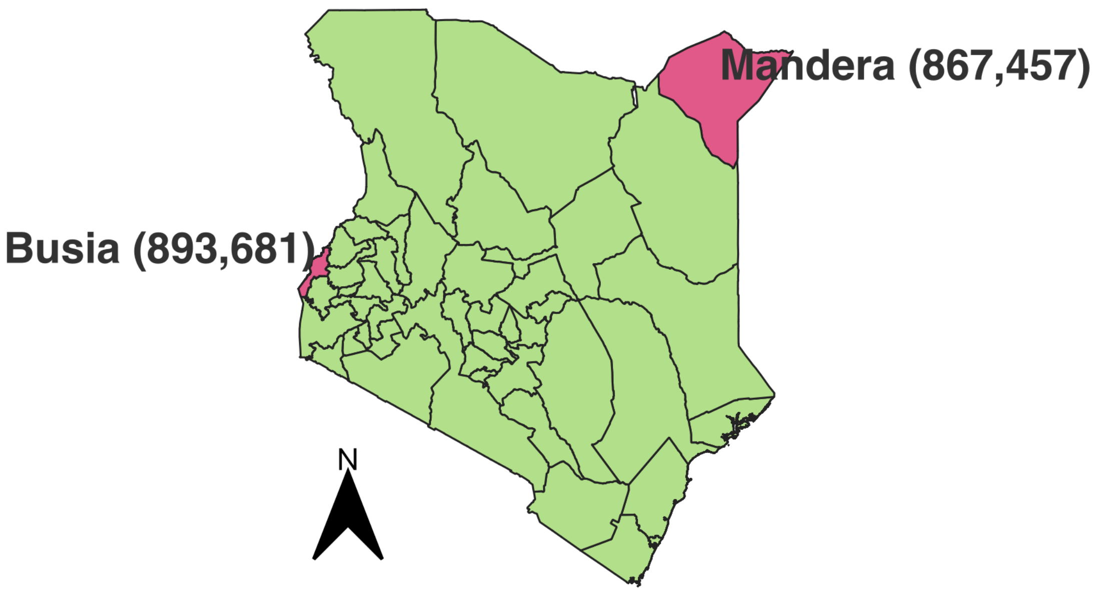
Mandera (867,457)
Busia (893,681)
N

COVID-19 RAPID QUANTITATIVE ASSESSMENT TOOL

ID of Study Participant: ……………………………………………
Date of Interview: ……………………………………………….
Physical Location: ……………………………………………….

**1. SOCIO-DEMOGRAPHIC INFORMATION**		
1.1	Sex	[1] Male [2] Female
1.2	Age of respondent (in complete years)	
1.3	What is your current marital status?	[1] Single (not married and not living with a partner)<br>[2] married (monogamous)<br>[3] married (polygamous)<br>[4] Separated (currently not living together but not divorced)<br>[5] Divorced<br>[6] Widowed/widower<br>[7] Co-habiting (not married but live with a partner)
1.4	What level of education or years of schooling did you finish?	[1] None<br>[2] Primary education, 1-4 years<br>[3] Primary education, 5-8 years<br>[4] Secondary Education, 9-12 years<br>[5] Secondary Education, 13-14<br>[6] University Education > 14 years<br>[7] Adult education
1.5	What is your main occupation?	[1] Student<br>[2] Employed/Professional(Teacher, doctor, nurse, manager, accountant)<br>[3] Casual worker (attendant in hotel, bar, shop, housemaid, gardener)<br>[4] Housewife<br>[5] Business (self-employed)<br>[6] Farmer (large scale, subsistent farming, gardening)<br>[7] Unemployed<br>[8]Other(specify) ________________________________
**2. SOCIO-ECONOMIC INFORMATION**		
2.1	Currently, who are you living with? (*Circle all that apply)*	[1] Alone<br>[2] Parents<br>[3] Spouse<br>[4] Children<br>[5] Other relatives<br>[6] Friends
2.2	What is the **MAIN source** of income for your household? (*Circle only one)*	[1] Formal employment<br>[2] Small scale farming<br>[3] Farming and selling of produce<br>[4] Small business, self-employed (non-farming)<br>[5] Petty trade<br>[6] Casual/informal labour<br>[7] Relatives/remittances<br>[8] Welfare/NGO support<br>[9] Other (specify)________________________________
2.3	What is the **MAIN** source of food for your	[1] Purchase (market/grocery store)<br>[2] Household farm/garden

For peer review only - http://bmjopen.bmj.com/site/about/guidelines.xhtml

	household? (*Choose one response only*)	[3] Relatives and friends<br>[4] Welfare/NGO support<br>[5] Other (specify) _____________<br>[88] Don't Know
2.4	How much on average do you spend on food in your household? (*choose from categories*)	[1] Less than 1000<br>[2] 1,000-2,999<br>[3] 3,000-4,999<br>[4] 5,000-9,999<br>[5] 10,000-19,999<br>[6] 20,000-49,999<br>[7] 50,000- 99,999<br>[8] 100, 000+
2.5	What is the main source of your domestic water?	[1] Tap/rain water within compound (piped into the house)<br>[2] Tap water outside the house compound (public tap)<br>[3] Tap water, purchased from trolley (mkokoteni)<br>[4] Well/bore hole<br>[5] Pond water<br>[6] Other (specify) ____<br>[88] Don't Know
2.6	Do you usually treat water for drinking?	[1] Yes<br>[2] No<br>[88] Don't know
2.7	If **YES to 3.6**, how do you usually treat water for drinking?	[1] Boil<br>[2] Filter<br>[3] Boil and Filter<br>[4] Use bottled water<br>[5] Do nothing<br>[6] Other (specify) ____________________________________<br>[88] Don't Know
2.8	What kind of latrine/toilet facility do members of your household usually use?	[1] Flush or pour flush toilet<br>[2] Pit latrine<br>[3] Bucket toilet<br>[4] Hanging toilet/hanging latrine<br>[5] No facility/bush/field<br>[6]Other (Specify)____
2.9	Do you share this toilet facility with other households?	[1] Yes<br>[2] No
2.10	Does your household have *(tick as many)*	[1] Clock/watch<br>[2] Electricity<br>[3] Radio<br>[4] Television<br>[5] Mobile telephone<br>[6] Fixed telephone<br>[7] Refrigerator<br>[8] Solar panel
2.11	Does any member of your household own:	[1] Bicycle<br>[2] Motorcycle/scooter<br>[3] Animal-drawn cart<br>[4] Car/truck

For peer review only - http://bmjopen.bmj.com/site/about/guidelines.xhtml

		[5] Boat with motor
2.12	What type of fuel does your household **MAINLY** use for cooking?	[1] Firewood<br>[2] Charcoal<br>[3] Kerosene<br>[4] Electricity<br>[5] Gas<br>[88] Other (Specify)________________________________
2.13	The house you live is it,	[1] Rented<br>[2] Live without pay?<br>[3] Owns<br>[4] Squatting
2.14	How many sleeping rooms are there in the house you live?	[1] Zero or one sleeping rooms<br>[2] Two sleeping rooms<br>[3] Three or more sleeping rooms.
2.15	What quality /type of material is the floor of the house you live in?	[1] Finished floor with parquet, carpet, tiles, ceramic Tiles<br>[2] Cement, concrete, raw wood, etc<br>[3] None, earth, dung Wood<br>[4] Other (specify) ________________________
**3. KNOWLEDGE ABOUT COVID-19**		
3.1	Have you heard about the new Coronavirus disease (COVID-19)	[1] Yes<br>[2] No
3.2	What do you know about the new coronavirus disease? (Choose all that apply)	Choose one best answer:<br>**[1]** I don't know anything<br>**[2]** It's a virus that can cause a disease<br>**[3]** It's a government's programme<br>**[4]** It's a TV/radio campaign<br>**[5]** Other: Specify________________
3.3	What kind of information have you received about the disease? (Legends to be added to the questionnaire)	Mark all answers that are true:<br>**[1]** How to protect yourself from the disease?<br>**[2]** Symptoms of the new coronavirus disease<br>**[3]** How it is transmitted<br>**[4]** What to do if you have the symptoms<br>**[5]** People who are at high risk of getting the disease<br>**[6]** Complications of the disease<br>**[7]** Other (please specify)_________

For peer review only

For peer review only - http://bmjopen.bmj.com/site/about/guidelines.xhtml

3.4	Where did you hear about the new coronavirus disease from? What channels or sources? (Tick all that apply)	Mark all channels that you heard about the disease from:<br>**[1]** Radio<br>**[2]** TV<br>**[3]** WhatsApp<br>**[4]** Social Media (e.g. Facebook, Instagram, Twitter)<br>**[5]** Health unit/Healthcare worker<br>**[6]** Family members<br>**[7]** Friends<br>**[8]** Community health workers<br>**[9]** Red Cross/ Red Crescent volunteers<br>**[10]** Other community mobilisers<br>[11] Community leaders<br>[12] Religious Leaders<br>[13] Traditional Healers<br>[14] Traditional Midwives<br>[15] Any other person from the community<br>[16] Other (please specify) __________
3.5	Which channel/who do you trust the most to receive information related to coronavirus? (one or more options)	Check the best channel for you to receive coronavirus information from:<br>**[1]** Radio<br>**[2]** TV<br>**[3]** WhatsApp<br>**[4]** Social Media (not WhatsApp)<br>**[5]** Health unit/Healthcare worker<br>**[6]** Family members<br>**[7]** Friends<br>**[8]** Community health workers<br>**[9]** Red Cross Red Crescent volunteers<br>**[10]** Other community mobilisers<br>[11] Community leaders<br>[12] Religious Leaders<br>[13] Traditional Healers<br>[14] Traditional Midwives<br>[15] Any other person from the community<br>[16] Other (Specify) ______________________
3.6	How dangerous do you think the new coronavirus risk is?	Choose the best answer that matches your belief about the disease:<br>**[1]** Very dangerous<br>**[2]** More or less dangerous<br>**[3]** Is not dangerous<br>**[4]** Other (Specify)________________________
3.7	Who do you think is at highest risk to get the coronavirus?	

For peer review only - http://bmjopen.bmj.com/site/about/guidelines.xhtml

		Mark all those you think are likely to become sick:<br>**[1]** Children under 5 years old<br>**[2]** Adolescents up to 15 years old<br>**[3]** Youth<br>**[4]** Adults<br>**[5]** Elderly persons<br>**[6]** Pregnant women<br>**[7]** Health workers<br>**[8]** Persons with chronic diseases such as diabetes and high blood pressure<br>**[9]** Other (Specify)_________________
3.8	Do you think you are likely to become sick with the new coronavirus?	[1] Yes<br>[2] No<br>[3] Don´t know<br>If Yes or No, tell us Why_
3.9	Do you think any of your family members are at risk of becoming sick with coronavirus?	[1] Yes<br>[2] No<br>[3] Don´t know<br>If Yes or No, tell us Why_
3.10	How does the coronavirus spread?	Mark all the ways you think the disease spreads:<br>**[1]** Blood transfusion<br>**[2]** Droplets from infected people<br>**[3]** Airborne<br>**[4]** Direct contact with infected people.<br>**[5]** Touching contaminated objects/surfaces<br>**[6]** Sexual intercourse contact<br>**[7]** Contact with contaminated animals<br>**[8]** Mosquito bites<br>**[9]** Eating contaminated food<br>**[10]** Drinking unclean water<br>**[11]** Don't know<br>[12] Other (Specify)_________________
3.11	What are the main symptoms of COVID-19?	Mark all the symptoms you think are caused by the new coronavirus:<br>**[1]** Fever<br>**[2]** Cough<br>**[3]** Shortness of breath and breathing difficulties<br>**[4]** Muscle pain<br>**[5]** Headache<br>**[6]** Diarrhoea<br>**[7]** Don't know<br>**[8]** No symptoms<br>**[9]** Other (Specify) _________________________

For peer review only - http://bmjopen.bmj.com/site/about/guidelines.xhtml

3.12	Do you know how to prevent it? (One or more options)	Choose all the prevention steps you know about:<br>**[1]** Sleep under the mosquito net<br>**[2]** Wash your hands regularly using soap and water<br>[3] Use hand sanitiser<br>**[4]** Drink only treated water<br>**[5]** Cover your mouth and nose when coughing or sneezing<br>**[6]** Avoid close contact with anyone who has a fever and cough<br>**[7]** Eliminate standing water<br>**[8]** Cook meat and eggs well<br>**[9]** Avoid unprotected direct contact with live animals and surfaces in contact with animals<br>**[10]** Standing 2 metres away from other people<br>**[11]** Wearing a mask<br>**[12]** Don't know<br>**[13]** Other Specify___________________________
3.13	What have you and your family been doing to prevent becoming sick with coronavirus in the recent days? (Tick all that apply)	Mark all steps that you have taken:<br>**[1]** Washing hands regularly using soap / water<br>[2] Using alcohol-based hand sanitiser<br>**[3]** Covering mouth and nose when coughing or sneezing<br>**[4]** Avoid close contact with anyone who has a fever and cough<br>**[5]** Eliminate standing water<br>**[6]** Cook meat and eggs well<br>**[7]** Avoid unprotected direct contact with live animals and surfaces in contact with animals.<br>**[8]** Standing 2 metres away from other people<br>**[9]** Wearing a mask<br>**[10]** Don't know<br>**[11]** Nothing<br>**[12]** Other (Specify)___________________________________
3.14	If Nothing to question 3.13, what makes it difficult for you to follow advice on preventing yourself from the disease?	**[1]** Need to go to work to earn money<br>**[2]** Live with a large family in a small house<br>**[3]** Don't know<br>**[4]** Other (Please specify)______________________
3.15	Do you consider it important to take actions to prevent the spread of coronavirus in your community?	[1] Yes<br>[2] No<br>[3] Don't know<br>[4] Other (Specify) ________________________
3.16	What would you do if you or someone from your family has symptoms of this disease?	

For peer review only - http://bmjopen.bmj.com/site/about/guidelines.xhtml

		Tick all the actions you would take:<br>**[1]** I will look for a more experienced relative to advise me on what to do<br>**[2]** I will go to the hospital / health unit<br>**[3]** I will go to the neighbourhood nurse<br>**[4]** I will buy medicines at the market<br>**[5]** I will look for the traditional healer<br>**[6]** I would stay in quarantine<br>**[7]** Other (Specify) ______________________________
3.17	What more would you like to know about the disease?	Choose all topics that you would like to know about;<br>**[1]** How to protect yourself from the disease?<br>**[2]** Symptoms of the new coronavirus disease<br>**[3]** How it is transmitted<br>**[4]** What to do if you have the symptoms<br>**[5]** Most at risk groups<br>**[6]** How to treat it<br>**[7]** Other (Specify)___________________________
3.18	Do you think the coronavirus disease is generating stigma against specific people?	[1] Yes<br>[2] No<br>[3] Don't know
3.19	(If yes) Which group is being dis-criminated in your community because of coronavirus?	1.____<br>2.____<br>3.____
3.20	Do you think Kenya is adequately prepared in dealing with this disease?	[1] Yes<br>[2] No<br>[3] Don't know
3.21	Do you know of any special measures that have been put in place nationally to deal with this new coronavirus? (Tick all that apply)	[1] Social distancing<br>[2] Closing borders<br>[3] Coronavirus telephone line/help<br>[4] Temporary expansion of healthcare facilities<br>[5] National Curfew<br>[6] Other (please specify)
3.22	If restrictions are in place, have you had difficulty in acquiring essential supplies such as food?	[1] Yes<br>[2] No<br>[3] Don't know
3.23	How do you think vulnerable groups can access essential supplies such as food and medicine? (Tick all that apply)	(1). Friends and family<br>(2). Community programmes<br>(3). National government run service<br>(4). NGO<br>(5). Religious organisations<br>(6). Other (please specify)

For peer review only

For peer review only - http://bmjopen.bmj.com/site/about/guidelines.xhtml

4. KNOWLEDGE ABOUT SIMILAR DISEASES: SEASONAL OR REGULAR FLU		
4.1	What do you usually do if you have a regular flu?	Mark the 1-2 most typical actions you take:<br>**[1]** I have never had the flu<br>**[2]** Nothing, I continue with my normal life, after some time the flu goes away<br>**[3]** I stay at home, so I don't infect others<br>**[4]** I wear a mask<br>**[5]** I drink tea until I recover<br>**[6]** I seek advice from my neighbours<br>**[7]** I seek advice from local healers<br>**[7]** I go to the health unit<br>**[8]** Other (Specify) ________________________________
4.2	From where/who do you usually receive information on flu and other diseases?	Choose as many channels as you have heard about regular flu from:<br>**[1]** Radio<br>**[2]** TV<br>**[3]** WhatsApp<br>**[4]** Social Media (not WhatsApp)<br>**[5]** Health unit/Healthcare worker<br>**[6]** Family members<br>**[7]** Friends<br>**[8]** Community health workers<br>**[9]** Red Cross Red Crescent volunteers<br>**[10]** Other community mobilisers<br>[11] Community leaders<br>[12] Religious Leaders<br>[13] Traditional Healers<br>[14] Traditional Midwives<br>[15] Any other person from the community<br>[16] Other (Specify) ________________

THANK YOU VERY MUCH FOR YOUR TIME

For peer review only - http://bmjopen.bmj.com/site/about/guidelines.xhtml

COVID-19 QUALITATIVE INTERVIEW GUIDE

This will be a semi structured interview with people who have knowledge of and experienced the COVID-19 disease. We wish to obtain views/opinions from policy actors, healthcare workers, COVID-19 survivors and carers, COVID-19 bereaved, truckers and traders.

Introductory Script

Enumerator:
Good morning/ afternoon,
My name is {**Enumerator Name**} from **KEMRI**.
Welcome to this session and thank you for taking the time to be here. We are conducting a research study on behalf of KEMRI and Kings College London (UK) to assess the response strategies and community/practitioner experiences of COVID-19 in border counties in Kenya.

We would like you to be as honest and as open as you can. Also take note that there are no correct, right or wrong response(s) to the issues raised. Feel free to chip in or interject at any point. All your responses will be both confidential and anonymous, and your name and details will not be divulged during analysis. Your responses will not affect your standing with anyone or any organization/company mentioned in this discussion.

In order to make sure that we capture the most useful account of this discussion, we would like to audio record our conversation. This recording will only be available to the research team involved. Will you give us permission to record?
Thank you.

Demographic Information

1. Participant geographic Location
2. Sex
3. Age
4. Occupation
5. Level of Education

Awareness of COVID-19

1. Have you heard about the New Coronavirus Disease (COVID-19)? Please tell me what you know about it (*Explain to the participant what the disease is in case s/he doesn't know what it is*)
2. From whom/ where did you get this information from (*Prompt for details of the information given through the specified source e.g. symptoms, transmission, self-care, risks, complications etc.*)

For peer review only - http://bmjopen.bmj.com/site/about/guidelines.xhtml

3. Are there some people, information sources or channels that you trust more than others to give you accurate information about the disease? Which people or sources are these and why do you trust them?

4. In your view how dangerous do you think the COVID-19 disease is? (*Probe for reasons as to why*)

5. Who in your view do you think is most at risk of contracting this disease? (*Probe for reasons as to why*)

6. Please explain to me how you think the COVID-19 is spread in in your County?

7. In your view, how would you identify someone who is infected with the disease? (*Probe for main symptoms*)

8. Do you think a person can be infected and spread the infection to others before they start showing the symptoms you have just mentioned? (*probe for reason for their opinion*)

9. Have you heard of any test that is done by medical professionals to identify people who have the disease? (*probe for what they know about testing*)

10. How would you prevent yourself or your family from contracting this disease?

11. What have you been doing to prevent yourself and your family from contracting the disease? (*Probe for prevention measures*)

12. What role can you play to prevent the spread of the disease within your community? (*Probe for self-quarantine measures, creation of awareness*)

13. What would you do if a member of your family contracted the disease?

14. Where do you think would be the best place for your family member to receive treatment if they contracted the disease? If it is a place away from home, would you be able to take them there? (*probe for why this is so both yes and no answers*)

15. What myths and misconceptions have you heard about the disease? (Probe for myths and misconceptions around symptoms, transmission, prevention, treatment)

16. Is there any more information you would like to know about the disease? (*Probe for preferred source for the additional information*)

17. In your view, do you think the COVID-19 disease pandemic is generation stigma against specific people? (*Probe the specific groups of people being stigmatised and how they are being stigmatized*)

18. Have you ever been sick with seasonal or regular flu? What do you usually do when you have a seasonal flu? (*Probe for home remedies, seeking care from hospital, doing nothing about it etc.*)

For peer review only - http://bmjopen.bmj.com/site/about/guidelines.xhtml

Additional questions for policy actors and healthcare workers

19. What is your role in the current organization that you serve?

20. How would you describe the preparedness and response of Kenya's healthcare system to COVID 19?

21. What has been your personal experience in regard to COVID-19?

22. What are some of the facilitators and barriers in the healthcare system when it comes to dealing with COVID-19 pandemic?

23. What would you wish to see done differently?

Thank you very much for your time and response.

For peer review only

For peer review only - http://bmjopen.bmj.com/site/about/guidelines.xhtml

Supplementary Table 1: List of variables used for constructing a wealth index

	Name of Variable	Coding		Factor loadings	
				Factor1	Factor2
1	Ownership of household goods - 1 Clock/Watch	1 = Yes	0 = No		
2	Ownership of household goods - 2 Electricity	1 = Yes	0 = No	.708	
3	Ownership of household goods - 3 Radio	1 = Yes	0 = No		.657
4	Ownership of household goods - 4 Television	1 = Yes	0 = No	.628	.427
5	Ownership of household goods - 5 Mobile telephone	1 = Yes	0 = No	.375	
6	Ownership of household goods - 6 Fixed telephone	1 = Yes	0 = No		
7	Ownership of household goods - 7 Refrigerator	1 = Yes	0 = No		
8	Ownership of household goods - 8 Solar panel	1 = Yes	0 = No		.552
9	Any household member own - 1 Bicycle	1 = Yes	0 = No		.753
10	Any household member own - 2 Motorcyle/Scotter	1 = Yes	0 = No	.427	
11	Any household member own - 3 Animal drawn cart	1 = Yes	0 = No		
12	Any household member own - 4 Car/Truck	1 = Yes	0 = No		
13	Any household member own - 5 Boat with motor	1 = Yes	0 = No		
14	Own house live in	1 = Yes	0 = No		.346
15	Sleeping rooms in the house that live in	1, 2, 3		.640	
16	Finished/cement floor	1 = Yes	0 = No	.681	
	Small coefficients below .30 were suppressed<br>*Eigen values for components one and two were 2.464 & 1.509*				

For peer review only

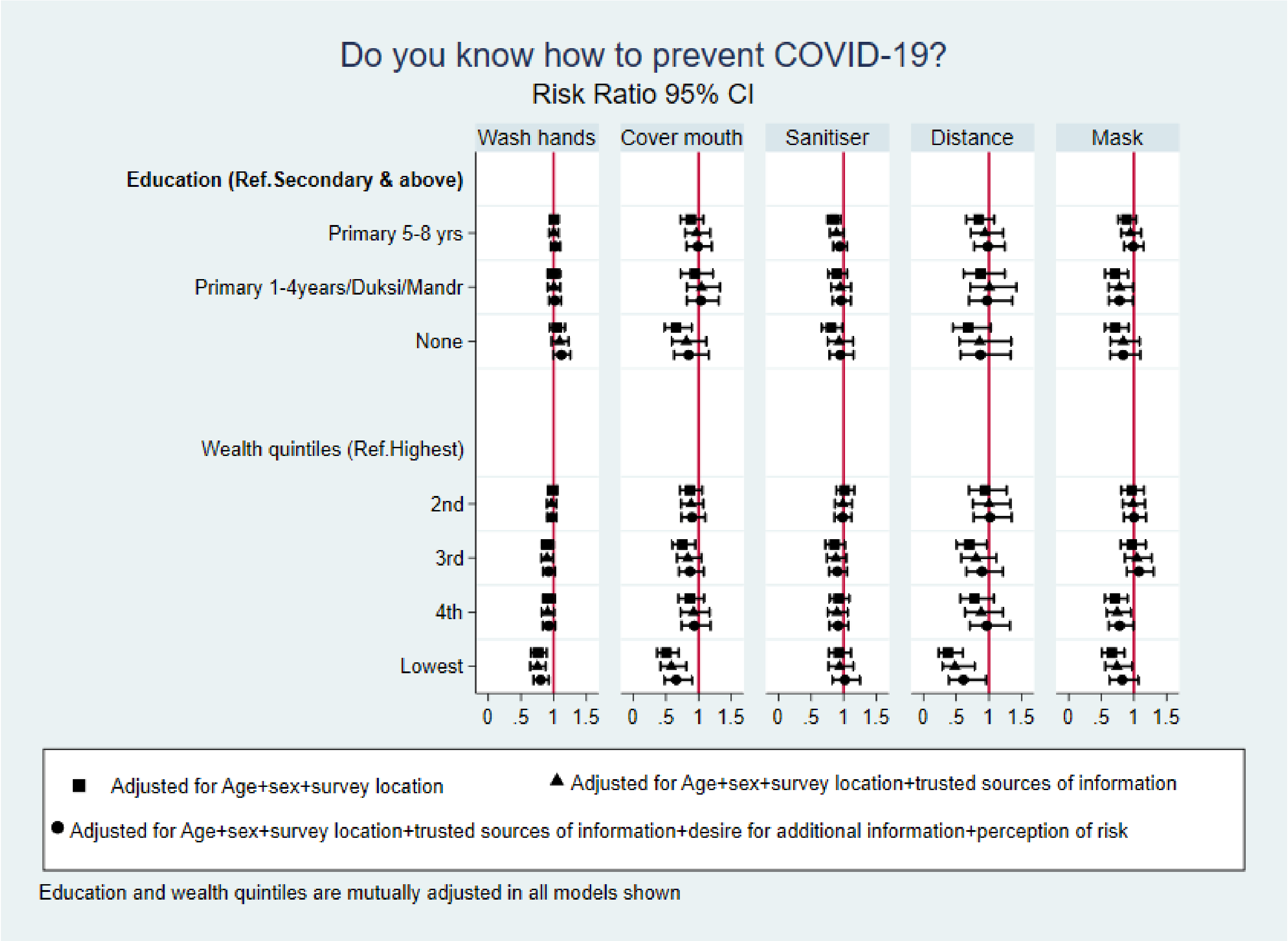

Supplementary Figure 1: Association between knowledge of prevention behaviours and measures of education and wealth. Risk Ratio and 95% confidence intervals.

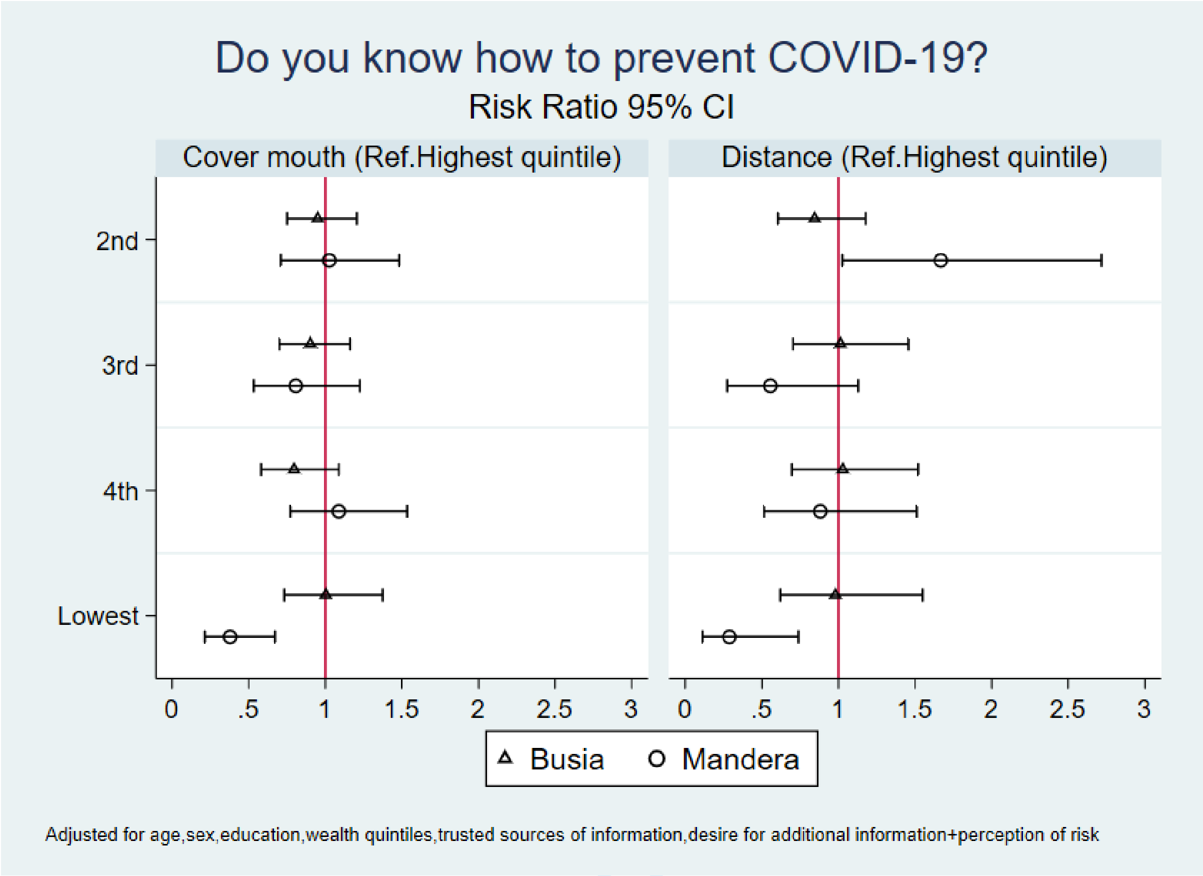

Supplementary Figure 2: Interaction between wealth quintiles and survey area for knowing that covering mouth when coughing/sneezing and keeping 2 metres distance could prevent COVID-19.

STROBE 2007 (v4) checklist of items to be included in reports of observational studies in epidemiology*

Checklist for cohort, case-control, and cross-sectional studies (combined)

Section/Topic	Item #	Recommendation	Reported on page #
**Title and abstract**	1	(*a*) Indicate the study's design with a commonly used term in the title or the abstract	1
		(*b*) Provide in the abstract an informative and balanced summary of what was done and what was found	3
**Introduction**			
Background/rationale	2	Explain the scientific background and rationale for the investigation being reported	4
Objectives	3	State specific objectives, including any pre-specified hypotheses	5
**Methods**			
Study design	4	Present key elements of study design early in the paper	5
Setting	5	Describe the setting, locations, and relevant dates, including periods of recruitment, exposure, follow-up, and data collection	5
Participants	6	(*a*) *Cohort study*—Give the eligibility criteria, and the sources and methods of selection of participants. Describe methods of follow-up<br>*Case-control study*—Give the eligibility criteria, and the sources and methods of case ascertainment and control selection. Give the rationale for the choice of cases and controls<br>*Cross-sectional study*—Give the eligibility criteria, and the sources and methods of selection of participants	5, 7
		(*b*) *Cohort study*—For matched studies, give matching criteria and number of exposed and unexposed<br>*Case-control study*—For matched studies, give matching criteria and the number of controls per case	
Variables	7	Clearly define all outcomes, exposures, predictors, potential confounders, and effect modifiers. Give diagnostic criteria, if applicable	6
Data sources/ measurement	8*	For each variable of interest, give sources of data and details of methods of assessment (measurement). Describe comparability of assessment methods if there is more than one group	6
Bias	9	Describe any efforts to address potential sources of bias	6
Study size	10	Explain how the study size was arrived at	5,7
Quantitative variables	11	Explain how quantitative variables were handled in the analyses. If applicable, describe which groupings were chosen and why	6
Statistical methods	12	(*a*) Describe all statistical methods, including those used to control for confounding	7
		(*b*) Describe any methods used to examine subgroups and interactions	7
		(*c*) Explain how missing data were addressed	7
		(*d*) *Cohort study*—If applicable, explain how loss to follow-up was addressed<br>*Case-control study*—If applicable, explain how matching of cases and controls was addressed	7

For peer review only

		Cross-sectional study—If applicable, describe analytical methods taking account of sampling strategy	
		(*e*) Describe any sensitivity analyses	7
**Results**			
Participants	13*	(a) Report numbers of individuals at each stage of study—eg numbers potentially eligible, examined for eligibility, confirmed eligible, included in the study, completing follow-up, and analysed	8
		(b) Give reasons for non-participation at each stage	
		(c) Consider use of a flow diagram	8
Descriptive data	14*	(a) Give characteristics of study participants (eg demographic, clinical, social) and information on exposures and potential confounders	10
		(b) Indicate number of participants with missing data for each variable of interest	10
		(c) *Cohort study*—Summarise follow-up time (eg, average and total amount)	
Outcome data	15*	*Cohort study*—Report numbers of outcome events or summary measures over time	
		Case-control study—Report numbers in each exposure category, or summary measures of exposure	
		Cross-sectional study—Report numbers of outcome events or summary measures	10
Main results	16	(*a*) Give unadjusted estimates and, if applicable, confounder-adjusted estimates and their precision (eg, 95% confidence interval). Make clear which confounders were adjusted for and why they were included	10
		(*b*) Report category boundaries when continuous variables were categorized	10
		(*c*) If relevant, consider translating estimates of relative risk into absolute risk for a meaningful time period	
Other analyses	17	Report other analyses done—eg analyses of subgroups and interactions, and sensitivity analyses	11-12
**Discussion**			
Key results	18	Summarise key results with reference to study objectives	15
Limitations	19	Discuss limitations of the study, taking into account sources of potential bias or imprecision. Discuss both direction and magnitude of any potential bias	4
Interpretation	20	Give a cautious overall interpretation of results considering objectives, limitations, multiplicity of analyses, results from similar studies, and other relevant evidence	15-17
Generalisability	21	Discuss the generalisability (external validity) of the study results	15-17
**Other information**			
Funding	22	Give the source of funding and the role of the funders for the present study and, if applicable, for the original study on which the present article is based	18

*Give information separately for cases and controls in case-control studies and, if applicable, for exposed and unexposed groups in cohort and cross-sectional studies.

Note: An Explanation and Elaboration article discusses each checklist item and gives methodological background and published examples of transparent reporting. The STROBE checklist is best used in conjunction with this article (freely available on the Web sites of PLoS Medicine at http://www.plosmedicine.org/, Annals of Internal Medicine at http://www.annals.org/, and Epidemiology at http://www.epidem.com/). Information on the STROBE Initiative is available at www.strobe-statement.org.

For peer review only
